# Time-course human urine proteomics in space-flight simulation experiments

**DOI:** 10.1186/1471-2164-15-S12-S2

**Published:** 2014-12-19

**Authors:** Hans Binder, Henry Wirth, Arsen Arakelyan, Kathrin Lembcke, Evgeny S Tiys, Vladimir A Ivanisenko, Nikolay A Kolchanov, Alexey Kononikhin, Igor Popov, Evgeny N Nikolaev, Lyudmila Kh Pastushkova, Irina M Larina

**Affiliations:** 1 Interdisciplinary Centre for Bioinformatics, Universität Leipzig, Leipzig, Germany; 2 Institute of Molecular Biology NAS RA; Yerevan, Armenia; 3 Institute of Cytology and Genetics SB RAS, Novosibirsk, Russia; 4 Talrose Institute for Energy Problems of Chemical Physics, RAS, Moscow, Russia; 5 Emanuel Institute for Biochemical Physics, RAS, Moscow, Russia; 6 Moscow Institute of Physics and Technology, Dolgoprudnyi, Russia; 7 Skolkovo Institute of Science and Technology, Skolkovo, Russian Federation; 8 Institute of Biomedical Problems - Russian Federation State Scientific Research Center RAS, Moscow, Russia

## Abstract

**Background:**

Long-term space travel simulation experiments enabled to discover different aspects of human metabolism such as the complexity of NaCl salt balance. Detailed proteomics data were collected during the Mars105 isolation experiment enabling a deeper insight into the molecular processes involved.

**Results:**

We studied the abundance of about two thousand proteins extracted from urine samples of six volunteers collected weekly during a 105-day isolation experiment under controlled dietary conditions including progressive reduction of salt consumption. Machine learning using Self Organizing maps (SOM) in combination with different analysis tools was applied to describe the time trajectories of protein abundance in urine. The method enables a personalized and intuitive view on the physiological state of the volunteers. The abundance of more than one half of the proteins measured clearly changes in the course of the experiment. The trajectory splits roughly into three time ranges, an early (week 1-6), an intermediate (week 7-11) and a late one (week 12-15). Regulatory modes associated with distinct biological processes were identified using previous knowledge by applying enrichment and pathway flow analysis. Early protein activation modes can be related to immune response and inflammatory processes, activation at intermediate times to developmental and proliferative processes and late activations to stress and responses to chemicals.

**Conclusions:**

The protein abundance profiles support previous results about alternative mechanisms of salt storage in an osmotically inactive form. We hypothesize that reduced NaCl consumption of about 6 g/day presumably will reduce or even prevent the activation of inflammatory processes observed in the early time range of isolation. SOM machine learning in combination with analysis methods of class discovery and functional annotation enable the straightforward analysis of complex proteomics data sets generated by means of mass spectrometry.

## Introduction

The physiological impact of human space flights missions exceeding several weeks poses problems such as radiation exposure, immunological depression and stress. Part of the concerns occur during the course of a mission, while others - such as cardiovascular deconditioning, bone and muscle losses and orthostatic intolerance - manifest themselves mainly upon return to earth only. These in-flight and post-flight physiological issues are vital to develop a sustainable program of human space exploration. Long-term space travel simulation experiments on earth are performed to discover the particular factors causing physiological and psychological problems and to develop methods helping to prevent or, at least to counteract them.

An interesting line of investigation was pursued as 'Mars isolation study' conducted at the Institute of Biomedical Problems in Moscow to simulate a journey to our neighbor planet. The purpose was to find out more about the effects of a long period of isolation on the human physiological and mental conditions in terms of data gathered over several weeks. Volunteers were confined to an enclosed, restricted environment where they obtained diets with defined amounts of salt (NaCl) and micro elements content and performed different activity programs. These studies were remarkable for their sustained duration and tight control of environmental variables. This ground-based space station model experiment enabled a novel, profound and extended trip to our 'inner space' to discover new aspects of human metabolism [1].

Particularly, the study provided a unique and detailed profile of physiological responses to decreasing salt intake. Besides playing a part in the development of hypertension (an actual study estimates that more than 1.5 million annual deaths from cardiovascular causes worldwide were attributed to increased sodium consumption [2]) and the weakening of the immune system, too much salt also seems to have a negative effect on the musculo-skeletal system due to acidification caused by the binding of salt to sugar-protein compounds. In consequence a high salt intake increases bone and muscle loss in humans on earth which is even exacerbated in the absence of gravity. One expects that a salt-reduced diet possibly diminishes negative effects such as bone degradation in space flights.

Although the physiology of salt balance is well understood (see the short review in [1] and the references cited therein) the space flight simulation experiment highlighted a new complexity in physiological responses that cannot be easily explained by previous knowledge [3-5]. For example, the studies raised doubts about the strict link between salt and water balance which are presumably caused by the storage of NaCl in a molecularly-bound, osmotically-inactive form paralleled by immune system driven micro-vascularization in skin which tends to reduce blood pressure [6-8].

One needs further exploration of these findings to improve our understanding of the effect of diet and of isolation on human physiology especially to understand the regulatory modes on the molecular level. So far measures estimating the kinetics of salt balance and of hormone production were analyzed and related to global parameters such as the blood pressure, extracellular water and body weight [3-5]. In addition to these measures, detailed urine proteomics data were collected during the Mars105 isolation experiment lasting 105 days potentially enabling a deeper insight into the molecular processes involved. First analyses report a high variability of protein abundance identified in the urine samples [9]. Another analysis established associations between clusters of proteins and a functional protein networks related to sodium intake which has been extracted from literature using bioinformatics methods [10]. A third study analyzed the possible tissue origin of the proteins detected. It founds an increased number of renal and urinary tract proteins after a real space mission compared with the ground-based flight simulation presumably reflecting the accumulation of sodium in cosmonauts body during space missions [11].

A comprehensive analysis of the time-dependent urine proteomics data set collected during the ground based flight simulation is still pending. In this publication we analyze the abundance of the about two thousand proteins measured during the experiment and discover its functional impact. We pursue a personalized view to disentangle the specifics of protein abundance in each of the six participating individuals. We demonstrate that machine learning using self organizing maps (SOM) in combination with different analysis tools enable a personalized and intuitive view on the data. Application and adaptation of SOM machine learning to time-resolved protein abundance data is novel and challenging due to the special data type, unknown error structure and possible methodical biases of the data.

In the first part of this publication we therefore address methodical issues related to the proteomics data set. In the second part we focus on the functional interpretational to answer question such as how urine protein abundance is affected by decreased salt consumption and isolation in the space flight simulation chamber and what biological processes were involved at different stages of the experiment.

## Results

### SOM abundance portraits and sample trajectories

**Figure 1:**
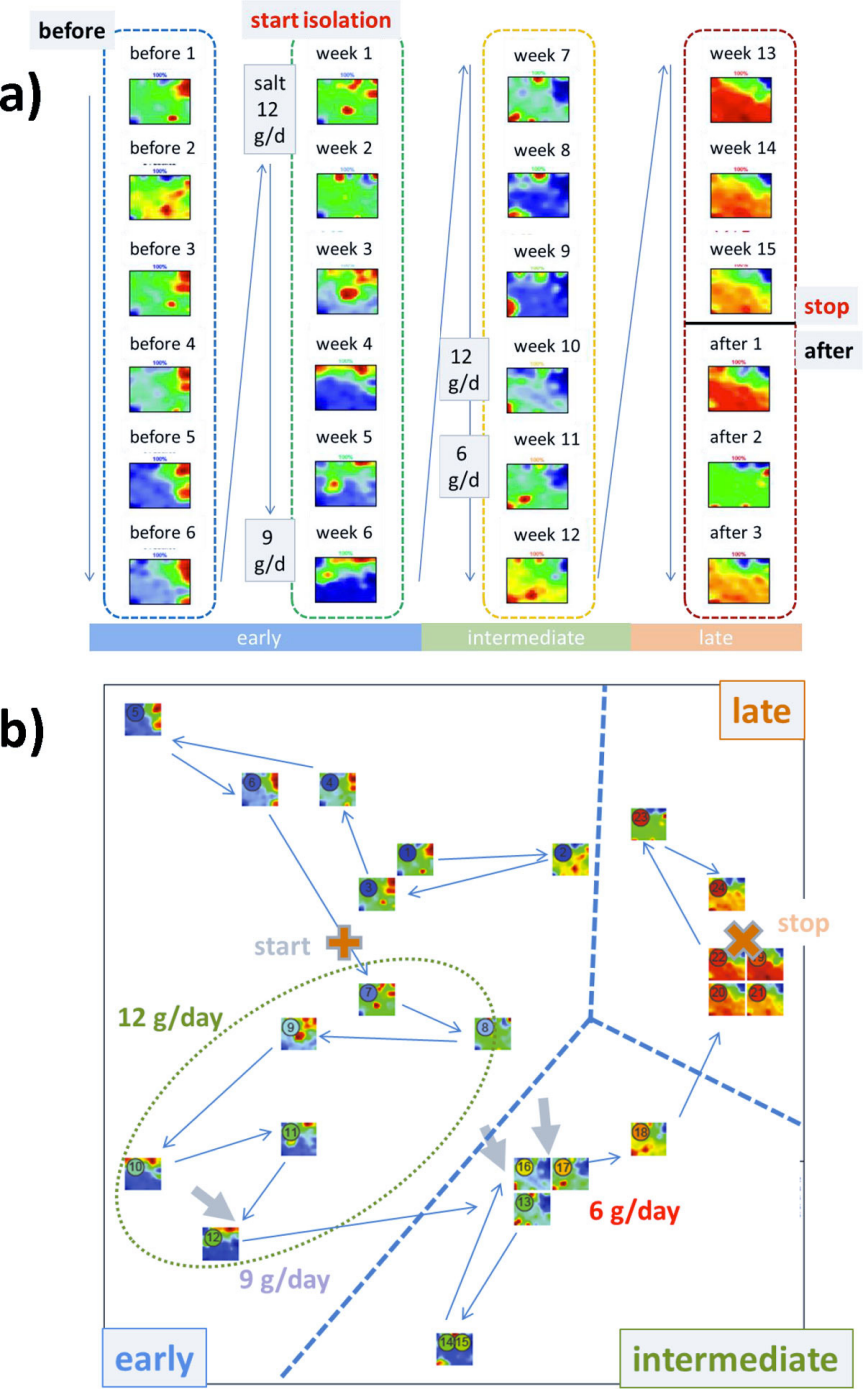
**Time dependent SOM proteomics portraits of urine samples taken before, during and after the isolation experiment (panel a) and time trajectory as obtained using 2^nd ^level SOM mapping (panel b)**. The results refer to the 'mean-volunteer analysis' by averaging the proteomics data over the six volunteers at each time point. Each column of images in panel a refers to one cluster as determined using 2^nd ^level SOM similarity analysis shown in panel b. Thin arrows indicate the temporal order of the specimen and thus the trajectory of the urine proteomics samples. The amount a salt consumption per volunteer and day during the isolation experiment is indicated in the figures: the arrows indicate the times of changing salt consumption. The dashed lines divide the trajectory into early, intermediate and late time ranges. The 'early' time-regime further subdivides into two clusters of samples collected before and after start of the isolation experiment (ellipses). Alternative independent component analysis of the sample trajectory is given in the supplementary text (Additional file 1).

Figure 1a shows the gallery of protein abundance landscapes as seen by the SOM-portraits. They visualize the mean protein abundances averaged over the individual volunteer data at each time point of sample collection. Hence, each landscape 'portrays' the proteomics phenotype of the about 2,000 protein species identified by mass spectrometry in the urine samples (IPI items). Proteins with high topmost over and under-expression levels are localized in the red and blue spot-like regions, respectively. The spot patterns clearly change in the course of the experiment reflecting alterations in the proteomics phenotypes potentially caused by isolation, modifications of salt (NaCl) consumption and presumably other factors.

Panel b of Figure 1 shows the so-called 2^nd^-level SOM which visualizes the mutual similarities between the samples in a two-dimensional plot. The samples pass virtually four time windows where the first and second ones were indicated by dotted ellipses: The first window includes the samples taken before starting the isolation experiment. The second time window lasts roughly until the end of the sixth week of isolation in which salt consumption is reduced from 12 g/day to 9 g/day. The third period ends after week no. 11, i.e. two weeks after salt consumption is further reduced to 6 g/day. The last time window finally includes the samples taken in the last three weeks of the isolation experiment and the three sample points taken afterwards. Note that the transition between time window two and three forms a sort of turning point of the trajectory after that the proteomic landscapes in the phase space of the 2^nd ^level SOM 'move' back in direction towards the starting point. According to the amount of salt consumption the samples taken before/after this turning point refer to higher and lower salt consumption, respectively. In a more rough view we divide the data into an 'early', 'intermediate' and a 'late' time regime as indicated in Figure 1: It considers the similarity of the abundance landscapes in the first two time windows and aggregates them into one early phase.

In the supplementary text we analyzed similarity relations using independent component analysis (ICA) projecting the samples in linear scale. ICA virtually confirms the results obtained using 2^nd ^level SOM.

### Spot trajectories and module selection

**Figure 2:**
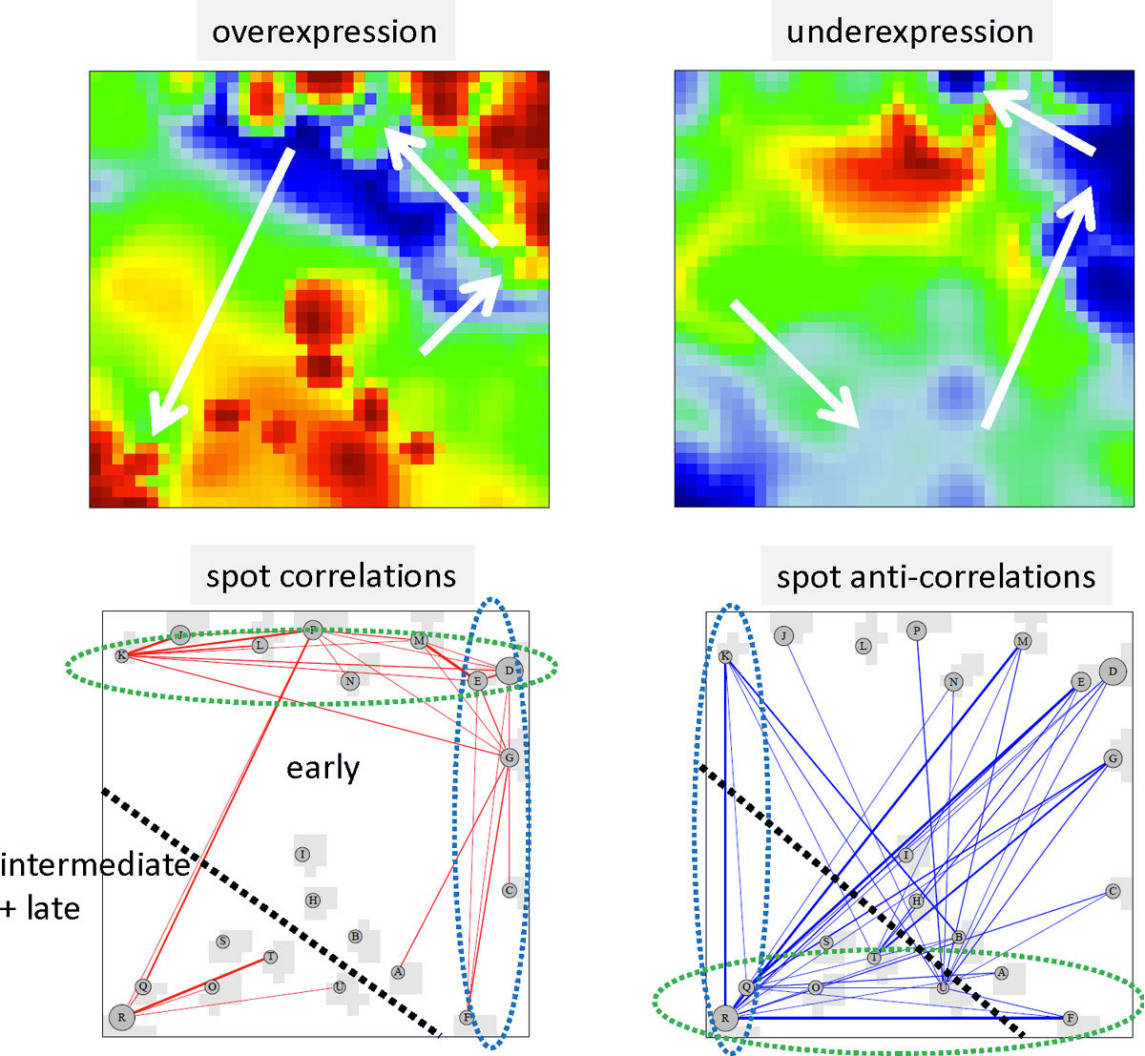
**Spot trajectories (part above) and mutual spot correlations: The over- and under-expression spot summary maps collect the red and blue spots observed in the individual portraits into one master map, respectively**. The arrows roughly illustrate the time-trajectories of over- and under-expression spots before, during and after the isolation experiment (see also the individual SOM portraits shown in Figure 1a). The correlation and ant-correlation maps visualize mutual correlations between the spots in terms of the weighted topological overlap (wto) measures for positive and negative correlations, respectively. Spots are connected by lines for strong correlations/anti-correlations.

The SOM-algorithm distributes the proteins over the map such that co-expressed proteins become located nearby. In consequence, proteins specifically up-regulated in one of the time regimes aggregate into red spot-like textures at a certain position of the map. With evolving time of the experiment the spot patterns change and, in particular, existing spots disappear and new ones appear at new positions (see Figure 1a). Figure 2 (upper part) illustrates these spot trajectories for red over- (left panel) and blue under- (right panel) expression spots. The so-called summary maps aggregate all red or blue spots observed in the individual profiles into one master map, respectively. The arrows illustrate the temporal order of appearance of the respective spots: Due to the self-organizing properties of the map red and blue spots 'rotate' in counterclockwise direction along the edges of the map in a central-symmetrical fashion. I.e., as a rule of thumb red and blue spots often appear as antagonistic twins indicating that each state is characterized by a set of up-, and a set of down-regulated proteins as well.

This property of self-organization is reflected in the spot-spot correlation and anti-correlation maps which were calculated using a weighted-topology overlap network approach as described in the Methods section and in ref. [12]: The bottom left panel in Figure 2 shows that spots up-regulated in the early time range are mutually highly correlated forming a sort of continuum of states located in right-upper part of the map. The two time windows in the early range are consequently associated with spots along the right and upper border of the map, respectively. The intermediate and late time ranges are accompanied by a marked shift of the spot position towards the lower left corner of the map thus allowing to associate the proteins within the respective spots with the discontinuous changes in samples trajectory described above (see also Figure 1). The anti-correlation map (bottom right panel in Figure 2) supports the view that spots up-regulated in the early and intermediate/late time ranges are down regulated at intermediate/late and early time ranges, respectively. Hence, the characteristic breakpoints along the spot trajectories observed can be associated with discontinuous changes of protein abundance detected in the spot trajectories.

In the next step we address the question how to select the spots appropriately or, in other words, how to segment the map properly into regions of co-regulated proteins. Besides the over- and under-expression spot selection algorithm we also applied alternative methods based on correlation and K-means clustering. Details and results of this analysis were provided in the supplementary text.

We found that the spot selection method is not crucial for extracting the basal dynamic properties of the system. In dependence on partial needs, e.g. to extract strongly differentially expressed proteins or larger groups of mutually co-expressed or even largely invariant features we recommend the overexpression, correlation or K-means clustering method, respectively. Here we will focus on the overexpression spot selection method because it is a good choice for marker selection which includes up- and down-regulated features as well. Selected results for the correlation and K-means clustering methods are presented in the supplementary text (Additional file 1).

### Spot profiles and functional analysis

**Figure 3:**
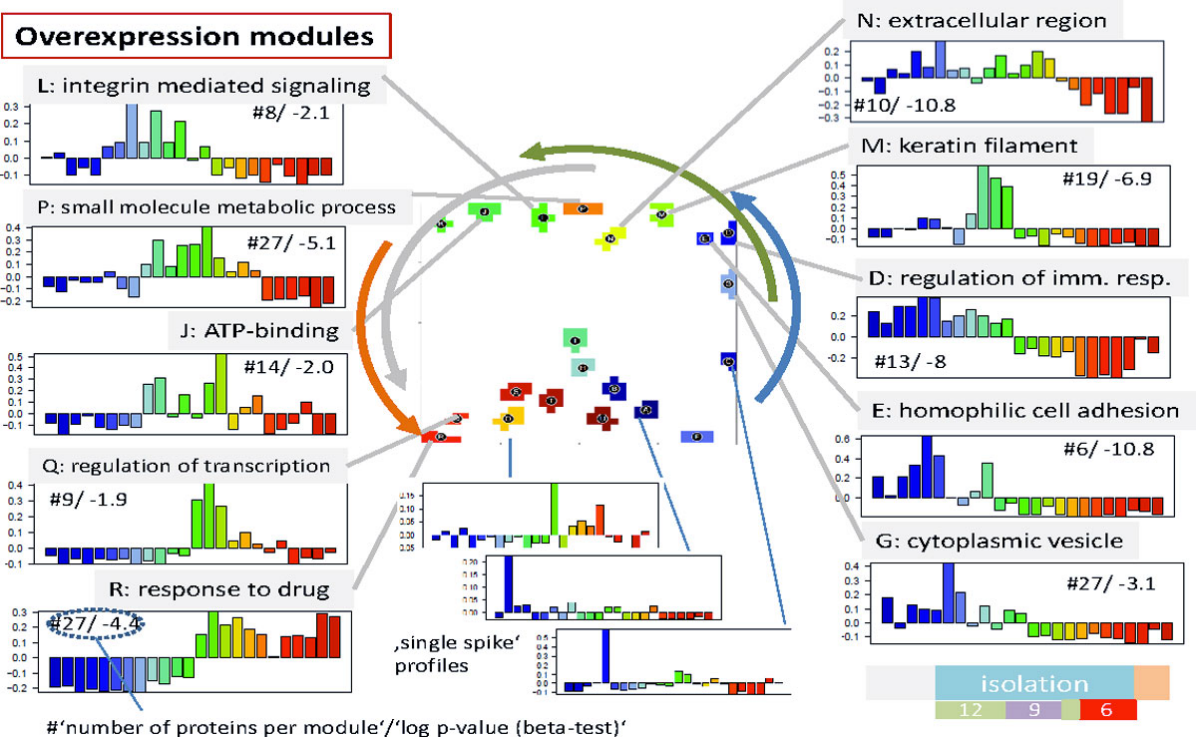
**Protein abundance profiles are assigned to the overexpression spot modules together with the leading functional context as obtained using gene set enrichment analysis**. Spots are assigned by capital letters. So-called single spiked profiles accumulate in rare spots in the lower-right part of the map. Lists of leading proteins are given for each spot in the supplementary text (Additional file 2). The vertical axis of the profiles is scaled in units of differential abundance, ΔE, representing the mean binary detection level centralized with respect to the mean abundance in all samples at all times.

Figure 3 assigns the spot profiles to selected overexpression spots. These profiles are mean time-dependent protein abundance data averaged over all meta-features included in the respective spot. The meta-features, in turn, are mean protein abundance data averaged over all single protein data contained in each meta-feature. Hence, the spot profiles are mean profiles characterizing the average abundance of the single proteins included in the respective spot. Most of the profiles show a wave-like shape with a maximum and minimum in different time windows reflecting the dynamic up- and down-regulation of proteins during the experiment. In direction of the spot trajectories discussed above, the abundance maximum seen in the individual spot profiles shifts to later times. The spot trajectory thus reflects first of all the phase-shift φ of the wave-like profiles which roughly increases from φ ~ 0-T*/2 for early activation (e.g. spot D) to φ ~ T*/2 - T* for activation at intermediate and late times (e.g. spot R). Here T* denotes the period of the changes, e.g. given as total time of the experiment. The spot profiles differ however not only in the position of their abundance maximum but also in the time delay between maximum and minimum abundance and also in their shape which can resemble more a harmonic cosine (e.g. spots G and R) or more a single peaked function (e.g. spots M and Q). The period can cover the whole duration of the experiment, i.e. T*~105 days (e.g. spots D and J) or a considerably longer or shorter time, T ~ 2 T* (e.g. spots E and R) or T<T* (e.g. spots L and P), respectively. Note that periodic changes of protein abundance can be induced by different extrinsic factors such as the activity, nutrition and working regime (e.g. night shift work during the experiment) of the volunteers, salt consumption but also intrinsic ones such as hormone activities (e.g. of andosterone, see discussion) and thus the period, or in other words, the degree of recovery of protein abundance after its perturbation, can deviate from the time span of the experiment.

Hence, the spots along the spot trajectory represent clusters of proteins concertedly activated and deactivated in sequential order during the experiment and differing also in the time of activation and the degree of recovery of the initial state at the end of the experiment. The overexpression spots contain from 6 to 27 proteins (as given in Figure 3) whereas the correlation and K-means spot clusters are markedly larger with 23-76 and 39-93 proteins per cluster, respectively (see respectively (see supplementary text; Additional file 2 and 3). Despite their differing size, the respective spot profiles taken from comparable regions of the map look very similar (compare Figure 3 with the respective figures shown in the supplementary text for correlation and K-means clustering).

The different time profiles of the spots allow us to relate them to different properties of the sample trajectory depicted in Figure 1. Particularly, spots showing different levels of protein abundance at the start and the end of the experiment (i.e. with periods T ≠ T*) are responsible for the shift between the start and end points of the sample trajectories whereas spots with cosine-like profiles and T~T* and also spots with peak-shaped profiles are mainly responsible for the turning point of the trajectories because the respective proteins mostly recover their abundance state during the experiment (see Figure 1).

**Figure 4:**
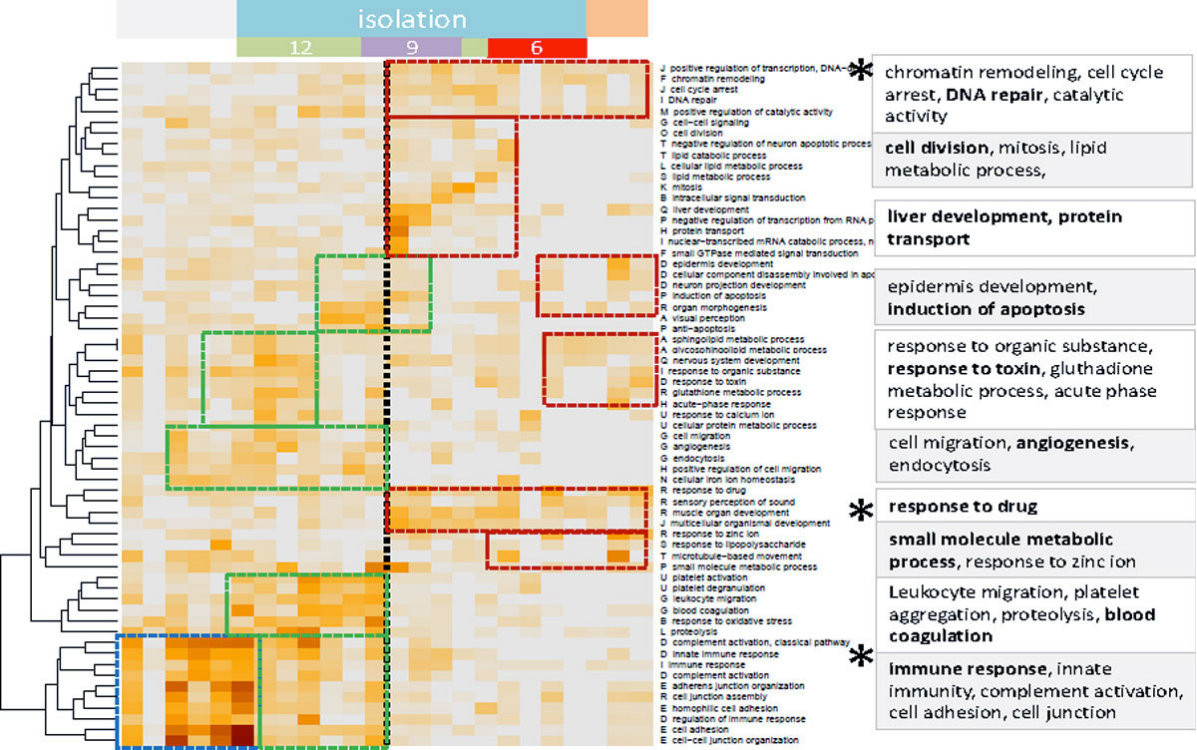
**Global enrichment analysis heat map: The map clusters top GO-sets of the category 'biological process' enriched in overexpression spots of the time series**. The dashed rectangles indicate time regions of marked enrichment of the processes listed in the right part of the figure (orange to brown colors refer to increasing enrichment). The vertical dotted line divides early from late time regimes. Processes marked with an asterisk do not fully recover during the experiment. They are related to the shift between the start and end points of the sample trajectories (see Figure 1)

Enrichment analysis using more than 2000 predefined groups of proteins referring to different GO-terms from the categories 'biological process', 'cellular component' and 'molecular function' allowed us to assign the functional context to each of the spot clusters selected. In Figure 3 the leading gene set is given for each overexpression spot cluster. The results of a more detailed analysis are given in the heat map shown in Figure 4 (see refs. [13,14] for the description of the method) and in the supplementary text where we map and profile selected protein sets in detail. According to these analyses the early time range is characterized by the activation of inflammatory processes and angiogenesis (gene sets inflammation, extracellular region, cell adhesion, complement activation, proteolysis, angiogenesis and Calcium ion binding) whereas intermediate and late responses are related to developmental and regenerative processes (development, mitosis, regulation of transcription, chromatin remodeling) and stress and drug response (small molecule regulative process, response to oxidative stress, hypoxia, apoptosis, response to Zinc, Magnesium ion binding, G-protein coupled activity), respectively. Note that part of the processes related to inflammation, drug response and also to genome and transcriptome activity (chromatin remodeling, DNA repair) can be attributed to the lack of recovery of the sample trajectories (these processes are marked by the asterisks in Figure 4).

Clusters of proteins associated to the response of the organism to 'NaCl' deficiency are identified previously using a comprehensive interactome network analysis [10]. We mapped proteins from these clusters into SOM space and found that they mostly refer to the early, and to a less degree, to the intermediate-time response (see supplementary text).

**Figure 5:**
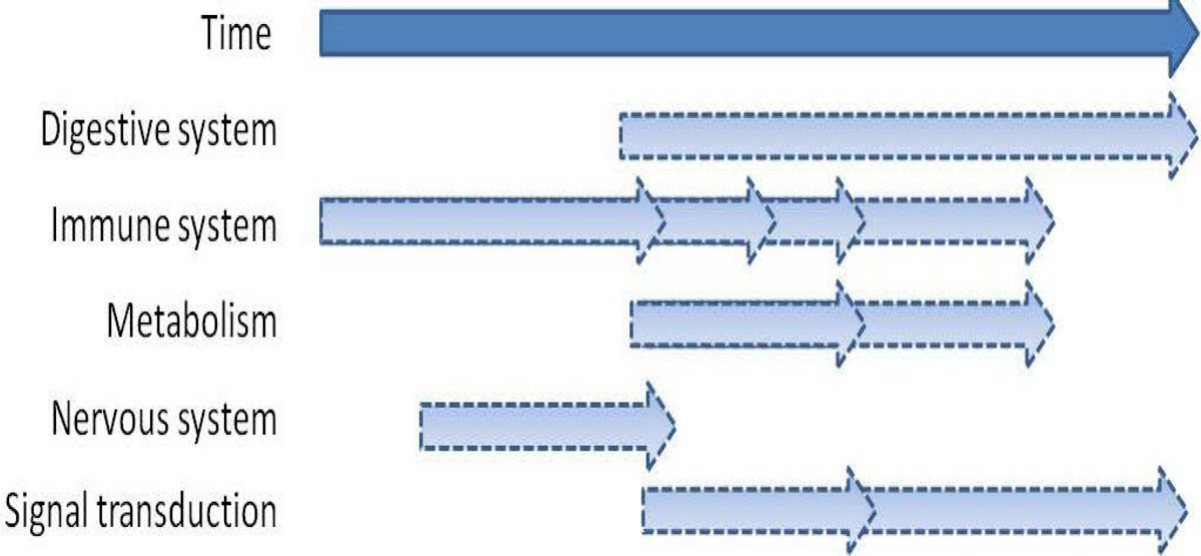
**Summary of results obtained from pathway flow analysis: The light blue arrows indicate characteristic processes activated with progressing time of the experiment**. The processes are identified by comparing the PSF of KEGG-pathways with the spot profiles of protein abundance (see supplementary text for details).

Pathway signal flow analysis (PSF) represents an independent option to discover the functional context of the spot profiles. In contrast to gene set enrichment analysis it takes into account the network topology of selected pathways taken from the KEGG database to obtain PSF-profiles which are compared with the abundance profiles of the spots. It turned out that early and intermediate protein abundance changes are associated with inflammatory responses and metabolic processes (fatty acids, nucleic acids and amino acids) indicating alterations of nutrition and partly starvation followed by activation of regenerative processes (Wnt-pathway, N-glycan biosynthesis) in the intermediate time range and of stress response signaling (p53 and mTOR-signaling pathways) and digestion at late times of the experiment (see Figure 5 and supplementary text for details). Many pathways lead to the activation of protein kinase C and inositol-triphosphate signaling cascades in agreement with the enriched protein sets related to signal transduction such as Ca^2+ ^binding and G-protein coupled receptor activity.

### Individual volunteer analysis

**Figure 6:**
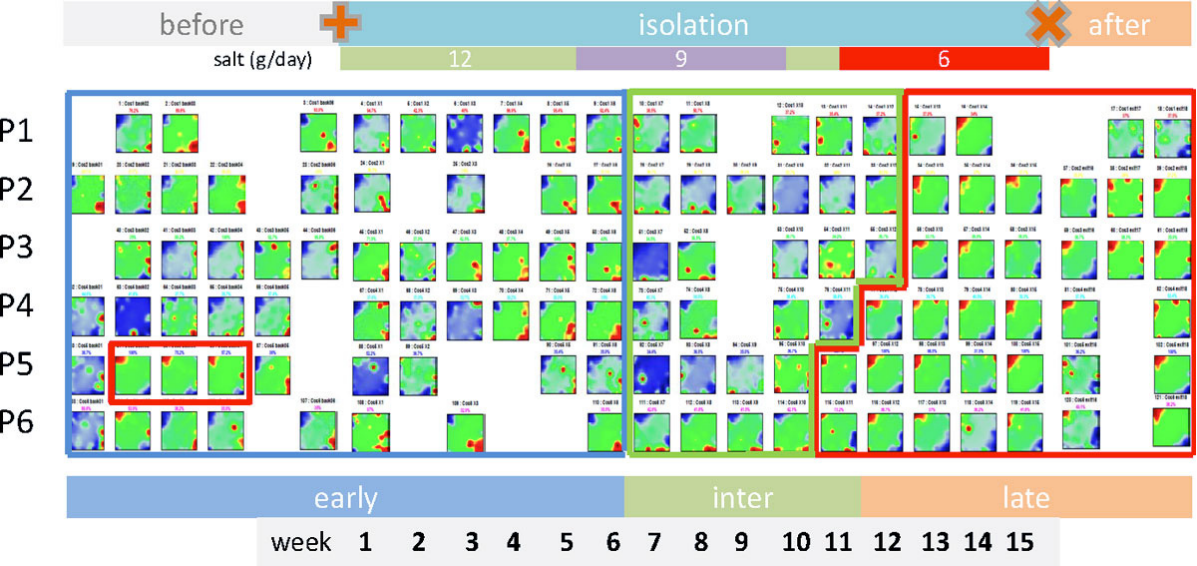
**Gallery of SOM portraits of the individual volunteers: Each row refers to one proband (P1 to P6) and each column to one time point of sample collection**. Empty positions in the matrix refer to missing data because no samples were collected. The blue, green and red frames include samples showing the characteristics of early, intermediate and late responses, respectively. Note that the SOM textures cannot be directly compared to the textures of the mean volunteer analysis (shown in Figure 1) because both sets of maps are trained independently. Note that three out of five urine samples taken from proband no. 5 (P5) and possibly one taken from P6 before starting isolation express proteomics characteristics observed apart from that in the samples of all volunteers in the late time range only (see the red frames for P5).

So far we presented results based on the averaging of the abundance of each protein at each time point over all six volunteers. This 'mean volunteer' analysis allowed extracting mean effects induced by isolation and varying salt consumption but it neglects individual differences between the volunteers. We therefore performed a second independent SOM analysis of the individual data of each volunteer. Figure 6 shows the gallery of time-dependent 'personalized' portraits of all six probands (P1 - P6). As for the 'mean volunteer analysis', the protein abundance landscapes can be divided into typical color textures assigned to the early-, intermediate- and late-response types, respectively. Simple visual inspection of the portraits shows that the abundance patterns of most of the volunteers alter in parallel (see the colored frames in Figure 6). Partly, one observes however small variations in the time-dependent changes: For example, the portraits of P4-P5 switch into the time regime of the 'late' type almost one-two weeks earlier than that of P1-P3. Late-type protein abundance patterns were observed for P5 in three samples taken before starting isolation.

**Figure 7:**
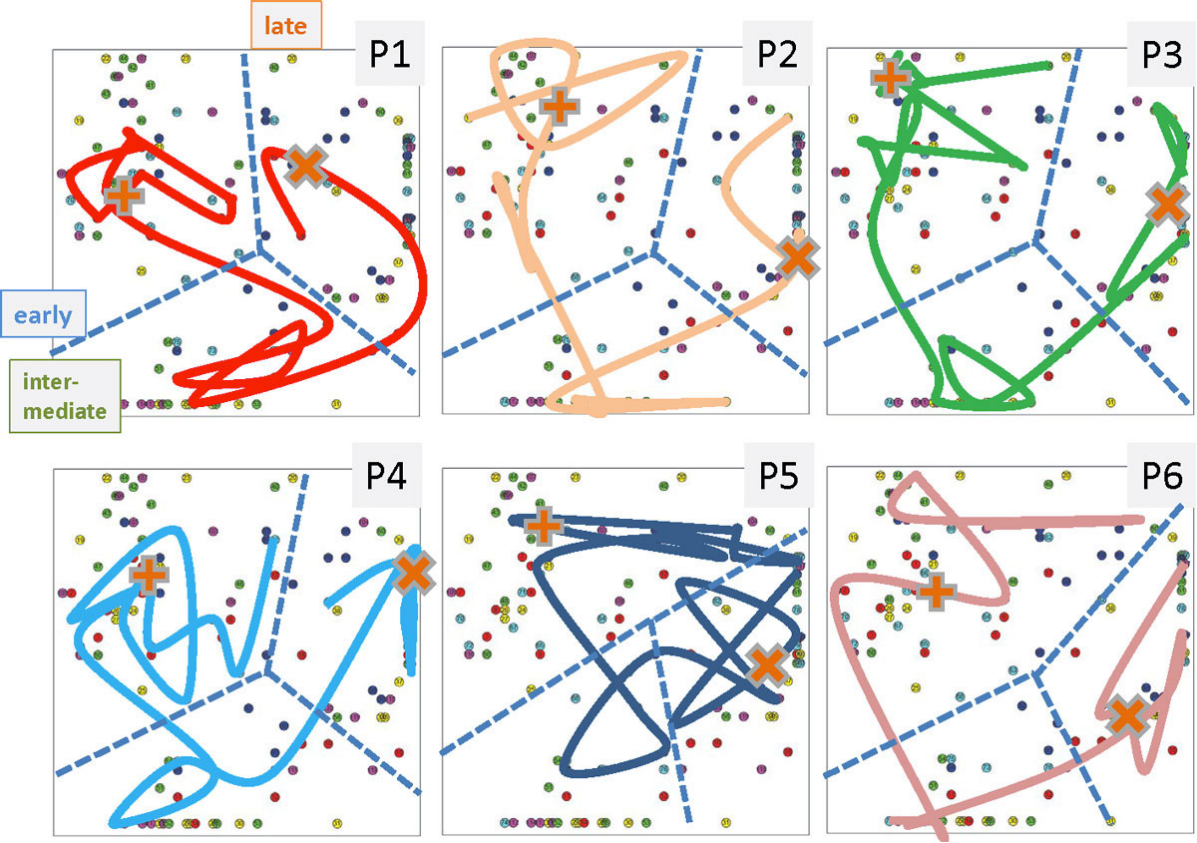
**Individual sample trajectories of each of the six probands (P1 - P6) in 2^nd ^level SOM coordinates**. Each of the trajectories can be divided into early, intermediate and late time regimes despite small individual differences (dashed lines). The 2^nd ^level SOM was trained with the data of all volunteers. The trajectories shown connect the time-dependent samples of the respective proband. The symbols '+' and 'x' indicate the start and the end of isolation. Note that the trajectory of proband no. 5 (P5) enters the 'late time range' still before starting isolation in correspondence with the respective proteomics portraits shown in Figure 6.

Figure 7 shows the individual sample trajectories of each of the volunteers using 2^nd ^level SOM analysis. One sees that virtually each trajectory can be clearly divided into the early, intermediate and late time ranges. The borderlines separating the different time regimes however slightly shift between the individuals. One also sees that volunteer P5 is characterized by a certainly more intricate trajectory reflecting his individual specifics.

Next, we performed functional analysis by applying gene set enrichment clustering to the single volunteer data (see supplementary text for details). In general, the functional context of the different time ranges agrees with that of the mean volunteer analysis. However, the larger set of individual sample data provides a more detailed view on the specifics of each volunteer. For example, features related to 'immune response' were either up-regulated in the early phase of the experiment only (P1, P4, P6) or, in addition, again in the late phase (P2, P3, P5).

### Organ related protein abundance

**Figure 8:**
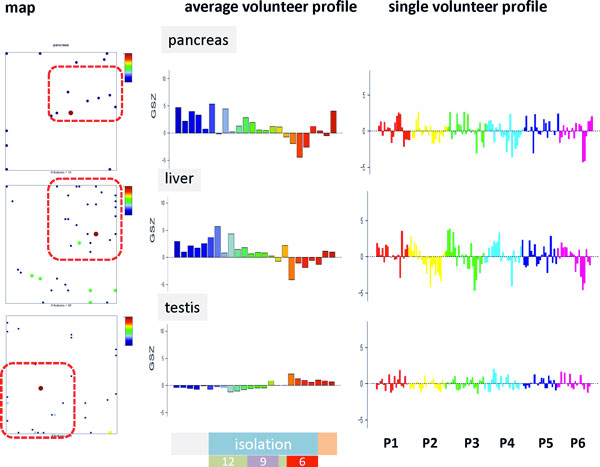
**Tissue specific protein abundance: Tissue specific protein sets are taken from TiGR **[15]**and mapped into the single volunteer map (left part)**. The red rectangles illustrate regions of increased local density of the respective proteins. These regions refer to the early_up, and late_up time ranges. The set-profiles shown in the middle part clearly reveal the different time profiles in the average volunteer analysis. The respective single volunteer analysis reflects proband-specific differences between their tissue abundances. The respective data of additional tissues (kidney, muscle, stomach, skin, lymph node, blood, prostate, brain, colon) is given in the supplementary text.

Proteins not of renal origin fall in urine from blood and in blood from the respective tissues and cells. We used Tissue specific Gene Expression and Regulation data base (TiGER, [15]) to assign protein species to different tissues and assess their abundance in the urine samples studied (see Figure 8 and supplementary text). First we map the tissue-related protein sets to SOM space: It turned out that the respective species of a series of tissue sets accumulate in different regions of the map which were assigned to different time ranges. For example, pancreas and liver proteins show an increased local density in the area of early_up proteins, muscle proteins in the region of intermediate_up region and testis proteins in the late_up region. The respective time profiles confirm the expected activation patterns. We found that proteins from liver, pancreas and kidney show increased abundance before and at the beginning of the isolation experiment. Proteins from muscle are overexpressed at intermediate times of isolation and proteins related to testis and stomach at the end and after isolation. Protein sets related to skin, lymph nodes, blood, prostate, brain and colon show virtually no or only a very weak time dependence in the single volunteer analysis.

The single volunteer tissue profiles again reveal individual differences between the probands: For example, liver proteins of P1 and P5 respond much weaker than liver proteins of the other volunteers. The individual profiles of prostate proteins clearly show time dependencies which however are averaged out in the average volunteer profile due to their asynchronous character (see supplementary text provided in Additional file 1).

### Total protein abundance analysis

**Figure 9:**
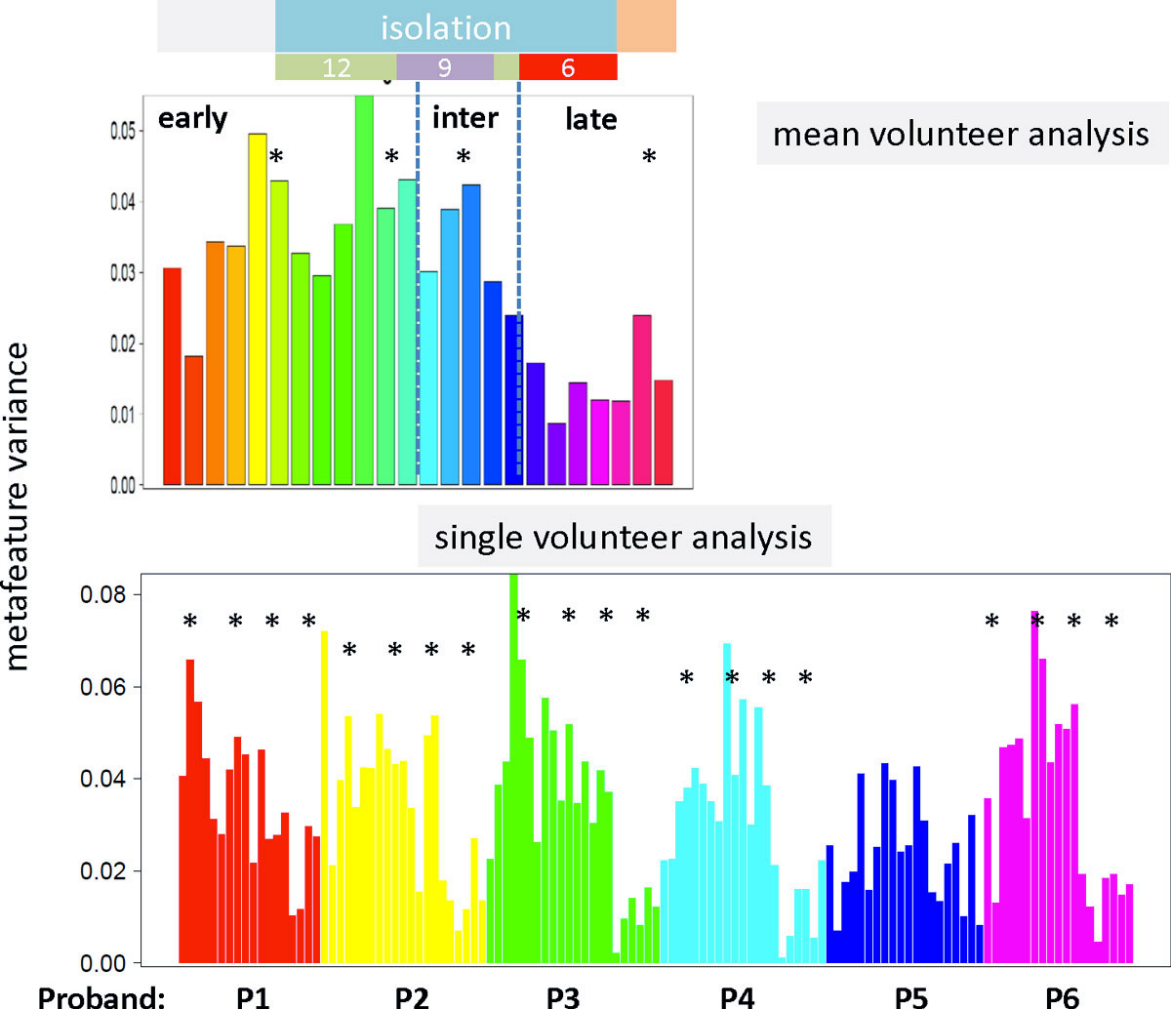
**Total level of protein abundance averaged over all probands (part above) and separately for each proband (part below) as a function of time: We calculated the variance of the meta-feature abundance landscapes obtained after SOM training using not-centralized protein abundance profiles**. The oordinate values thus estimate the mean squared amplitude of overall abundance as a function of time. The asterisks indicate the sequence of four peaks observed virtually in all data sets.

In addition to single-, meta-feature and spot related abundance levels using centralized values (i.e. normalized ones with respect to the mean value averaged over all volunteers and time points) we analyzed the time profile of the total protein (i.e. integral) abundance level in terms of the variance of the respective meta-feature abundance landscapes (Figure 9). The abundance landscapes refer to a separate SOM training described below and in the supplementary text. It turned out that, on average, the total abundance level slightly increases before isolation in the early time range but then, after a plateau, it steeply decreases in the intermediate and late time ranges until the end of isolation of the volunteers. Hence, isolation causes the overall decrease of protein abundance in the urine samples. In other words, processes down-regulated in the intermediate and late time regimes obviously involve a larger number of proteins and/or their stronger abundance changes than processes up-regulated in the late time regime. Analysis of the population map supports this expectation (see supplementary text): About 27% of the proteins and 33% of the meta-features are up-regulated in the early time range whereas only 20%/13% of the proteins/meta-features up-regulate in the late regime. The remaining 53%/54% refer to rare and single spiked features. Inspection of the individual volunteer data again reveals slight differences between the total abundance levels of the probands and between details of their respective time courses (Figure 9). For example, P5 shows a decreased total level of protein abundance.

The detailed inspection of all total profiles indicates a certain fine structure in terms of three to four local peaks which appear immediately before or at starting isolation, after reducing salt consumption from 12 to 9 g/day and further to 6 g/day and at the end of the experiment (see the asterisks in Figure 9). Interestingly, adjacent local peaks of total protein abundance are separated by about five weeks possibly reflecting an intrinsic infradian rhythm in protein abundance. The total abundance level slightly increases after finishing isolation indicating slow regeneration of the volunteers. Part of this fine structure is found also in the abundance profiles of selected spot modules, e.g. of the overexpression spots G, E, M, P, J and Q (see Figure 3) expressing one or two sharp peaks in the time regions identified in the total abundance analysis.

To get deeper insight into this phenomenon we performed a full and detailed SOM analysis of the absolute abundance profiles using a similar approach as developed for differential abundance data. Recall that analysis using centralized profiles applied so far focuses on abundance changes independent of the abundance level. For example, virtually invariant profiles of high and of low abundance levels were clustered together in this case. Absolute abundance values certainly distinguish between these two situations. Thus the analysis of absolute abundance profiles is expected to provide additional information about the abundance levels of the proteins in the course of the experiment. Detailed results were described in the supplementary text (Additional file 1). We found that a series of processes become activated in relatively narrow time windows of peaked abundance at the four fixed times identified in total abundance analysis, namely at or immediately before isolation (angiogenesis, complement activation and others), at or immediately after reducing salt consumption to 9 g/day (focal adhesion and cytoskeleton) and to 6 g/day (cell differentiation and organ development) and near the end of the experiment after isolation. The latter trend suggests recovery of the initial state before starting isolation. Double peaked profiles combine peaks at late and intermediate times (e.g. metabolic process and apoptosis). Importantly, immune response processes are permanently active during the experiment with a slight decay in the late time range. About 60% of the proteins are permanently expressed on low abundance levels during the experiment whereas about 7% - 10% are permanently expressed on high abundance levels. This result agrees with our estimation using centralized data.

## Discussion

### SOM portrays urine proteome abundance landscapes with high temporal and individual resolution

**Figure 10:**
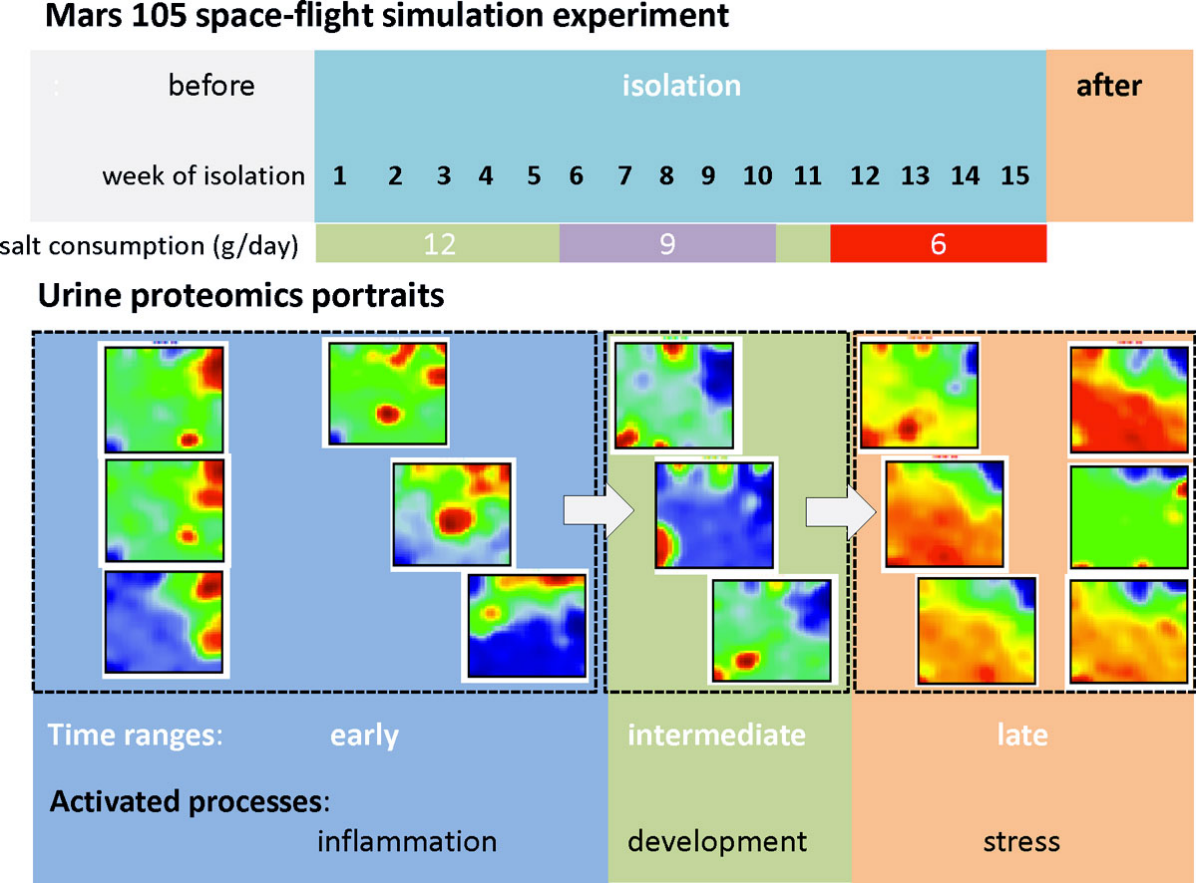
**Summary graphics of the time course of urine proteomics during the Mars 105 space-flight simulation experiment**.

From a methodical point of view we aimed at analyzing a complex high-content data set of about 2000 protein species measured at 24 different time points for six individuals in terms of clustering and class discovery, feature selection an functional information mining using SOM machine learning. The data set is unique and exceedingly valuable with respect to its scope, duration, and level of environmental control. It has been shown that the analysis pipeline chosen is well suited to extract longitudinal (i.e. time dependent) as well as transversal (i.e. volunteer specific) information in detail. One special strength of the approach can be seen in its visualization capabilities allowing the intuitive perception of essential properties of the data such as the detection of spot-like clusters of differentially and co-expressed proteins, and especially, of their time-dependent changes and/or their volunteer-specific variations. The basal results of our SOM analysis are summarized in Figure 10 and Table 1.

**Table 1:** Overview over effects observed in space-flight isolation experiments after analysis of urine proteomics.

Time range	early	intermediate	late
**week of isolation**	before isolation and week 1-6	week 7 - 11	week 12 - 15 and after isolation
**NaCl consumption**	12 g/day (week 1-6)	9 (week 7-9), 12 (week 10) and 6 g/day (week 11)	6 g/day (week 12-15)
**Activated biological processes (enrichment analysis)**	inflammation, cell adhesion, blood coagulation, proteolysis, angiogenesis, Ca^2+ ^binding, extracellular region	cell division, lipid metabolism, skin development, keratinization, chromatin remodeling, response to oxidative stress and hypoxia, regulation of apoptosis	response to drug/toxin, small molecule metabolic process, intracellular, Mg^2+ ^binding, response to Zinc, Cell death, G-protein coupled receptor, regulation of blood pressure (renin/angiotensin)
**Activated pathways (PSF analysis)**	immune response; nervous system; nucleotide, amino acid and lipid (butanoate) metabolism	digestive system; metabolism; regenerative processes (Wnt-signaling pathway and N-glycan biosynthesis)	signal transduction; response to stress (p53-, mTOR-signaling pathway), energy metabolism (ubiquinone biosynthesis)
**Activated tissue responses**	Liver, kidney, pancreas, (partly skin)	muscle	testis, stomach, (partly liver and kidney)
**Relation to previous results**	NaCl related interactome activated [10], NaCl storage in an osmotically inactive form and micro-vascularization [6,7], renal proteins activated [11]		blood pressure decrease and aldosterone level increase [4]
**Total protein abundance**	increasing and high	decreasing	low
**Percentage of proteins up-regulated**	27%	20%	
**Percentage of invariant, noisy and single spiked proteins**	>50%		
**Up-regulated modules**	G, E, D, M, N, L	J, P, Q	R
**Down-regulated modules**	R, Q, J, P	D, E, G, Q, R	G, E, D, M, N, L, P
**Module-related proteins (differential abundance)**	see Additional file 2		
**Module-related proteins (absolute abundance)**	see Additional file 3		

We found that

- The dynamics of urine proteomics can be described in terms of sample trajectories reflecting similarity relations between the protein abundance landscapes of the samples as a function of time; or alternatively, in terms of spot trajectories reflecting similarity relations between the time profiles of different groups of co-expressed proteins. Both types of trajectories describe the dynamics of urine proteomics in a complementary fashion.

- The time course of urine proteomics splits roughly into three time ranges, an early, an intermediate and a late one using data averaged over all six volunteers studied. Each of the time ranges is characterized by relatively similar protein abundance landscapes and thus by similar biological processes activated (and deactivated).

- The abundance of about one half (47%) of the 2000 protein species clearly changes in the course of the experiment. The total protein abundance level is maximum in the early time region and then it progressively decreases until the end of the experiment.

- The remaining other half of all proteins (53%) is either expressed invariantly virtually not or weakly responding to the experiment or it shows so-called rare, noisy and single-spiked profiles. The respective protein species are expressed only at very few time points for a small part of the volunteers only. The further analysis and interpretation of these profiles is beyond the scope of this study.

- The volunteer averaged sample trajectory passes through a turning point at the end of the early time range and then it moves backwards in direction of the starting point revealing the partial recovery of the protein abundance state observed before starting isolation on one hand, but also certain differences between the start and end points of the experiment on the other hand.

- The three characteristic time ranges are consistently observed in the individual time course proteomics of all six volunteers. Small but clear individual differences are observed (e.g. relatively low abundance levels of proband no. 5 and slight variations of the start and end points of the time ranges between the individuals). Here we focus on the ubiquitous effects. We note however, that our method enables the personalized view on these individual differences.

- The similar time courses of urine proteomics of all volunteers let us conclude that the three time ranges reflect representative and essential physiological regimes associated with isolation, salt consumption and presumably also other factors. Note that the intermediate and late time ranges start one week after reducing the daily salt consumption from 12 to 9 g and further to 6 g, respectively.

- The so-called 'spots' collect co-expressed proteins representing regulatory modes associated with distinct biological processes which can be identified using previous knowledge by applying enrichment or pathway flow analysis. In total we identified about ten different modes of protein abundance. Application of different methods of spot selection (e.g. using overexpression, correlation or K-means clustering techniques) essentially provides a consistent picture however with different numbers of proteins associated with the different modules. Significance of co-expression of the proteins of each module was estimated using a beta test adapted to the spot clusters identified in the SOM analysis.

- The larger number of spot modules exceeding the number of time ranges specified reflects the fact that this rough classification into three ranges further splits into different dynamic modes characterized by their phase shift, period and particular shape.

- The separate SOM analysis of absolute abundance values provides additional and complementary results: It allows to identify permanently present and weakly expressed proteins, respectively and it allows to extract single and double peaked abundance profiles presumably indicating immediate responses of urine proteomics to changes of salt consumption and/or infradian rhythms due to other factors.

### Urine proteome abundance reflects variations of sodium balance and of related molecular processes

The similar time courses of urine proteomics of all volunteers let us conclude that the three time ranges reflect representative and essential physiological regimes associated with the duration of isolation and salt consumption, the only dietary factor that systematically and markedly changed in the course of the experiment. The intermediate and late time ranges start not later than one week after reducing the daily salt consumption from 12 to 9 g and further to 6 g, respectively (recall that samples were collected only once a week which limits the time resolution of the experiment). Note that a salt consumption of 12 g/day to 6 g/day is considered as the normal range of human daily salt intake. Hence the observed effects are not related to excessive or deficient salt intake compared with this normal range but rather reflect subtle responses to slight but systematic alterations of salt consumption within the normal physiological limits.

For functional interpretation we applied enrichment and pathway signal flow analysis. In general, early protein activation can be related to pro-inflammatory processes as indicated by the GO sets immune response and inflammatory processes, activation at intermediate times to developmental and proliferative processes and late activations to stress and responses to chemicals. It has been reported previously that macrophages, a type of cells in the immune system, besides defending the body against infections appear also to be involved in the regulation of the salt balance and blood pressure [4,6,7]. In body regions with high salt concentrations, they cause the formation of new blood and lymph vessels especially in skin, thus helping to regulate the body's microcirculation with consequences for the blood pressure. In support of this mechanism we find that processes like angiogenesis, cell adhesion, proteolysis and proteins in cellular components like extracellular region became activated in parallel to pro-inflammatory processes. Moreover, also a set of proteins involved into an interaction network related to organisms response to salt (NaCl) taken from [10] were activated in the early time region immediately following the adjustment of the daily NaCl-dosis to 12 g. Interestingly, the activity of the protein set 'regulation of blood pressure' increases slightly in the late phase of the experiment only (see supplementary results). This set collects a group of proteins involved in regulation of blood pressure via the 'conventional' renin/angiotensin mechanisms: Their expression stimulates the release of aldosterone which in turn reduces blood pressure. Indeed, the blood concentration of aldosterone is found to continuously increase during the isolation experiment paralleled by the continuous decrease of systolic blood pressure [4].

Part of protein abundance in the early time range can be related to kidney involved in excretion and water balance in agreement with [11]. According to the generally accepted view, sodium accumulation in the human body takes place in the extracellular space and is accompanied by an increase in the rate of fluid retention and body weight gain. In space-flight isolation experiments the relative rate of body weight gain was however lower than the relative rate of gain in the total body sodium, which suggested that sodium accumulated in an osmotically inactive form presumably in bone, skin (connective tissue) or cartilage (see [7] and references cited therein). Proteins usually expressed in skin were only weakly activated in the early time range. Note however that related processes such as keratinization were clearly up-regulated. Other organ specific abundance patterns characteristic for liver and pancreas become also activated in the early time regime. These alterations in protein abundance presumably reflect the effect of isolation, nutrition and salt consumption on digestion and homeostasis. Activation of muscle-specific proteins in the intermediate and of testis-specific proteins in the late time regime are presumably consequences of the physical activity and/or of hormone production of the volunteers during the experiment.

Activation of regenerative processes in the intermediate time range at least partly might be related to reorganization of tissues involved in salt balance and storage. With progressive time of isolation protein abundance strongly decreases. Stress related signatures became increasingly into play accompanied by signatures related to drug metabolism.

Analysis of absolute abundance values shows that part of proteins related to immune response and extracellular space are permanently expressed with a slight decay in the late time range. In contrast, proteins involved in stress response and signal transduction gain in activity in the late phase of isolation. Interestingly, the abundance of proteins related to organ morphogenesis, angiogenesis and cell differentiation seem to respond immediately to changes of salt consumption by abundance peaks of 1-3 weeks duration. The question whether these effects are affected by infradian rhythms due to other effects such as the night-shift of the working regime and/or periodic changes of hormone production and salt balance [3,4] requires further studies.

## Summary and conclusions

Ground-based space station model experiments enabled a novel, profound and extended trip to our 'inner space' to discover different aspects of human metabolism. Analysis of urine proteomics data using SOM machine learning in combination with biological function mining provided detailed insights into the physiological status of healthy cosmonaut-volunteers on protein level. Protein abundance characteristics support previous results about alternative mechanisms of salt storage paralleled by the activation of immune response in the context of their influence on micro-vascularization. Based on our results we hypothesize that reduced NaCl consumption of about 6 g/day presumably will reduce or even prevent the activation of inflammatory processes observed in the early time range of isolation. Moreover, the physiological status of the volunteers systematically and consistently changed during the 105 day experiment. Extended studies such as the 500 day isolation study (Mars 500) are required to discover long term effects. Our data also show that the turning point of the time trajectories suggest a first phase of adaptation to the conditions of isolation about two months after starting the experiment. Recovery to the 'normal' physiological status before the experiment is not observed during and directly after isolation.

## Methods and data

### Experimental setup

Six healthy men aged from 26 to 41 year participated in the ground based isolation experiment. They spent 105 days in an airtight chamber with autonomous systems of life support which is installed in the Institute of Biomedical Problems of the Russian Academy of Sciences. The isolation study was approved by several ethical boards of the Russian Federation and European Space Association authorities. Written informed consent was obtained and all studies were done as outlined in the Declaration of Helsinki.

The regime of salt consumption was reduced from 12 g/day and volunteer in week 1 - 5, to 9 g/day (week 6 - 9) and finally to 6 g/day (week 11 - 15) (see Results section for details). In week 10 volunteers consumed 12 g/day. Urine was sampled (15 ml) once a week in the morning after breakfast (middle jet collection) as described previously [10,16]. In addition, four to six samples were collected from each subject before the isolation experiment and one to three after the experiment. Urine proteomics data were obtained by High Performance Liquid Chromatography and Tandem Mass Spectrometry (HPLC-MS/MS).

### Sample Preparation for Mass Spectrometry

Urine samples (15 mL) were concentrated using Amicon UltraUltracel-15 5 k tube (Millipore, USA) at 1,000 g for 1 h at 4°C. The resultant concentrate (300 ml) was then evaporated to dryness in a centrifuge evaporator. Samples were normalized up to total protein concentration of 10 mg/mL using reduction buffer containing 0.2 M Tris-HCl, pH 8.5, 2.5 mM EDTA, 8 M urea. Urinary protein level was measured by standard method with Bradford Protein Kit (Bio-Rad) according to manufacturer recommendations. To reduce cysteine residues the solution of urinary proteins was mixed with dithiothreitol (0.1 M final concentration) and incubated at 37°C. For alkylation of reduced SH-groups, the reaction mixture was cooled and mixed with small amount of concentrated aqueous solution of iodoacetamide up to its final concentration of 0.05 M. After incubation of the reaction mixture at room temperature for 15 min in darkness, the reaction was stopped by adding molar excess of 2-mercaptoethanol (10 ml per mg of added dithiothreitol). Proteins were precipitated by addition of 10 volumes of acetone containing 0.1% (v/v) trifluoro acetic acid and overnight incubation at -20°C. After centrifugation at 12,000 g for 10 min at 4°C the sediment was re-suspended in 96% ethanol (v/v), centrifuged again at 12,000 rpm for 10 min at 4°C, and dried in the centrifuge evaporator for 1 h at 45°C. Trypsinolysis of the urinary protein fraction was performed in 200 mM NH_4_HCO_3 _buffer (protein concentration about 1 mg/mL) with modified porcine trypsin (Promega, USA) added at the ratio enzyme/protein of 1:100 (w/w). After 6 h incubation at 37°C hydrolysis was stopped with formic acid (final concentration of 3.5%). The solution was centrifuged at 12,000 g for 10 min at 4°C, and the supernatant was analyzed by HPLC-MS/MS [16].

### High Performance Liquid Chromatography and Tandem Mass Spectrometry (HPLC-MS/MS)

HPLC-MS/MS experiments were performed in triplicate on a nano-HPLC Agilent 1100 system (Agilent Tech-nologies, Santa Clara, CA, USA) in combination with a 7-Tesla LTQ-FT Ultra mass spectrometer (Thermo Electron, Bremen, Germany) equipped with a nanospray ion source (in-house system) as described in [10,11,16]. A sample volume of 1 μl was loaded by autosampler onto a homemade capillary column (75 μl id, length 12 cm, Reprosil-Pur Basic C18, 3 μm, 100 A; Dr. Maisch HPLC GmbH, Ammerbuch-Entringen, Germany) which was prepared as described in [17]. Separation was performed at a flow rate of 0.3 ml/min using 0.1% formic acid (v/v, solvent A) and acetonitrile 0.1% formic acid (v/v, solvent B). The column was pre-equilibrated with 3% (v/v) solvent B. Linear gradient from 3% to 50% (v/v) of solvent B in 90 min followed by isocratic elution (95% v/v, of solvent B) for 15 min was used for peptide separation. MS/MS data were acquired in data-dependent mode using Xcalibur (Thermo Finnigan, San Jose, CA, USA) software. The precursor ion scan MS spectra (m/z 300-1600) were acquired in FT mode with a resolution of 50000 at m/z 400. The three most intense ions were isolated and fragmented by collision-induced dissociation(CID), MS/MS spectra were measured in the linear ion trap (LTQ). In data-dependent experiments, dynamic exclusion was used with 20 s exclusion duration. In data-dependent experiments, dynamic exclusion was used with 20 s exclusion duration.

### Urine proteomics data preprocessing

Raw MS/MS data from the LTQ-FT were processed to msm-files using the software RAW2MSM (version 1.10_2007.06.14) [17]. Mascot database searching was performed using Mascot Server 2.2 software (Matrix Science, London, UK; version 2.2.06); all tandem mass spectra were searched against the human IPI protein sequence database from the European Bioinformatics Institute (version 3.82; released 06.04.2011; 92104 entries) assuming the digestion enzyme trypsin. Search criteria included two missed cleavage, carbamidomethyl of cysteine as a fixed modification, oxidation of methionine as a variable modification, fragment ion mass tolerance of 0.50 Da (10 ppm). Protein identifications were accepted if they contained at least 2 identified peptides with ion scores >24. The results were verified against reverse database to a false discovery rate of less than 1% using Scaffold 4.0 software (version Scaffold-01_07_00, Proteome Software Inc., Portland, OR). All Mascot search results and parameters are submitted to the PeptideAtlas (submission PASS00592) repository and are freely available for download with the URL: http://www.peptideatlas.org/PASS/PASS00592. The data file with peptides and proteins are also provided as Additional file 4.

This preprocessing provides 2,038 species indexed by the international protein indices (IPI) in the Mascot data base. All protein species indexed by IPI were included into our analysis. 1660 (71%) of them were explicitly assigned to genes using the biomaRt program package available in the bioconductor repository with query to Ensemble gene annotations http://www.bioconductor.org/packages/release/bioc/html/biomaRt.html. The presence/absence of each protein species in each sample was defined by binary 1/0 values providing an abundance matrix for each volunteer where each row corresponds to one protein and each column to one time point of sample selection (Additional file 5). For downstream analysis we used either these individual, volunteer-specific data (single volunteer analysis) or we calculated the mean abundance for each protein and time point by averaging protein data over the individual volunteers (mean volunteer analysis). Single volunteer abundance data are provided as Additional file 5.

The time course of abundance of each protein is called abundance or expression time profile whereas the abundance of all proteins considered at one time point is called abundance or expression state. We will use the terms 'abundance' and 'expression' (of proteins in urine) as synonyms throughout the paper. Effectively a protein species is present if its MS-signal exceeds the mean detection threshold in a constant volume of urine (15 ml). Note that the amount of proteins detected refers to a constant volume collected and thus 'protein abundance' estimates protein concentration in urine. Decreased amounts of proteins detected thus can be explained by decreased protein penetration into urine at stable water reabsorption/dilution and/or by decreased water reabsorption by kidney at a constant amount of penetrated proteins. This latter 'dilution' effect seems however to play a minor role (i) because total water balance and/or urine excretion varies to a much less extent compared with the decrease in total protein abundance detected [4]; and (ii) because the protein composition alters very strongly reflecting marked changes of the underlying physiology. Upon simple dilution one would expect only weak alterations of the protein composition.

For further analysis we used centralized abundance profiles as standard by subtracting the mean abundance value of each profile from the raw profile data. Positive and negative values consequently define the range of over- and under-presence of each protein species relatively to its mean value, respectively. Such centralized data accent alterations of protein expression independent of its absolute expression level. The SOM algorithm (see below) clusters profiles of proteins showing similar changes together. Hence, also invariantly high and invariantly low expressed proteins are clustered together. To analyze the absolute abundance level of the data we also used data without centralization. Detailed results of this analysis are presented in the supplementary text.

### SOM machine learning

We used an analysis pipeline based on the R-program opoSOM developed previously for high-throughput gene expression analysis [13,14]. It transforms the abundance values of all proteins measured into an abundance landscape per state. It serves as fingerprint portrait of the respective proteomic phenotype. The program also performs a series of useful downstream analysis tasks such as sample similarity-, differential feature selection- and gene set enrichment-analyses.

After appropriate initialization (see [14]) the SOM-algorithm distributes the proteins over a 40x 40 two-dimensional quadratic grid such that each protein profile is associated with the most similar grid point using the Euclidian distance as criterion. The grid points are called 'meta features'. Then the method iteratively adjusts the meta-feature profiles in small increments to agree better with the observed protein profiles. In consequence, the resulting two-dimensional map of meta-profiles optimally covers all protein profiles observed experimentally. Moreover, the map becomes self-organized, which means that proteins of similar profiles are clustered together, whereas proteins with distinct abundance profiles localize in different regions of the map.

The training thus translates the abundance data given as N × M matrix (N = 2037: number of proteins, M= 24 number of time points in mean volunteer) into a K × M matrix (K = 1600: number of meta-features). Each proteomic phenotype is visualized by color-coding the grid points in the two-dimensional grid of meta-features according to their abundance values from red to blue for high to low abundance values, respectively. Neighbored meta-features tend to be colored similarly owing to their similar profiles. In consequence the obtained mosaic images show a smooth texture with red and blue spot-like regions referring to clusters of over- and under-expressed proteins, respectively.

The SOM portraying methods has been applied before to different omics data including also proteomics data for MALDI-typing [18] (see also [19] and references cited therein). In extensive benchmark tests we showed that SOM outperforms alternative methods for dimension reduction of high-dimensional data [13]. Finally, parameter settings for optimal performance of the methods have been systematically studied before [13,14,19].

### Spot module selection, enrichment analysis and Beta correlation testing

To identify groups of co-expressed proteins we applied an over- and under-expression spot module selection method: It first averages each meta-feature value over all individual expression states considered and then selects the maximum and minimum 2-percentile of them, respectively. Then the spot-modules were defined as closed areas of adjacent, i.e. mutually connected meta-features in the map. Alternatively we tested two different module selection methods based on correlation and K-means clustering, respectively (see supplementary text).

Proteins from the same module are co-abundant in the experimental series and define a functional module according to the 'guilt-by-association' principle [20]. We applied *gene set enrichment analysis *to discover the functional context of the module using a data base of a few thousand predefined gene sets according to gene ontology (GO) classification as described in [14]. Enrichment scores are calculated using either Fishers exact test or the 'gene set enrichment Z-score' (GSZ) as proposed in [21]. The former score estimates the probability that the number of proteins from the set is found in the list of proteins in a module given the total number of proteins studied. The GSZ-score in addition considers the degree of overexpression of the proteins in the spot (see also [14] for details).

Interrelations between the spot modules are characterized in terms of the *weighted topological overlap network *(wTO) based on correlations between the meta-features as described in [12]. It considers not only direct correlations between all pairwise combinations of meta-features in the spots but also 'mediated' ones acting via all possible third meta-features in the map [22].

We adapted a multi-test-adjusted *correlation test *based on beta-test statistics as proposed previously [23] to estimate the significance of concerted expression of the proteins in each of the modules identified. This test calculates the significance that the group of proteins collected in a given module shows concerted abundance profiles. Significance is estimated using the beta value of each spot. It is defined as the squared ratio of two sum correlations, namely of the sum correlation between the mean module profile and the single feature profiles of the module and the sum correlation between all single features of the module. Our method substitutes the single feature profiles by the profiles of the respective meta-features to reduce the computational efforts. The beta test statistics is transformed into a p-value which estimates the multi-test-adjusted probability of the null hypothesis, namely that the single protein expression values of the module do not correlate each with another. Details of the method are given in the Supplementary Text section provided as Additional file 1.

### Pathway signal flow analysis of selected KEGG pathways

The Pathway Signal Flow (PSF) algorithm evaluates the changes in signal flows for a given pathway depending on the pathway topology and relative protein expression measured [24]. Particularly, it evaluates how a signal from network inputs spreads downstream from source nodes to sink nodes depending on the relative expression of the proteins forming the nodes and the types of interactions between them [25]. The more changes in the pathway flow are observed, the more it is likely that the given pathway will be involved into biological processes underlying the phenotypic differences between the conditions studied. The relative expression of a node is calculated as the mean of the relative abundance (fold change) of all items in the given node. The PSF method uses Kyoto Encyclopedia of Genes and Genomes (KEGG) Pathway database as the source of molecular pathway information [26]. We compared PSF time profiles with the time profiles of selected modules to assign the respective biological functions as described in Additional file 1.

### Time trajectories

Time trajectories aim at visualizing the time-dependent changes of the proteomics phenotypes studied. We applied standard sample similarity analysis using 2^nd ^level SOM and independent component analysis (ICA). Both methods project the samples into 'similarity space' which allows establishing the trajectory as the sequence of subsequent time points. Similarity analysis compares the protein expression states as seen by the SOM portraits. It uses the abundance of meta-features as the input data, which has the advantage of improving the representativeness and resolution of the results [13]. We applied 2^nd ^level SOM analysis as proposed in [27] to visualize the similarity relations between the samples. This method has the advantage that it projects also high-dimensional multivariate data into two dimensions which allows their straightforward evaluation. Its disadvantage is that the obtained phase space is scaled non-linearly and non-orthogonally with respect to different, mutually independent variables. We therefore also applied ICA [28] to the SOM meta-feature data using the R-package 'fastICA'. It distributes the samples in the phase space spanned by the components of minimal mutual statistical dependence. These components point along the directions of maximum information content in the data which is estimated by their deviation from a (non-informative) normal distribution [29].

## Competing interests

The authors declare that they have no competing interests.

## Authors' contributions

Conceived and designed the experiments: IML, ENN; performed the experiments and performed primary LC/MS-proteomics analysis: AK, IP, ENN, IML, LKP, conceived and designed downstream proteomics analysis (SOM and pathway analysis): HB, HW, AA, VI; NAK; performed downstream analysis: HB, HW, AA, KL, EST; wrote the paper: HB, IL, AK.

## Supplementary Material

Additional file 1 **Supplementary text includes supplementary methods, results, figures and tables**. Click here for file

Additional file 2 **Lists of differentially expressed proteins in the overexpression spot modules**. Click here for file

Additional file 3 **List of proteins in the K-means clusters segmented in the SOM of absolute protein abundance data**. Click here for file

Additional file 4 **Single volunteer proteomics data**. Click here for file

Additional file 5 **Protein abundance matrix used for SOM analysis**. Click here for file

## Declarations

Publication of this article has been funded in part by the BMBF within the frame of the German-Russian network of Bioinformatics and Biotechnology (the SOM analysis); Russian Science Foundation grant No 14-24-00114 (AK, IP and EN acknowledge the support in performing of all mass spectrometry measurements); Russian Science Foundation grant No 14-24-00123 (EST, VAI and NAK acknowledge the support in performing of the computer analysis of biological pathways and processes).

This article has been published as part of *BMC Genomics *Volume 15 Supplement 12, 2014: Selected articles from the IX International Conference on the Bioinformatics of Genome Regulation and Structure\Systems Biology (BGRS\SB-2014): Genomics. The full contents of the supplement are available online at http://www.biomedcentral.com/bmcgenomics/supplements/15/S12.

## References

[B1] Ortiz-Melo D Coffman Thomas M A Trip to Inner Space: Insights into Salt Balance from Cosmonauts Cell metabolism 2013 17 1 1 2 10.1016/j.cmet.2012.12.009 23312276

[B2] Mozaffarian D Fahimi S Singh GM Micha R Khatibzadeh S Engell RE Lim S Danaei G Ezzati M Powles J Global Sodium Consumption and Death from Cardiovascular Causes New England Journal of Medicine 2014 371 7 624 634 10.1056/NEJMoa1304127 25119608

[B3] Rakova N Jüttner K Rauh M Dahlmann A Goller U Beck L Agureev A Vassilieva G Lenkova L Johannes B Wabel P Moissl U Vienken J Gerzer R Eckardt KU Müller DN Kirsch K Morukov B Luft FC Titze J Ultra long-term sodium balance studies during the Mars500 campaign Aktuel Ernahrungsmed 2012 37 03 P9_5

[B4] Rakova N Jüttner K Dahlmann A Schröder A Linz P Kopp C Rauh M Goller U Beck L Agureev A Vassilieva G Lenkova L Johannes B Wabel P Moissl U Vienken J Gerzer R Eckardt KU Müller Dominik N Kirsch K Morukov B Luft Friedrich C Titze J Long-Term Space Flight Simulation Reveals Infradian Rhythmicity in Human Na+ Balance Cell metabolism 2013 17 1 125 131 10.1016/j.cmet.2012.11.013 23312287

[B5] Titze J Larina IM Garib K Kirsch KO Maye A Lang R Gunga HK Johanes B Gochlen-Koch H Kim E Monitoring of Sodium Balance during Long-Term Isolation of Humans in a Ground-Based Space Station Model Hum Physiol 2003 29 5 595 605 10.1023/A:1025820118198

[B6] Kleinewietfeld M Manzel A Titze J Kvakan H Yosef N Linker RA Muller DN Hafler DA Sodium chloride drives autoimmune disease by the induction of pathogenic TH17 cells Nature 2013 496 7446 518 522 10.1038/nature11868 23467095 PMC3746493

[B7] Machnik A Neuhofer W Jantsch J Dahlmann A Tammela T Machura K Park JK Beck FX Muller DN Derer W Goss J Ziomber A Dietsch P Wagner H van Rooijen N Kurtz A Hilgers KF Alitalo K Eckardt KU Luft FC Kerjaschki D Titze J Macrophages regulate salt-dependent volume and blood pressure by a vascular endothelial growth factor-C-dependent buffering mechanism Nat Med 2009 15 5 545 552 10.1038/nm.1960 19412173

[B8] Marvar PJ Gordon FJ Harrison DG Blood pressure control: salt gets under your skin Nat Med 2009 15 5 487 488 10.1038/nm0509-487 19424204

[B9] Valeeva OA Pastushkova LK Pakharukova NA Dobrokhotov IV Larina IM Variability of urine proteome in healthy humans during a 105-day isolation in a pressurized compartment Hum Physiol 2011 37 3 351 354 10.1134/S0362119711030157 21780686

[B10] Larina IM Kolchanov NA Dobrokhotov IV Ivanisenko VA Demenkov PS Tiys ES Valeeva OA Pastushkova LK Nikolaev EN Reconstruction of associative protein networks connected with processes of sodium exchange regulation and sodium deposition in healthy volunteers based on urine proteome analysis Hum Physiol 2012 38 3 316 323 10.1134/S0362119712030061 22830250

[B11] Pastushkova LK Kireev KS Kononikhin AS Tiys ES Popov IA Starodubtseva NL Dobrokhotov IV Ivanisenko VA Larina IM Kolchanov NA Nikolaev EN Detection of Renal Tissue and Urinary Tract Proteins in the Human Urine after Space Flight PLOS one 2013 8 8 e71652 10.1371/journal.pone.0071652 23967230 PMC3742504

[B12] Hopp L Wirth H Fasold M Binder H Portraying the expression landscapes of cancer subtypes: A glioblastoma multiforme and prostate cancer case study Systems Biomedicine 2013 1 2

[B13] Wirth H Loeffler M von Bergen M Binder H Expression cartography of human tissues using self organizing maps BMC Bioinformatics 2011 12 306 10.1186/1471-2105-12-306 21794127 PMC3161046

[B14] Wirth H von Bergen M Binder H Mining SOM expression portraits: Feature selection and integrating concepts of molecular function BioData Mining 2012 5 18 10.1186/1756-0381-5-18 23043905 PMC3599960

[B15] Liu X Yu X Zack D Zhu H Qian J TiGER: A database for tissue-specific gene expression and regulation BMC Bioinformatics 2008 9 1 271 10.1186/1471-2105-9-271 18541026 PMC2438328

[B16] Agron IA Avtonomov DM Kononikhin AS Popov IA Moshkovskii SA EN N Accurate mass tag retention time database for urine proteome analysis by chromatography-mass spectrometry Biochemistry (Mosc) 2010 75 5 636 641 10.1134/S0006297910050147 20632944

[B17] Ishihama Y Rappsilber J Andersen JS M M Microcolumns with selfassembled particle frits for proteomics J Chromotography A 2002 979 1-2 233 239 10.1016/S0021-9673(02)01402-4 12498253

[B18] Wirth H von Bergen M Murugaiyan J Rösler U Stokowy T Binder H MALDI-typing of infectious algae of the genus Prototheca using SOM portraits Journal of Microbiological Methods 2012 88 1 83 97 10.1016/j.mimet.2011.10.013 22062088

[B19] Binder H Wirth H Khosrow-Pour M Analysis of large-scale OMIC data using Self Organizing Maps Encyclopedia of Information Science and Technology 2014 Third Edition IGI global 1642 1654 in press

[B20] Quackenbush J Microarrays--Guilt by Association Science 2003 302 5643 240 241 10.1126/science.1090887 14551426

[B21] Toronen P Ojala P Marttinen P Holm L Robust extraction of functional signals from gene set analysis using a generalized threshold free scoring function BMC Bioinformatics 2009 10 1 307 10.1186/1471-2105-10-307 19775443 PMC2761411

[B22] Zhang B Horvath S A general framework for weighted gene co-expression network analysis Statist Appl Genet Mol Biol 2005 5 1 17 10.2202/1544-6115.1128 16646834

[B23] Läuter J Horn F Rosolowski M Glimm E High-dimensional data analysis: Selection of variables, data compression and graphics - Application to gene expression Biometrical Journal 2009 51 2 235 251 10.1002/bimj.200800207 19358215

[B24] Arakelyan A Nersisyan L KEGGParser: parsing and editing KEGG pathway maps in Matlab Bioinformatics 2013 29 4 518 519 10.1093/bioinformatics/bts730 23292739

[B25] Arakelyan A High-throughput Gene Expression Analysis Concepts and Applications Sequence and Genome Analysis II - Bacteria, Viruses and Metabolic Pathways 2013 Hong Kong: iConcept Press

[B26] Kanehisa M Goto S KEGG: kyoto encyclopedia of genes and genomes Nucleic Acids Res 2000 28 1 27 30 10.1093/nar/28.1.27 10592173 PMC102409

[B27] Guo Y Eichler GS Feng Y Ingber DE Huang S Towards a Holistic, Yet Gene-Centered Analysis of Gene Expression Profiles: A Case Study of Human Lung Cancers Journal of Biomedicine and Biotechnology 2006 2006 Article ID 69141 10.1155/JBB/2006/69141 PMC1698264 17489018

[B28] Hyvärinen A Oja E Independent component analysis: algorithms and applications Neural Networks 2000 13 4-5 411 430 10.1016/S0893-6080(00)00026-5 10946390

[B29] Liebermeister W Linear modes of gene expression determined by independent component analysis Bioinformatics 2002 18 1 51 60 10.1093/bioinformatics/18.1.51 11836211

