# Supplementary material for: Time-course human urine proteomics in space-flight simulation experiments
Source: BMC Genomics. 2014 Dec 19;15(Suppl 12):S2. doi: 10.1186/1471-2164-15-S12-S2 (PMC4303941; doi:10.1186/1471-2164-15-S12-S2)
Supplement: Additional file 1 — Supplementary text includes supplementary methods, results, figures and tables. [file 1471-2164-15-S12-S2-S1.PDF]

Supplementary Text

Time-course human urine proteomics in space-flight simulation experiments

Hans Binder[1]*, Henry Wirth[1], Arsen Arakelyan[2], Kathrin Lembcke[1], Evgeny S. Tiys[3], Vladimir A. Ivanisenko[3], Nicolay A. Kolchanov[3], Alexey Kononikhin[4,6], Igor Popov[5,6], Evgeny N. Nikolaev[4,5,6,7]*, Lyudmila Kh. Pastushkova[8], Irina M. Larina[8]

[1] Interdisciplinary Centre for Bioinformatics, Universität Leipzig, Leipzig, Germany
[2] Institute of Molecular Biology NAS RA; Yerevan, Armenia
[3] Institute of Cytology and Genetics SB RAS, Novosibirsk, Russia
[4] Talrose Institute for Energy Problems of Chemical Physics, RAS, Moscow, Russia
[5] Emanuel Institute for Biochemical Physics, RAS, Moscow, Russia
[6] Moscow Institute of Physics and Technology, Dolgoprudnyi, Russia
[7] Skolkovo Institute of Science and Technology, Skolkovo, Russian Federation
[8] Institute of Biomedical Problems – Russian Federation State Scientific Research Center RAS, Moscow, Russia

*Corresponding authors:
Hans Binder. University of Leipzig, Interdisciplinary Centre for Bioinformatics (IZBI), 04107 Leipzig, Haertelstr. 16-18, Germany; e-mail: binder@izbi.uni-leipzig.de; Tel.: +49-341-9716671; Fax: +49-341-9716679

E.N. Nikolaev. Institute for Energy Problems of Chemical Physics Russian Academy of Sciences, Leninskij pr.38 k.2, 119334 Moscow, Russia, e-mail: ennikolaev@rambler.ru

Content

TIME-COURSE HUMAN URINE PROTEOMICS IN SPACE-FLIGHT SIMULATION EXPERIMENTS 1

1 SUPPLEMENTARY METHODS 3

1.1 BETA CORRELATION TEST OF SPOT MODULES 3
1.2 PATHWAY SIGNAL FLOW ANALYSIS 5

2 SUPPLEMENTARY RESULTS 8

2.1 INDEPENDENT COMPONENT ANALYSIS 8
2.2 SUPPORTING MAPS: POPULATION, VARIANCE AND ENTROPY MAPS 9
2.3 SPOT MODULE SELECTION 11
2.4 ESTIMATING BETA SIGNIFICANCE OF META-FEATURES AND SPOT MODULES 12
2.5 ALTERNATIVE SPOT SELECTION: CORRELATION AND K-MEANS CLUSTERING 14
2.6 OVEREXPRESSION SPOT ANALYSIS: PROTEIN LISTS 16
2.7 SPOT RELATED PATHWAY SIGNAL FLOWS 16
2.8 MAPPING OF SELECTED PROTEIN GROUPS 19
2.9 SINGLE VOLUNTEER ANALYSIS 21
2.10 ORGAN RELATED PROTEIN EXPRESSION 26
2.11 MAPPING AND PROFILING OF SELECTED GO PROTEIN SETS 29
2.12 SOM ANALYSIS OF ABSOLUTE PROTEIN EXPRESSION LEVELS 32

3 REFERENCES 38

1 Supplementary methods

1.1 Beta correlation test of spot modules

We consider a spot-module taken from the SOM map representing a cluster of s=1,…,S meta-features which, in turn, are 'micro-clusters' of n_s single features. In total, the spot thus contains $P=\sum_{s=1}^{S} n_s$ single features. Meta features and single features are given as time profiles of protein expression values (E_{pt}, E_{st}^{meta}, respectively) centralized with respect to their mean value, averaged over all time points t=1,…, T of the measurement:

$$\begin{aligned} \Delta E_{st}^{meta} &= E_{st}^{meta} - \tfrac{1}{T}\sum_{t=1}^{T} E_{st}^{meta} \quad and \\ \Delta E_{pt} &= E_{pt} - \tfrac{1}{T}\sum_{t=1}^{T} E_{pt} \quad , \quad p=1...P \end{aligned} , \qquad (1)$$

respectively. The ΔE thus define differential expression values given in units of the mean binary detection level (E=0 for absent and E=1 for present). Each spot cluster is characterized by the spot-averaged expression profile

$$\Delta E_t^{spot} = \frac{1}{S\cdot P}\sum_{s=1}^{S} n_S \cdot \Delta E_{st}^{meta} \approx \frac{1}{P}\sum_{p=1}^{P} \Delta E_{pt} \; . \qquad (2)$$

The latter equation considers the fact, that each meta feature profile is given to a good approximation by the average over all single features of the microcluster. The meta-feature profiles in Eq. (2)are weighted with the respective numbers of single features per meta-feature.

We now aim at testing whether ΔE_t^{spot} is significantly co-expressed with the set of single expression values contained in the meta-features of the spot Δe_{pt} or not. For this purpose we make use of the correlation test introduced previously [1, 2]: It states that the correlation of a set of variables x_{tp}(p=1,…,P; t=1,…,T) with a selected variable y_t is significant at level α if it meets the condition

$$\begin{aligned} beta &\geq BETA_{1-\alpha}(\tfrac{1}{2}, \frac{T-2}{2}) \quad with \\ beta &\equiv \frac{\left((Y-\overline{Y})'(Z-\overline{Z})\right)^2}{(Y-\overline{Y})'(Y-\overline{Y})\cdot(Z-\overline{Z})'(Z-\overline{Z})} \end{aligned} \qquad (3)$$

and

$$X = \begin{pmatrix} x_{11} & \cdots & x_{1P} \\ \vdots & \ddots & \vdots \\ x_{T1} & \cdots & x_{TP} \end{pmatrix} \quad , \quad Z = XD \quad and \quad Y = \begin{pmatrix} y_1 \\ \vdots \\ y_T \end{pmatrix} . \qquad (4)$$

The diagonal matrix D with the elements $d_{pp} = \mathrm{var}(X_p)^{-1/2} \equiv \left(\left(X_p - \overline{X_p}\right)'\left(X_p - \overline{X_p}\right)\right)^{-1/2}$ z-normalizes X_p, the column vectors of X. $\overline{X_p} \equiv \tfrac{1}{T}\sum_{t=1}^{T} x_{tp}$ denotes the respective column-average (in Eq. (3), analogously for Y and Z). Eq. (3)states that the beta test statistics is distributed according to the beta-distribution,

$$f(beta) = beta^{-1/2} \cdot (1-beta)^{T/2-2} \,/\, BETA_1(\tfrac{1}{2}, \frac{T-2}{2}) \,.$$

Eqs. (3) - (4) apply to Eqs. (1) - (2) if one simply sets

$$y_t = \Delta E_t^{spot} \quad and \quad x_{tp} = \Delta E_{pt} \;. \tag{5}$$

The test statistics can be expressed as

$$beta = q^2 \quad with \quad q = \frac{\sum_{p=1}^{P} r_{yp}}{\left(\sum_{p=1}^{P} \sum_{p'=1}^{P} r_{pp'} \right)^{1/2}} \;, \tag{6}$$

where q is the ratio of two sum correlations, namely the correlation between Y and Z and the correlation between the columns of Z, i.e.

$$r_{yp} = \frac{(Y-\overline{Y})'(Z_p - \overline{Z_p})}{\mathrm{var}(Y)^{1/2}} \quad and \quad r_{pp'} = (Z_p - \overline{Z_p})'(Z_{p'} - \overline{Z_{p'}}) \;, \tag{7}$$

respectively. Then the p-value estimates the probability that the single features in the spot cluster are not significantly correlated and thus not co-expressed,

$$beta \sim BETA_{1-p}(\tfrac{1}{2}, \frac{T-2}{2}) \;. \tag{8}$$

Let us assume

$$\sum_{p=1}^{P} r_{yp} \approx \tfrac{1}{P} \sum_{s=1}^{S} n_s r_{ys} \quad and \quad \sum_{p=1}^{P} \sum_{p'=1}^{P} r_{ij} \approx \tfrac{1}{P^2} \sum_{s=1}^{S} \sum_{s'=1}^{S} n_s n_{s'} \cdot r_{ss'} \;, \tag{9}$$

to a good approximation, i.e. we replace the sum correlations of the single features with that of the meta features and weight them with their populations. Accordingly, the elements of the X-matrix in Eq. (5) should be replaced with

$$x_{sp} = \Delta E_{ps}^{meta} \;. \tag{10}$$

The beta test was also applied to the meta-features of the map. In this case Eq. (3) applies with

$$y_t = \Delta E_{st}^{meta} \;, \quad x_{tp} = \Delta E_{pt} \quad and \quad P = n_{meta} \,. \tag{11}$$

It relates the sum correlation of the single features with the meta feature to the mutual sum correlation of the single features.

1.2 Pathway signal flow analysis

Biological pathways are directed and spatially defined sets of bio-molecular physical and regulatory interactions that represent information propagations (or signal flow) leading to functional realizations of biological processes. Thus, molecular pathways can be represented as directed graphs with nodes corresponding to genes, proteins, and compounds and edges depicting directed relationships between nodes (Figure S 1).

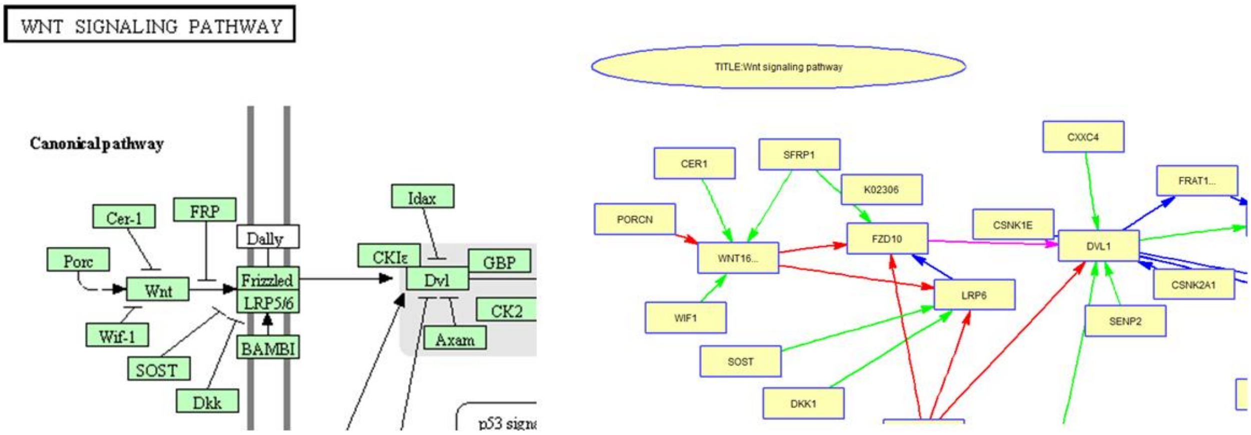

Figure S 1: Fragment of the KEGG Pathway Wnt-signaling pathway map image and corresponding graph object parsed from the KGML file.

Graph structure representation is often used to store pathway information in machine-readable format, usually, as xml files. For KEGG pathway images graph structures are stored in KGML (KEGG pathway xml format) files that can be used for automated analysis of pathways. Using KEGG parser - a Matlab tool for parsing and editing pathways maps we obtained graphs object for 258 pathways containing in KEGG pathway database[3]. Different interaction types present in KEGG pathways (i.e. phosphorylation, de-phosphorylation, ubiquitination, methylation, glycolysation, indirect effect, binding, etc.) were generalized in terms of their functional effect into two types: activation and inhibition.

Pathway topology is an important characteristic of a pathway and is pivotal for its functioning. The terminal (source and sink) genes seem to be more important from the viewpoint of signal transduction, than genes located in the middle of the pathway. On the other hand, pathway branching and mean number of interactions per gene may also highly influence gene-expression dependent signal transduction. Next parameter influencing pathway activation is the expression level of individual genes/gene products ingiven pathway. Expression, being the marker of gene/protein activity in the cell, is estimated based on the amount of mRNA/protein that has been synthesized from the given gene. Thus based on these two parameters it is possible to identify how pathway activity can be changed compared to reference state.

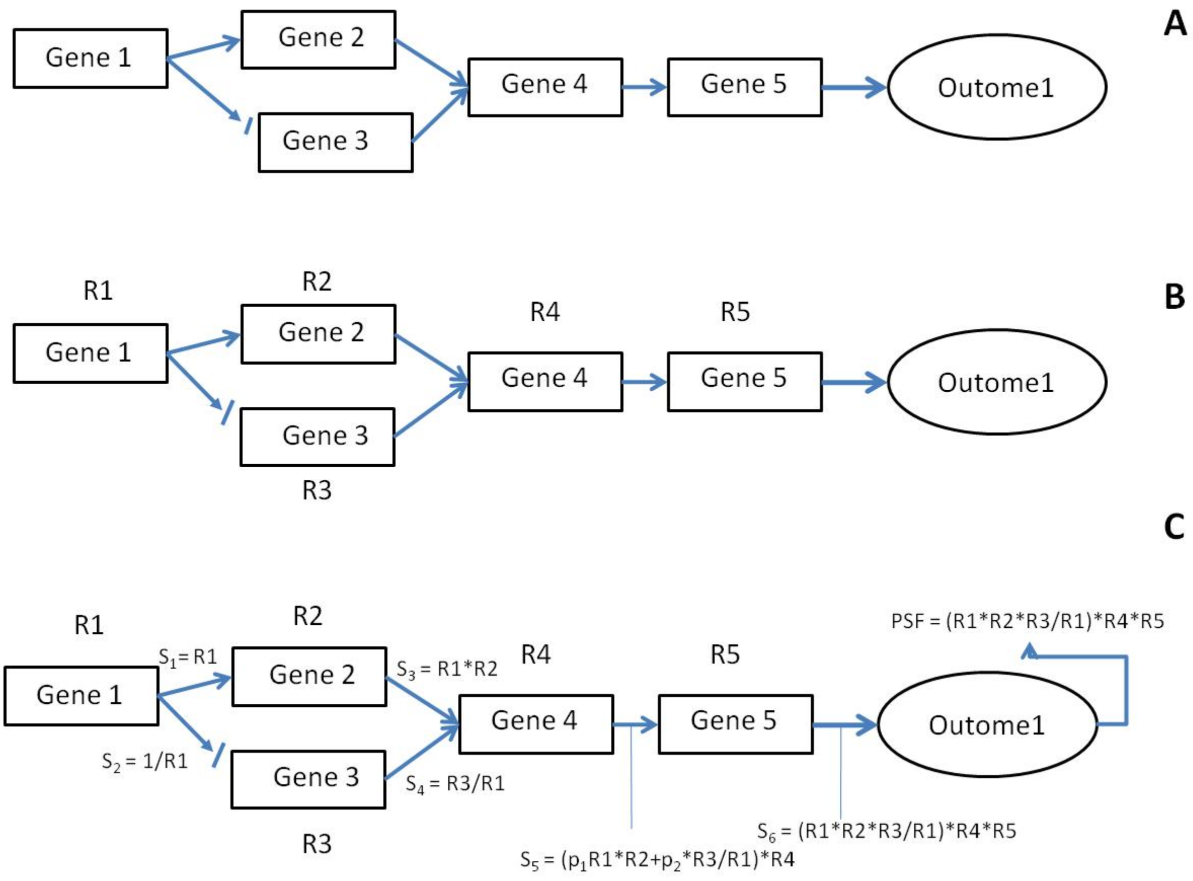

Figure S 2: Schematic representation of PSF algorithm workflow on hypothetic pathway graph.

Figure S 2 schematically shows main steps of pathway signal flow (PSF) calculation. It starts with topological sorting of pathway graphs to identify source (input/ gene1) and sink (output/outcome) nodes. Feedback loop containing pathways is sorted partially. After this step an initial unity signal is applied to the pathway source node(s). The signal flow at the outgoing edge is set equal to the product of input signal and relative expression of source node:

$$S_i = S_{i-1}^{k} R_i,$$

where R_i is the relative expression of node i; S_{i-1}^{k} is signal flow at the node incoming edges. k defines the activation exponent with k = 1, if node (i-1) activates node i and k = -1, if node (i-1) inhibits node i. If a node has two or more inputs, its relative expression is partitioned based on the value of input signals and then it is summarized. Signal flow at the sink nodes of a pathway is considered as pathway signal flow (PSF). Significance of pathway flow perturbation is calculated by reshuffling node relative expressions 1000 times and constructing the empirical distribution.

We compared temporal profiles of spot expressions with profiles of pathway flow perturbations in order to identify pathways associated with each spot cluster derived from SOM analysis. Temporal association of pathway signal perturbations with spot expression were performed using regression. R^2>0.8 cutoff for association was chosen.

It should be noted that many pathways have more than one functional outcome. For example activation of WNT signaling pathway (Figure S 3) may cause activation of Cell Cycle, Adherens junction pathways, proteolysis, gene expression trough activation of NFAT, etc. Thus differential activation of genes belonging to different pathway branches may lead to perturbations of different outcomes. In such multi-branch pathways PSF and its significance is calculated for each functional outcome separately.

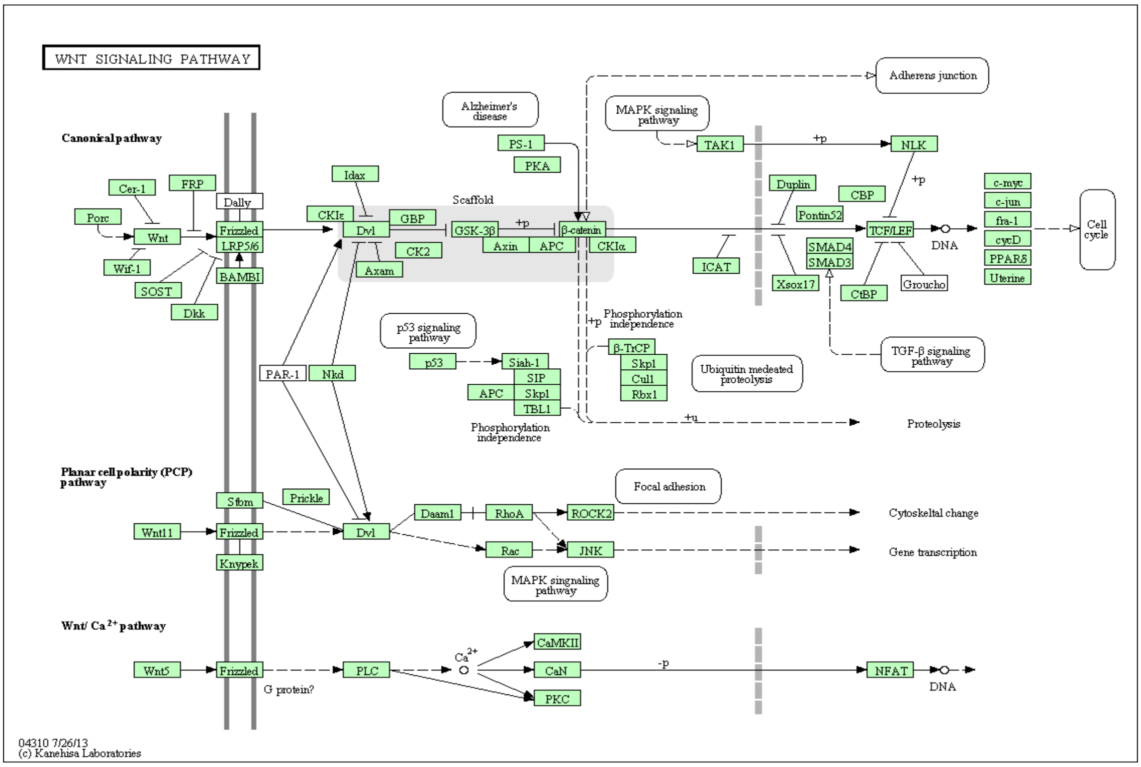

Figure S 3: Example of multi-branch pathway map.

2 Supplementary results

2.1 Independent component analysis

The 2nd-level SOM presentation as used in the main paper is advantageous because it visualizes multivariate relations within a relatively simple two-dimensional image using however distorted non-linear distance metrics. We analyzed similarity relations using independent component analysis (ICA) projecting the samples in linear scale. Figure S 4 shows the sample trajectory in the three-dimensional space spanned by the first three independent components (IC1 – IC3) obtained after independent component analysis (ICA).
ICA virtually confirms the results obtained using 2nd level SOM presented in the main paper: During the experiment the samples move along the first independent component (IC1), until week 6 in one direction and afterwards backwards (‘early time range’). Both oppositely directed parts of the trajectory refer to the ranges of high and low salt consumption, respectively. They are shifted each to another along IC3 so that the system doesn’t reach its starting point after the experiment.

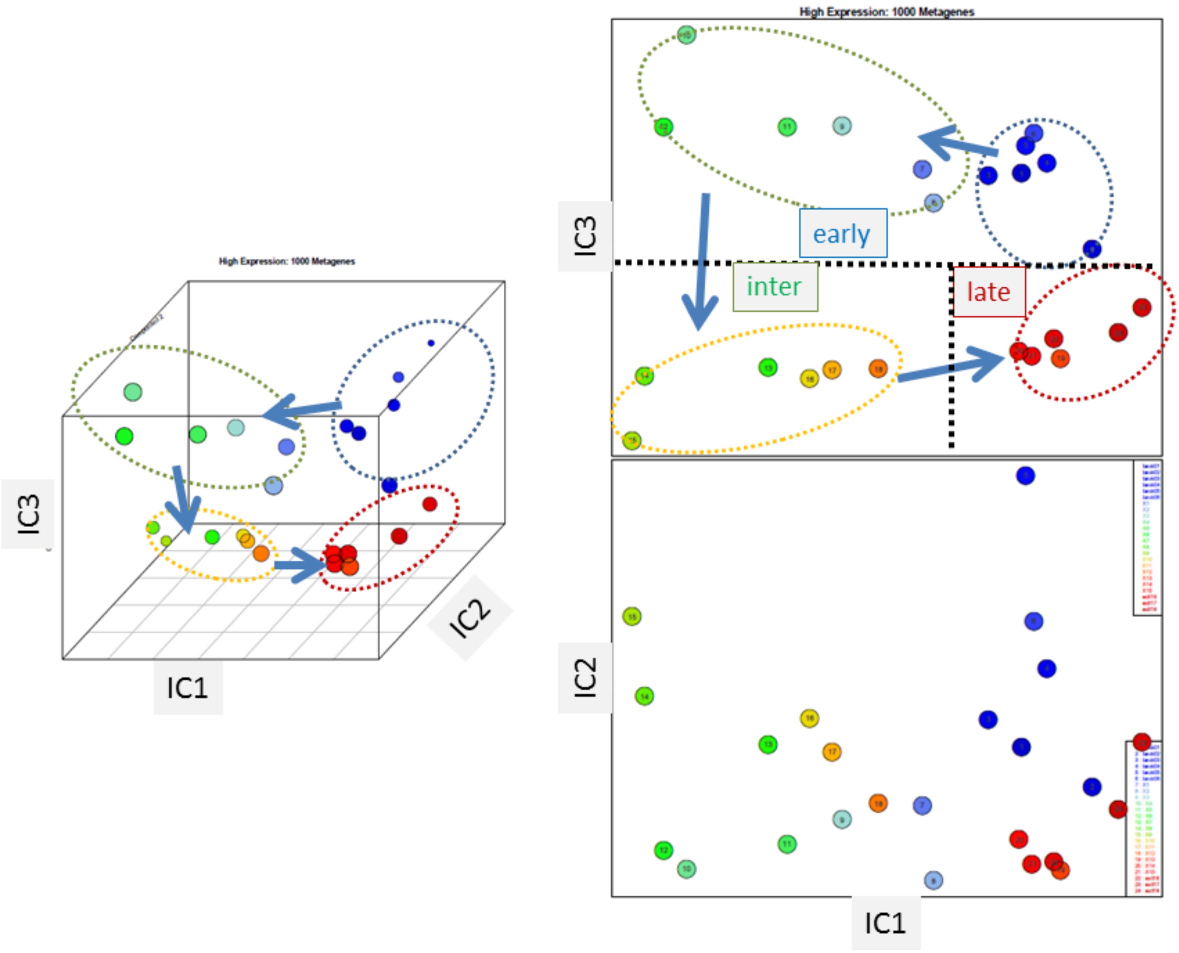

Figure S 4: Independent component analysis (ICA): The left part shows the three dimensional distribution of samples in the space spanned by the first three independent components IC1, IC2 and IC3. The right part shows two-dimensional projections into IC1/IC2 and IC1/IC3 planes.

2.2 Supporting maps: population, variance and entropy maps

The population map color codes the number of single features per meta-features in the map (Figure S 5). Empty meta-features not containing single features are colored in white. The single features mainly cluster into three regions which can be assigned to features up-regulated in the early and in the late (and the intermediate) time ranges and to single spiked and rare features as indicated. Most of the proteins refer to the former cluster (see the table in Figure S 5). The variance map visualizes the variance of each meta-feature profile, $\mathrm{var}_m = \sum_t \Delta E_{mt}^{meta\,2} / (T-1)$ (m=1…M denotes the number of meta features), using an appropriate color code (red to blue means high to low). It reveals that the highly variant profiles in the early_up and late_up clusters are separated by the region of relatively invariant single-spiked profiles.

The entropy map plots the standard entropy of each meta feature profile, $h_m = -\sum_{i=1}^{3} p_{mi} \log_2 p_{mi}$ where p_{mi} is the relative frequency of three levels of protein expression, overerexpression (i=1), underexpression (i=2) and non-differential expression (i=3), in the profile of meta feature m. To estimate p_{mi} we divided the expression values of the meta feature profiles in to three levels by application of a defined threshold (here the 25- and 75-percentile of all meta feature expression values was used). h_m is restricted to values in the interval [0, $\log_2 3$]. An entropy value of 0 represents a perfectly ‘ordered’ profile, where all meta-feature values are assigned to only one of the expression levels. Contrary, the maximum value of $\log_2 3 \approx 1.58$ is reached if the meta-feature values of the profile uniformly distribute over the three levels. The entropy is an information content measure by definition. A virtually invariant profile with a low entropy consequently reflects the fact that it is almost uninformative with respect to the time course of protein expression. Contrarily, a high entropy value means that the information content of the respective profiles is high. Note that variance and entropy reveal similar but partly also complementary properties of the meta-features: A low variant profile is typically less informative with low entropy too. A highly variant profile however can possess only medium entropy because it lacks maximum diversity (e.g. if it shows a high but constant differential expression). Finally, profiles of maximum diversity and thus maximum entropy usually of medium variance only. Comparison of the variance and entropy maps in Figure S 5 reveal an interesting substructure of the high variant areas: Particularly, one identifies a region of less diverse but highly differently expressed profiles in the to-right corner of the map.

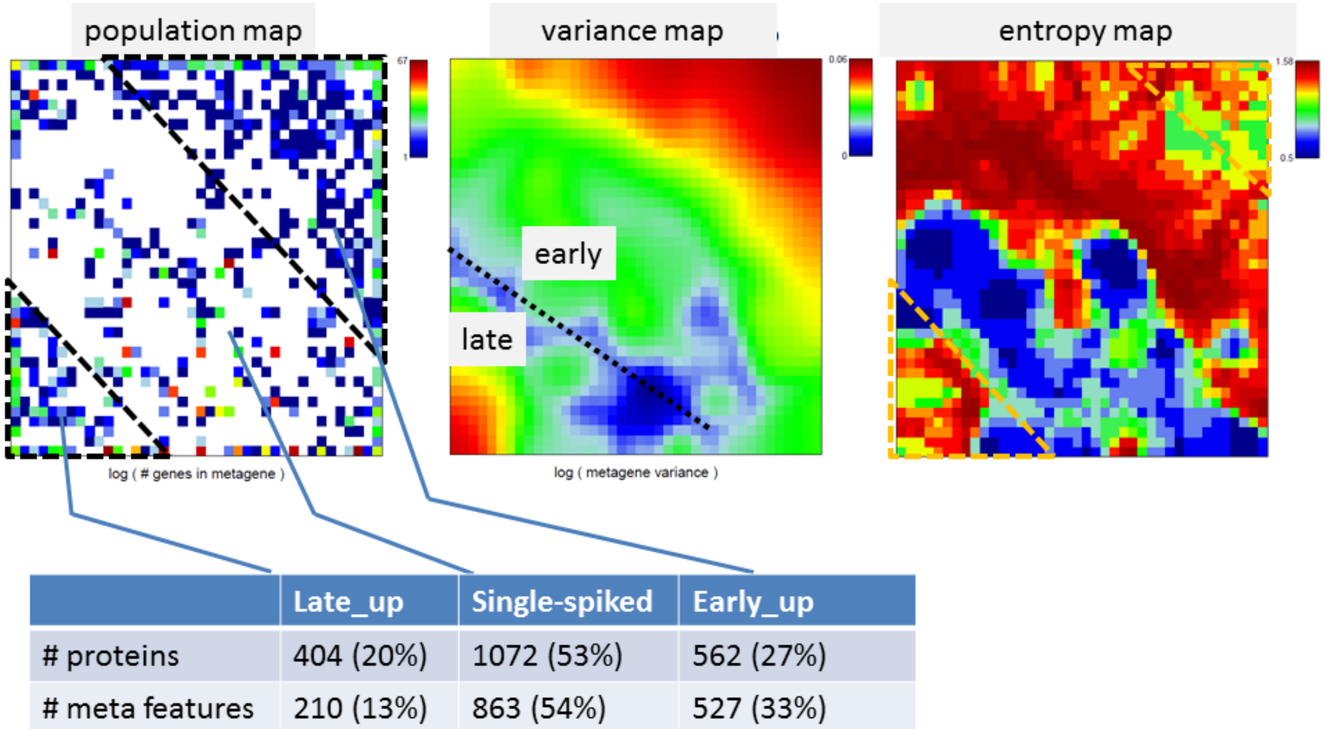

	Late_up	Single-spiked	Early_up
# proteins	404 (20%)	1072 (53%)	562 (27%)
# meta features	210 (13%)	863 (54%)	527 (33%)

Figure S 5: Supporting maps characterizing the meta-feature landscapes. The population map visualizes the occupation of meta features with single features (blue to red refers to 1 to 67 single feature/meta features, white are empty meta features). The variance map color codes the variance of the meta feature profiles red (high variance) to blue (small). The entropy map color codes the entropy of the meta-feature profiles from red (high entropy) to blue (low). The ranges referring to early and late time ranges are separated by areas of low populated, low variant and low entropy meta-features. Note that the entropy in the area of highest variance near the top right corner of the map is only medium because the respective profiles are less diverse than that in the ranges of intermediate variant meta-features.

2.3 Spot module selection

We compared three different methods to identify groups of co-expressed proteins that we call modules: (i) *Over- and under-expression spot-modules* are calculated by averaging each meta-feature value over all individual expression states considered and then selecting the maximum and minimum 2-percentile of them, respectively. Then the spot-modules were defined as closed areas of adjacent, i.e. mutually connected meta-features in the map. (ii) *Correlation spot modules* are calculated using a 'seed algorithm' starting with the pair of meta-features showing the largest Pearsons correlation coefficient between their profiles. Then adjacent meta-features are added to this module if the mutual correlation coefficient with the seed features exceeds a certain threshold (here 0.5). Otherwise a new seed-pair is selected among the still 'free' meta-features (i.e. that which are not assigned to another correlation module so far) defining a new module which grows by adding adjacent free meta-features using the same correlation threshold. This algorithm is repeated until no seed-pair satisfies the correlation criterion. (iii) *K-means cluster modules* are calculated by applying K-means clustering to the profiles of the meta-features using the *Euclidian* distance between them as similarity metrics. The desired number of cluster is set to the number of overexpression spots determined before. Note that K-means clustering assigns all meta-features to a certain cluster whereas overexpression and correlation clustering leaves a certain number of meta-features unassigned with respect to the clusters determined.

Figure S 6 (upper part) illustrates that the degree of area occupancy of the map increases in this order. In the supplementary material we present a series of so-called supporting maps which have been designed to analyze intrinsic properties of the SOM [4]. The population map shows that the proteins distribute heterogeneously over the map and preferentially accumulate in the regions of overexpression in the early (27 %) and late/intermediate (20 %) time ranges. About 53 % of the proteins are classified into invariant and 'single spiked' ones as will be discussed below. They are of limited interest here.

The overexpression spot criterion selects 388 (19% of all) proteins where about 178 (46% of selected) can be assigned to the interesting fraction of variable profiles, not referring to the invariant or single spiked proteins. The correlation and K-means cluster methods select 1,288 (63%) and 2,038 (100%) proteins, respectively, where however the fraction of interesting proteins increases only slightly to 63% (correlation spots) and 47% (K-means spots). The heat maps shown in Figure S 6 document that either of the methods well reproduces the time course of the system in terms of spot profiles. We applied beta-testing to estimate the significance of the protein clusters selected by the different methods (see next subsection). It turned out that spots selected in the upper right and lower left regions of the map, independently of the clustering method, collect clusters of proteins concertedly changing with time whereas proteins in the remaining regions in the map do virtually not.

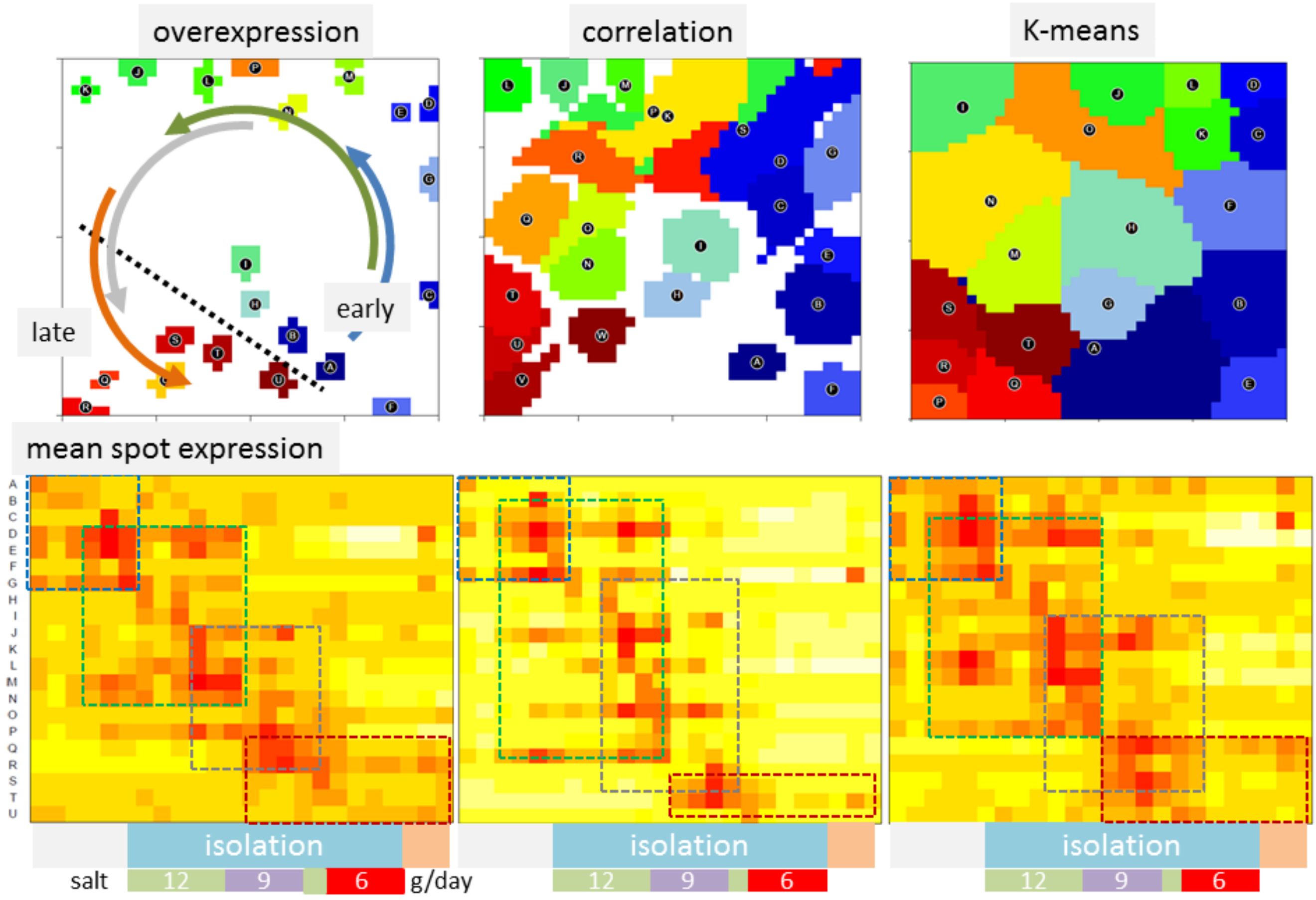

Figure S 6: Comparison of overexpression, correlation and K-means spot selection methods: Note that each method selects cluster-areas of different size and shape in the SOM map (see the colored areas in the respective maps). Temporal sequence of protein up-regulation follows roughly the spot trajectory as indicated by the arrows in the overexpression map. The heat maps show the mean abundance of each spot (red…high, white…low) sorted in temporal order in vertical direction. Essentially one finds analogous abundance patterns for all methods. All clusters are assigned by letters: Enlarged plots and lists of proteins in each of the overexpression spots are provided in additional file 2..

2.4 Estimating beta significance of meta-features and spot modules

The beta map in Figure S 7 color codes the beta value of each meta feature ($\log_{10}$-scale), thus estimating the degree of mutual correlation between the meta feature profile and the profiles of the associated single features (see Eq. (3)). It again identifies the late_up and early_up regions by large beta values. The spot significance maps shown in the lower panes in Figure S 6 color code the spot clusters obtained by means of three independent methods according to their $\log_{10}p$ values obtained by means of the beta test described above. The clusters are largely insignificant in the region of rare and single-spike profiles meaning that their meta-features are not co-expressed in terms of correlated profiles. Note that the p-value is governed by q^2 (see Eq. (6)) which scales with the variance of the meta-features (Eq. (7)). Hence, highly variant regions of the map (see Figure S 5) well match to regions of significant spot clusters.

Figure S 7 compares the beta-test significance maps of the mean-volunteer and the single volunteer analyses. The exact localization of the spots differs between both maps because they were obtained in independent training runs. The number of significant spots and their split into an early_up and a late_up cluster agree between both maps. On

the other hand, the number of insignificant single spike spots largely increases in the single volunteer map owing to the fact that these profiles mostly refer to proband-specific spikes.

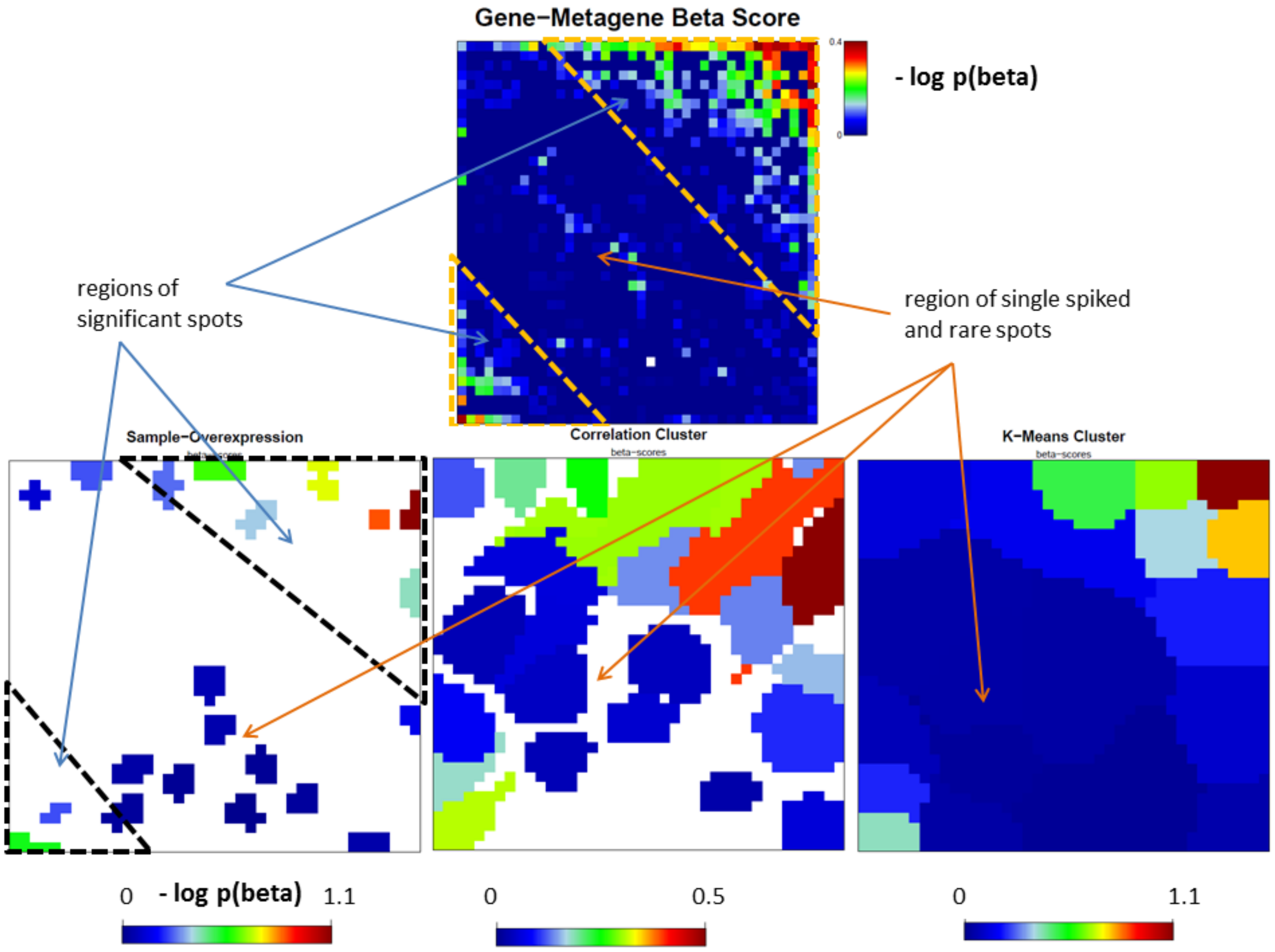

Figure S 7: Beta test significance maps of spots obtained using the overexpression, correlation and K-means clustering methods. Significance for each spot was estimated using the correlation beta test. Green to brown refers to $\log_{10}p<-0.5$ (overexpression and K-means clustering spots) and $\log_{10}p<-0.25$ (correlation cluster). Single spiked and rare spots are found in the central area of the map. They are insignificant in terms of correlated set of genes included in each of the spots. The beta map visualizes the log beta value of each meta-features (see Eq. (3), blue refers to small values, red to large ones).

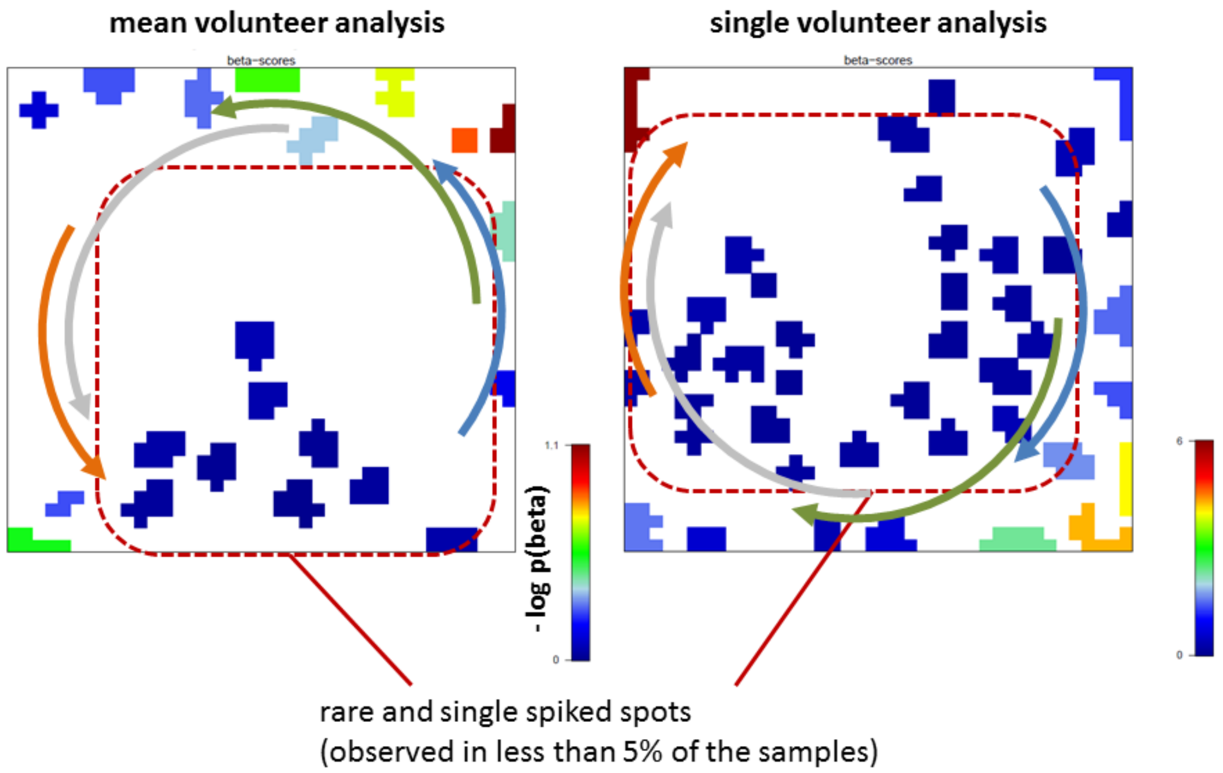

Figure S 8: Beta test significance maps of the overexpression spots obtained in the ‘mean volunteer analysis’ (left, see also Figure S 7, left panel) and ‘single volunteer analysis’ (right). In both maps insignificant spots accumulate in the central area of the map. Their number is much larger in the ‘single volunteer’-map (~30) than in the ‘mean volunteer’-map (~10) whereas the number of significant spots roughly agrees (8-10). Note also that spot overexpression changes with time either in counter-clockwise or in clockwise direction in both maps due to the independent SOM training of the data.

2.5 Alternative spot selection: correlation and K-means clustering

Figure S 9 shows the spot profiles of the significant expression modules obtained by means of the correlation clustering and K-mean clustering methods together with the most enriched gene set per module. Profiles and leading gene sets mostly agree between the different methods. Note that the K-means cluster modules contain the largest numbers of single proteins whereas the overexpression modules contain the least number of proteins with correlation clusters in between.

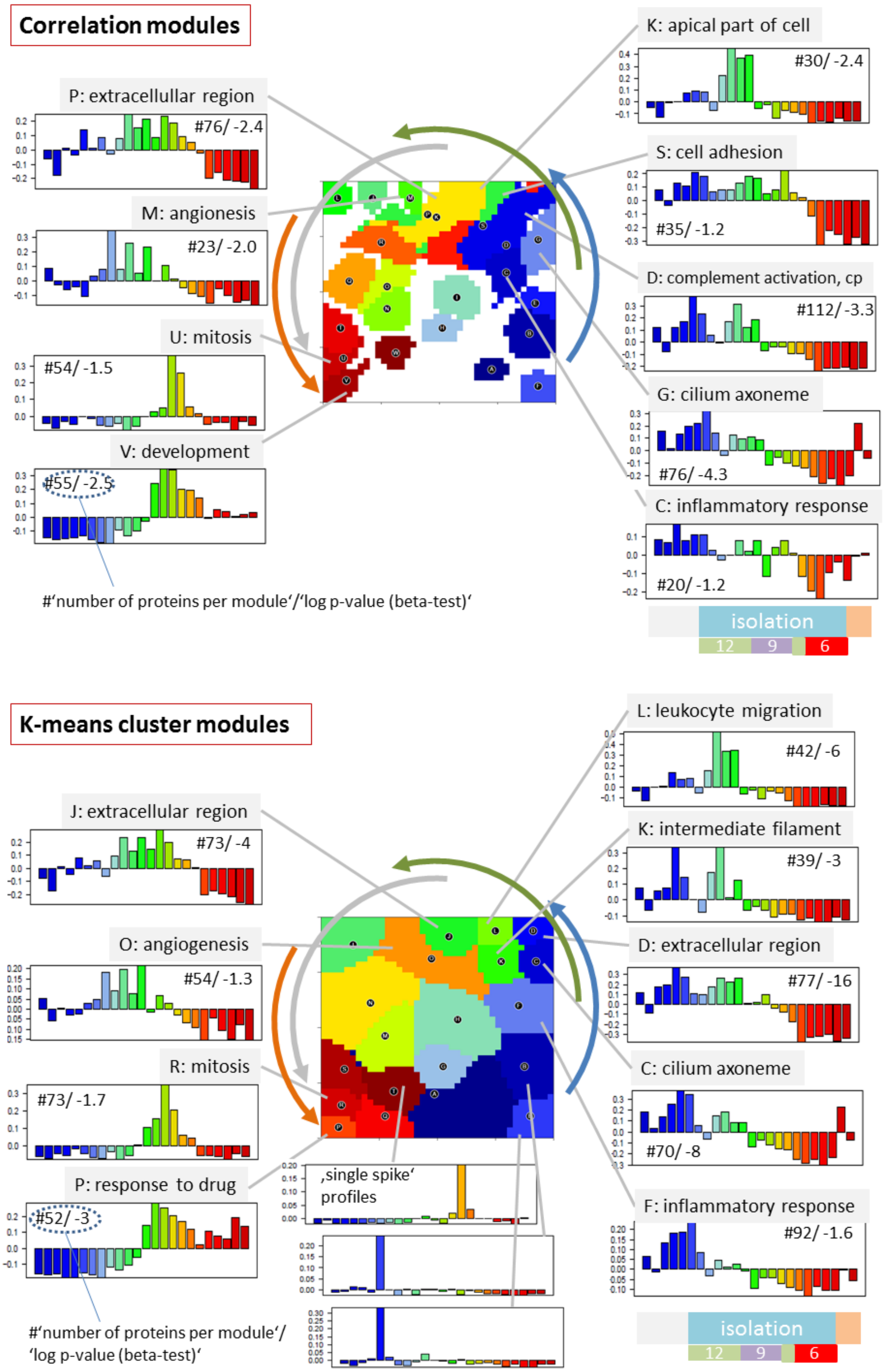

Figure S 9: Spot profiles and top enriched gene set per spot as seen by the correlation spot (part above) and K-mean clustering (part below) module selection methods. Selected single-spiked spots are shown in the K-means clustering map only.The vertical axis of the profiles is scaled in units of differential expression, ΔE, representing the mean binary detection level centralized with respect to the mean expression in all samples at all times (see supplementary text).

2.6 Overexpression spot analysis: protein lists

Figure S 10 lists the proteins in the overexpression spots ranked with decreasing significance as estimated using the correlation t-test.

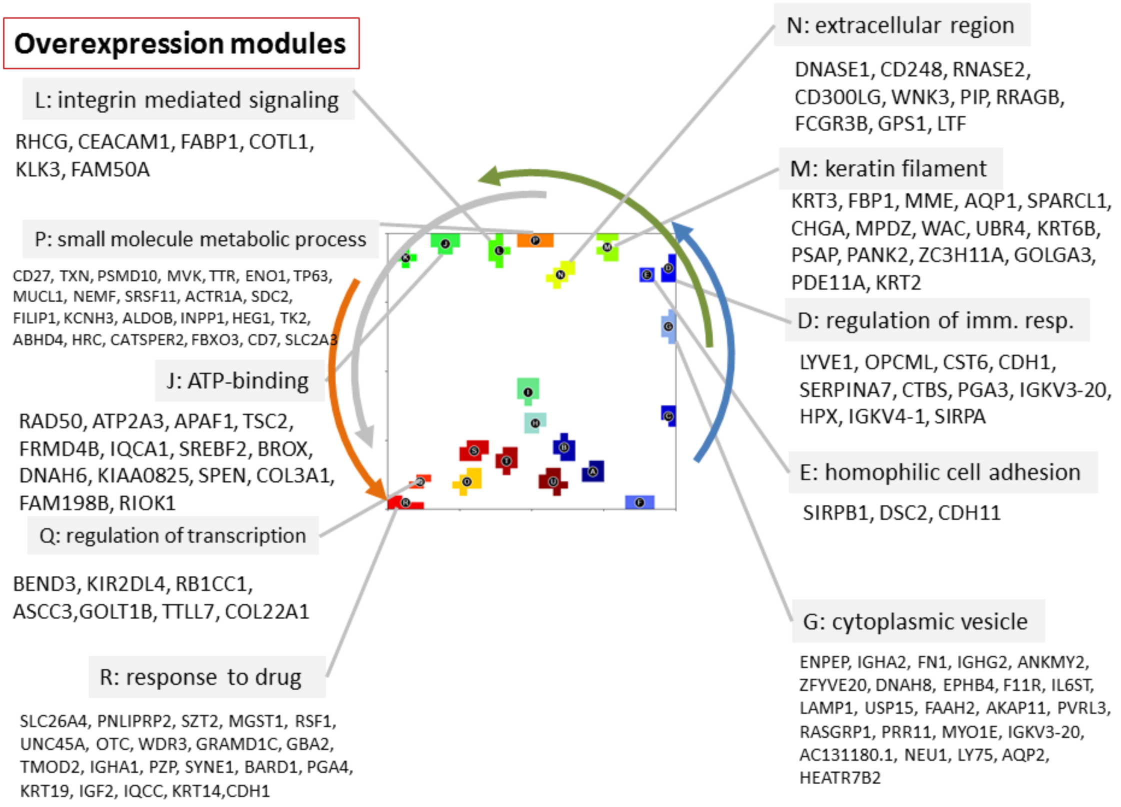

Figure S 10: Proteins in the overexpression spot modules.

2.7 Spot related pathway signal flows

For functional assignment of the spots we applied pathway flow analysis. Using the protein abundance data at each time point PFA provides pathway flow data for selected proteins in the overexpression spots. Note that in contrast to gene set enrichment analysis PFA uses the topology of selected pathways in combination with the protein data independent of the location of the proteins in the map. Spot assignment was obtained by correlating the pathway flow profiles with the mean meta-feature profiles of each spot and choosing the spot profile of maximum mutual correlation coefficient.

Figure S 11 shows the mean spot profiles (red) of selected spots together with the pathways showing strongest correlation of the PFA values of selected proteins (blue curves). Early and intermediate time range responses are associated with inflammatory processes, cell adhesion, ECM-receptor and antigen processing in qualitative agreement with the results of gene set enrichment analysis. Interestingly, metabolisms of nucleotides (purine and pyrimidine),of fatty acids (butanoate, pyruvate) and of amino acids (cysteine, metheonine and lysine) are also activated in this time range reflecting specific changes of the activity of liver and, partly kidney tissues in agreement with the results of tissue analysis (see below). Increased concentration of these metabolites in blood is characteristic for starvation when nucleotides and fatty acids are converted into glucose as evidenced by substrate balances across organs [5]. Intermediate time responses tentatively reflect regenerative (Wnt-pathway) and recombinant (N-glycan biosynthesis) processes [6]. Observed late time responses (p53- and mTOR-signaling pathway and ubiquinone biosynthesis) are indicators for cellular responses to different types of stress such as hypoxia and DNA damage, for nutrient and/or energy deficiency [7] and for changed energy metabolism.

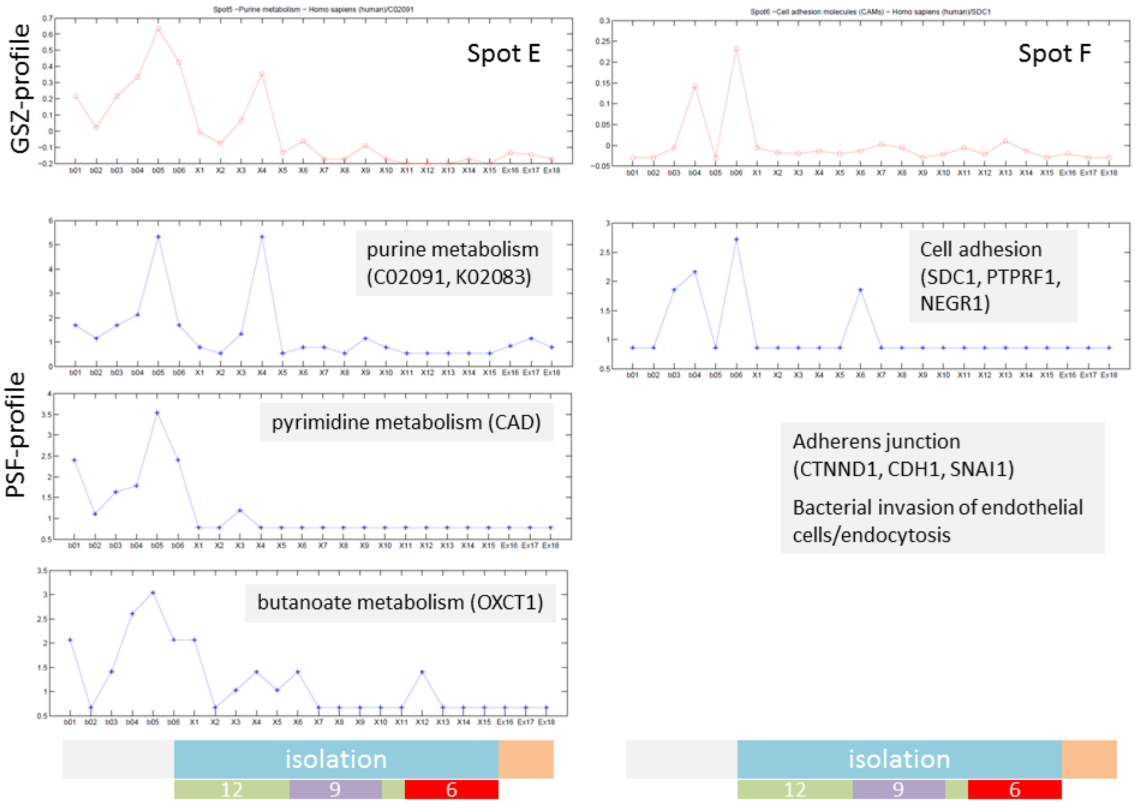
GSZ-profile
PSF-profile
Spot5 –Purine metabolism – Homo sapiens (human)/C02091
Spot E
purine metabolism (C02091, K02083)
pyrimidine metabolism (CAD)
butanoate metabolism (OXCT1)
Spot6 –Cell adhesion molecules (CAMs) – Homo sapiens (human)/SDC1
Spot F
Cell adhesion (SDC1, PTPRF1, NEGR1)
Adherens junction (CTNND1, CDH1, SNAI1)
Bacterial invasion of endothelial cells/endocytosis
isolation
12
9
6
isolation
12
9
6

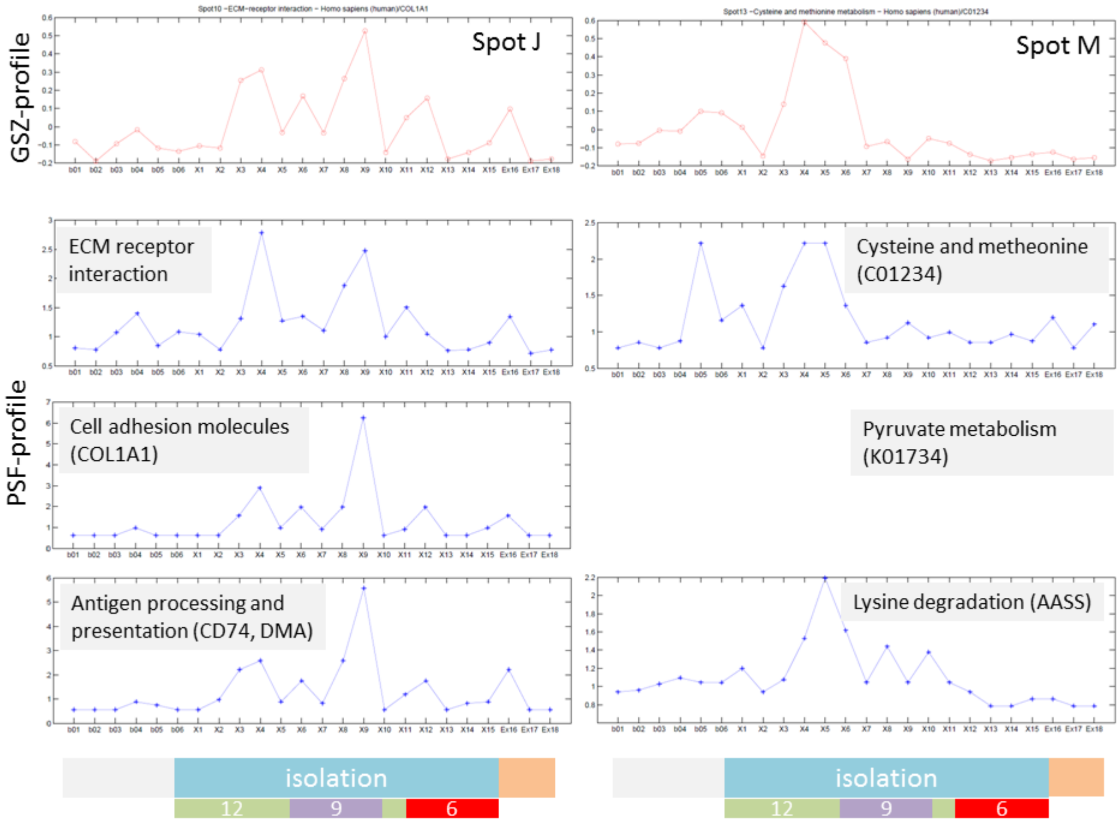
GSZ-profile
PSF-profile
Spot10 –ECM-receptor interaction – Homo sapiens (human)/COL1A1
Spot J
ECM receptor interaction
Cell adhesion molecules (COL1A1)
Antigen processing and presentation (CD74, DMA)
Spot13 –Cysteine and methionine metabolism – Homo sapiens (human)/C01234
Spot M
Cysteine and metheonine (C01234)
Pyruvate metabolism (K01734)
Lysine degradation (AASS)
isolation
12
9
6
isolation
12
9
6

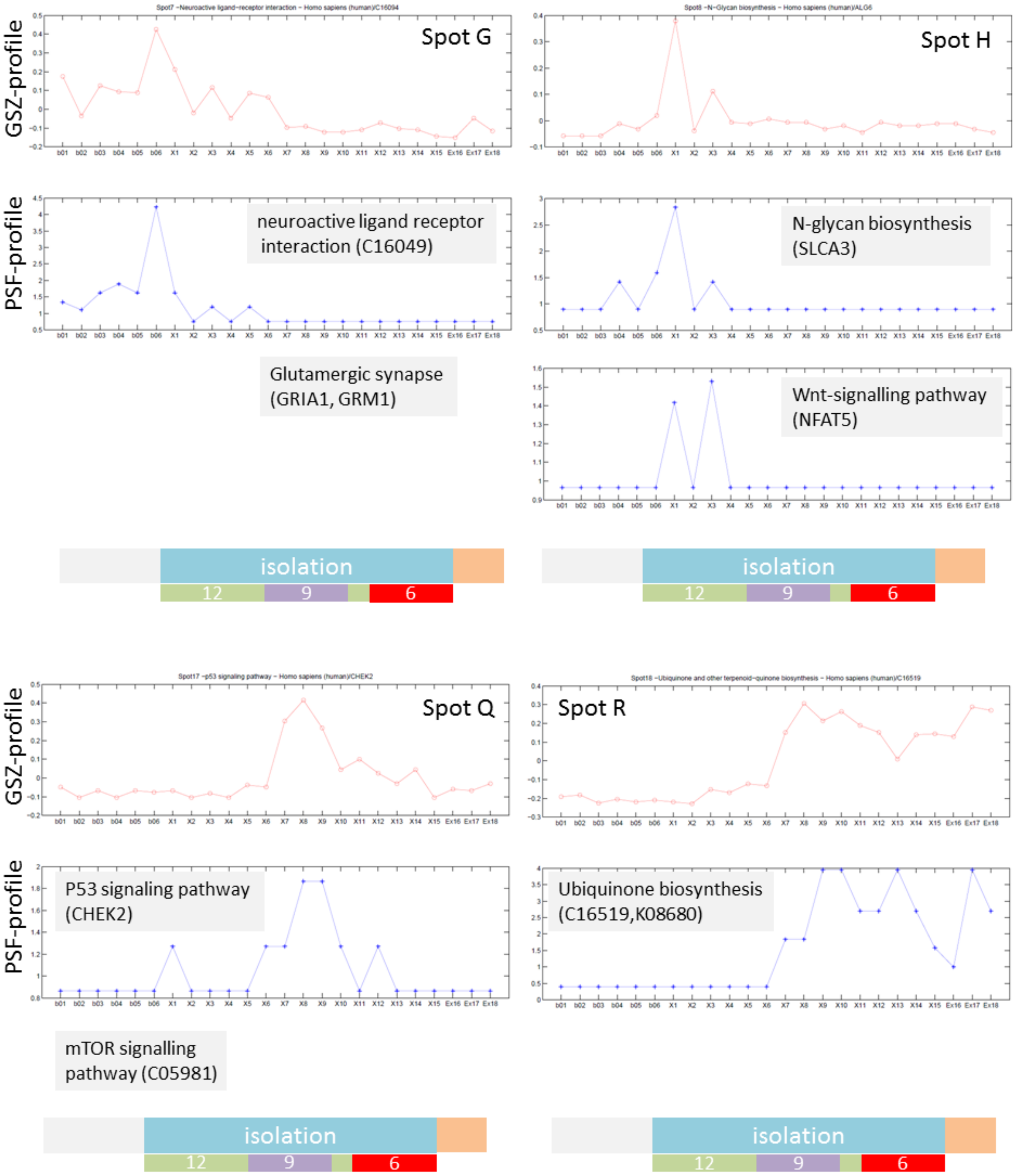

Figure S 11: Pathway flow analysis of selected overexpression spots referring to different time ranges. The mean expression profiles of the spots are shown in red whereas the respective PFA-profiles are shown in blue below together with the respective pathways and outcome-genes in parentheses.

2.8 Mapping of selected protein groups

We mapped groups of genes obtained in previous studies [8, 9] to our SOM space. The proteins collected in clusters 87, 83 and 9 (see [9] for assignments and details) accumulate in different areas in the early time region of the map (Figure S 12). Cluster 87 has been shown to associate with salt effects [9].

Figure S 12 shows the position of proteins commonly detected before and after space flight in urine samples of MIR cosmonauts *and* of the volunteers of the isolation experiment (group ‘constant’), detected in either the space flight or the isolation experiment (‘variable’) or detected only after space flight (‘flight specific’). The proteins are found in the regions of early and of late responses as well.

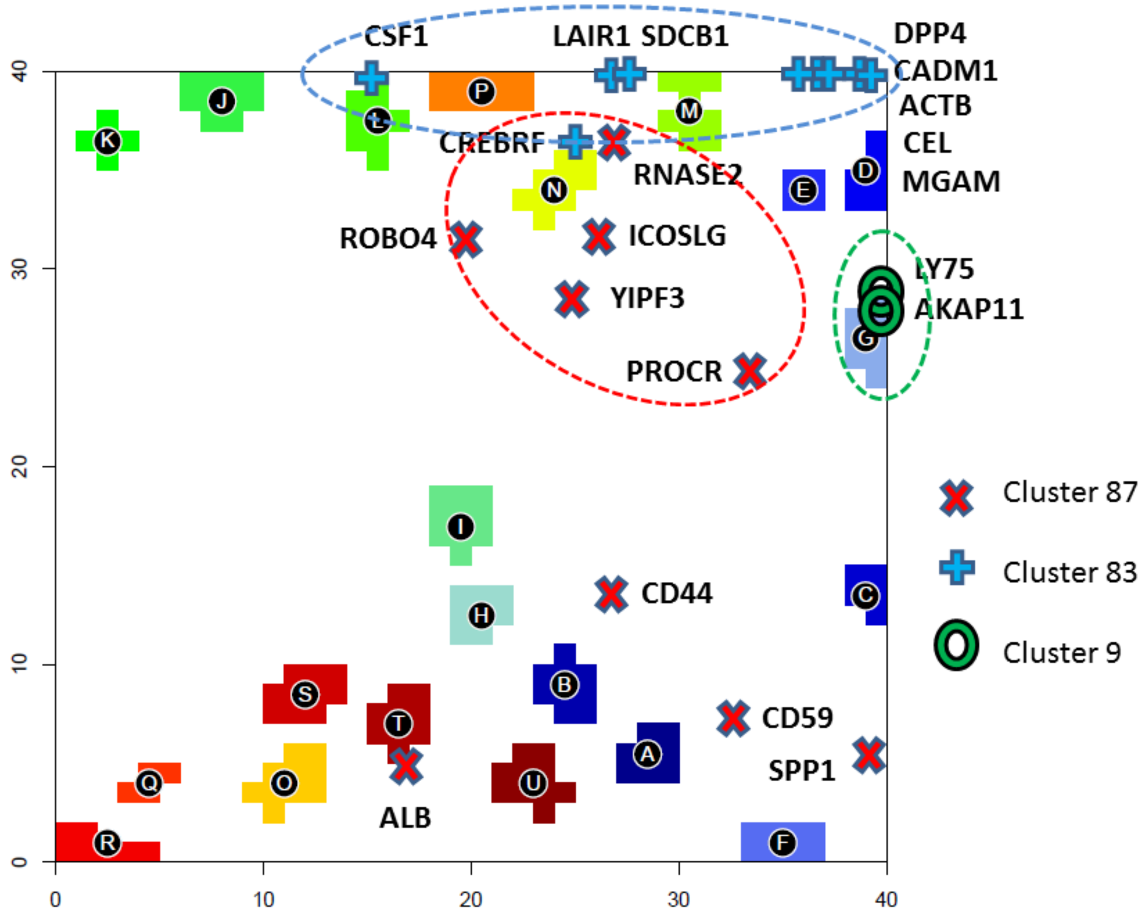

Figure S 12: Mapping of selected protein species from three clusters studied and defined previously [9]. The dashed ellipses indicate regions of increased local densities of the proteins from the three clusters. Cluster 87 is associated with salt effects.

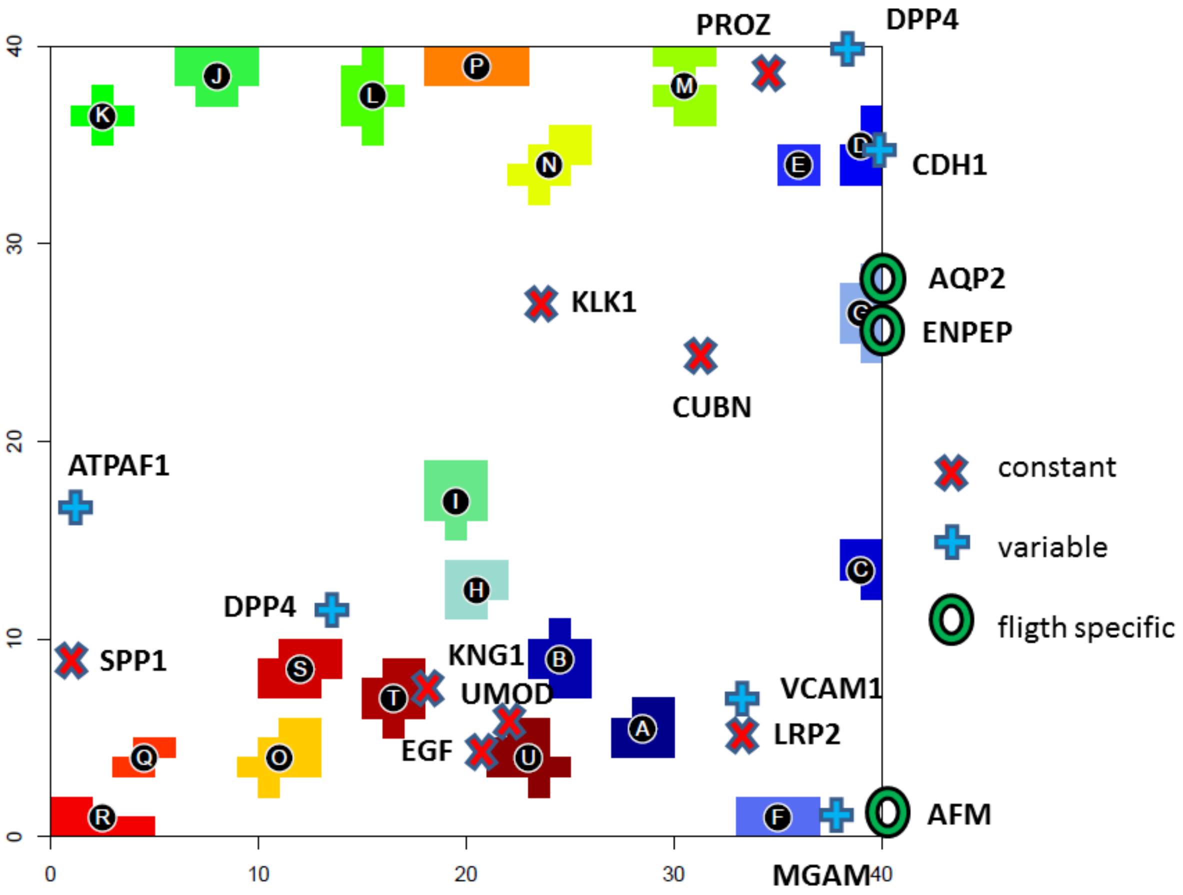

Figure S 13: Mapping of selected proteins to SOM: Constantly present under physiological conditions and after flight (symbol x), variably present (symbol +) and specifically expressed in urine samples of cosmonauts after flight (symbol O). Data are taken form ref. [8]. Most of the ‘constant’ and ‘variable’ proteins belong to the single spiked and rare spot areas whereas the flight specific accumulate in the early_up region, meaning that these species are down regulated before and after isolation experiment.

2.9 Single volunteer analysis

The spot textures of the individual volunteer SOM cannot be directly compared to that of the mean volunteer analysis because both SOM are trained independently. One gets however an analogous number of about 9 – 10 overexpression spot clusters with continuous profiles containing 12 – 61 features per spot. These profiles reflect the essential properties of protein kinetics for each of the probands as observed also in the mean volunteer SOM (Figure S 14). Detailed inspection of these profiles reveals for example, that, the late regime spot characteristics of P5 in the measurements before isolation is due to a slightly reduced overall level of the abundance of the respective proteins compared with the other probands but not to a different time course (see spots H, P and Y in Figure S 14).

In addition, our algorithm detects about 30 spots of the single spiked and rare type (Figure S 15): Each of these profiles shows at minimum one spiked protein expression and contains about 20 single proteins. These spots are insignificant in terms of correlated sets of proteins ($-\log_{10}p(\text{beta})>0.1$). Moreover, each of them was found in less than 5% of the samples and contains usually less than 20 features per spot. They show typically strong positive, spike-like outliers in one or a very few samples only. We attribute these spots tentatively, to technical errors of the measurement and/or to very specific physiological effects of unknown origin. These spots were excluded from further discussion because of their singular character. Importantly, the SOM sorting algorithm reliably separates such features from the features responding continuously to the experimental conditions. The identification of such spiked profiles would allow us to study the origin of this effect more in detail and also to develop and to apply suited correction methods. These issues are however beyond the scope of this publication.

Figure S 16 shows the gene set enrichment heatmap of the single volunteer analysis. Most of the enriched processes ‘switch’ in a coordinated fashion in the different volunteers. One sees however also individual differences, e.g. the volunteers show different degrees of immune response during the experiment.

An alternative approach to extract single volunteer information is illustrated in Figure S 17: It shows the so-called profiling map which is obtained by training of a coarse grained SOM of size 10x10. Each tile of the map compares the profiles of the individual probands referring to the respective meta features. The profiles roughly divide into early_up, intermediate_up and late_up types which show an almost concerted expression among the probands. A fourth group of profiles indcates stronger individual differences in different time ranges of the experiment. Owing to the smaller number of meta features the individual profiles are less resolved as in the larger 40x40 standard map. Single and rare profiles mostly collect in the range of ‘individual’ profiles.

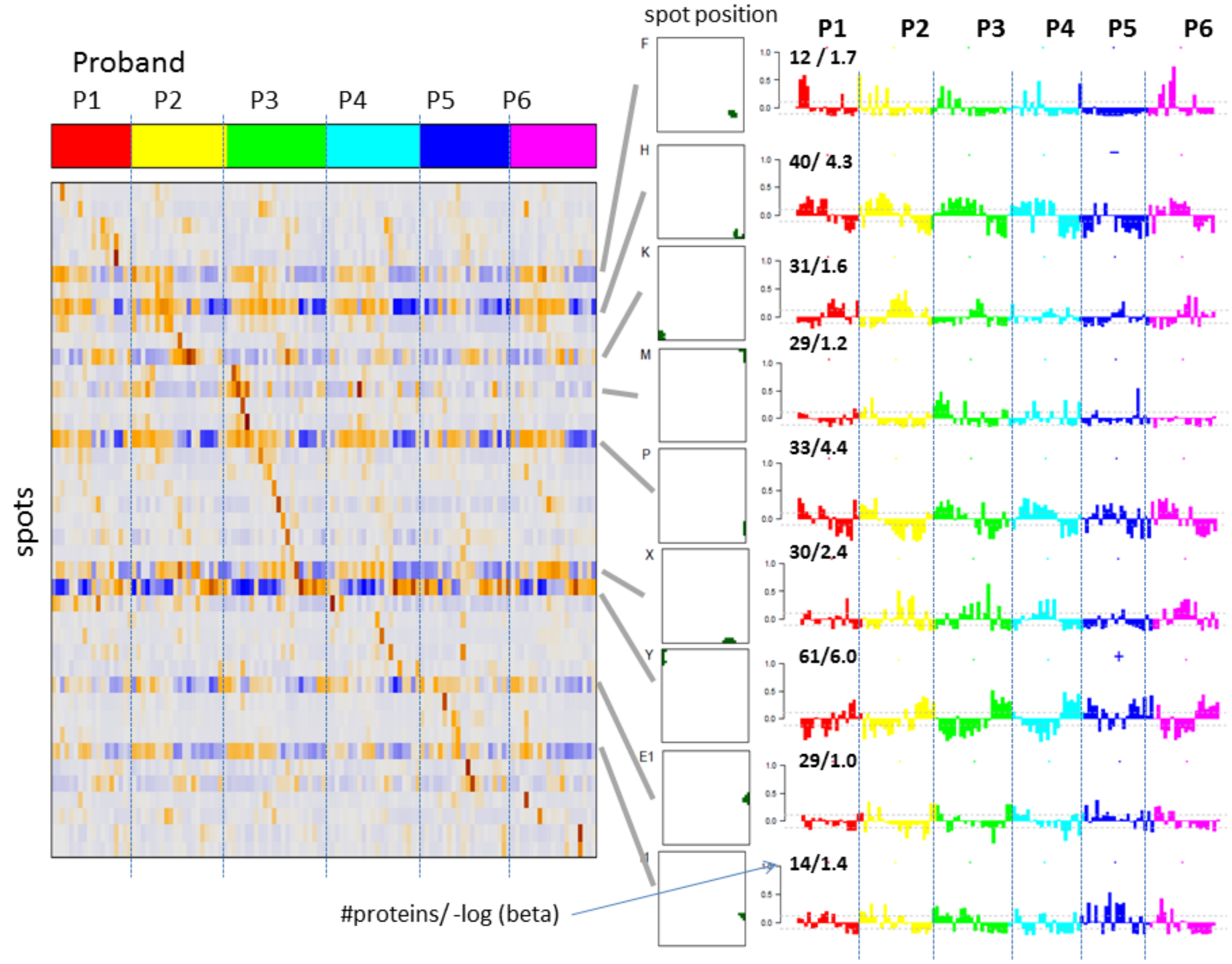

Figure S 14: Protein abundance profiles of overexpression spot clusters. The heat map provides an overview over the abundance profiles (dark brown to blue indicate high to low abundance levels, respectively). The right part selects profiles showing continuous responses, i.e. not referring to so-called single-spiked or rare profiles (these data are shown in the supplementary text). The spiked profiles can be identified in the heat map: They are characterized by single-brown colored bars due to high abundance levels of single or only a few protein species at single time points only (see also the supplementary text for full profiles). Note that the profiles of proband no. 5 (P5) reveal either low (spots H and P) or high (spot Y) abundance levels in the early time range compared with the respective spot profiles of the other probands which causes the deviating abundance portraits (see the main paper) and course of the sample trajectory of P5. On the other hand, the shapes of the profiles of P5 in general agree with that of the other probands showing that the specifics of P5 refer rather to absolute protein abundance levels and not to relative changes during the experiment.

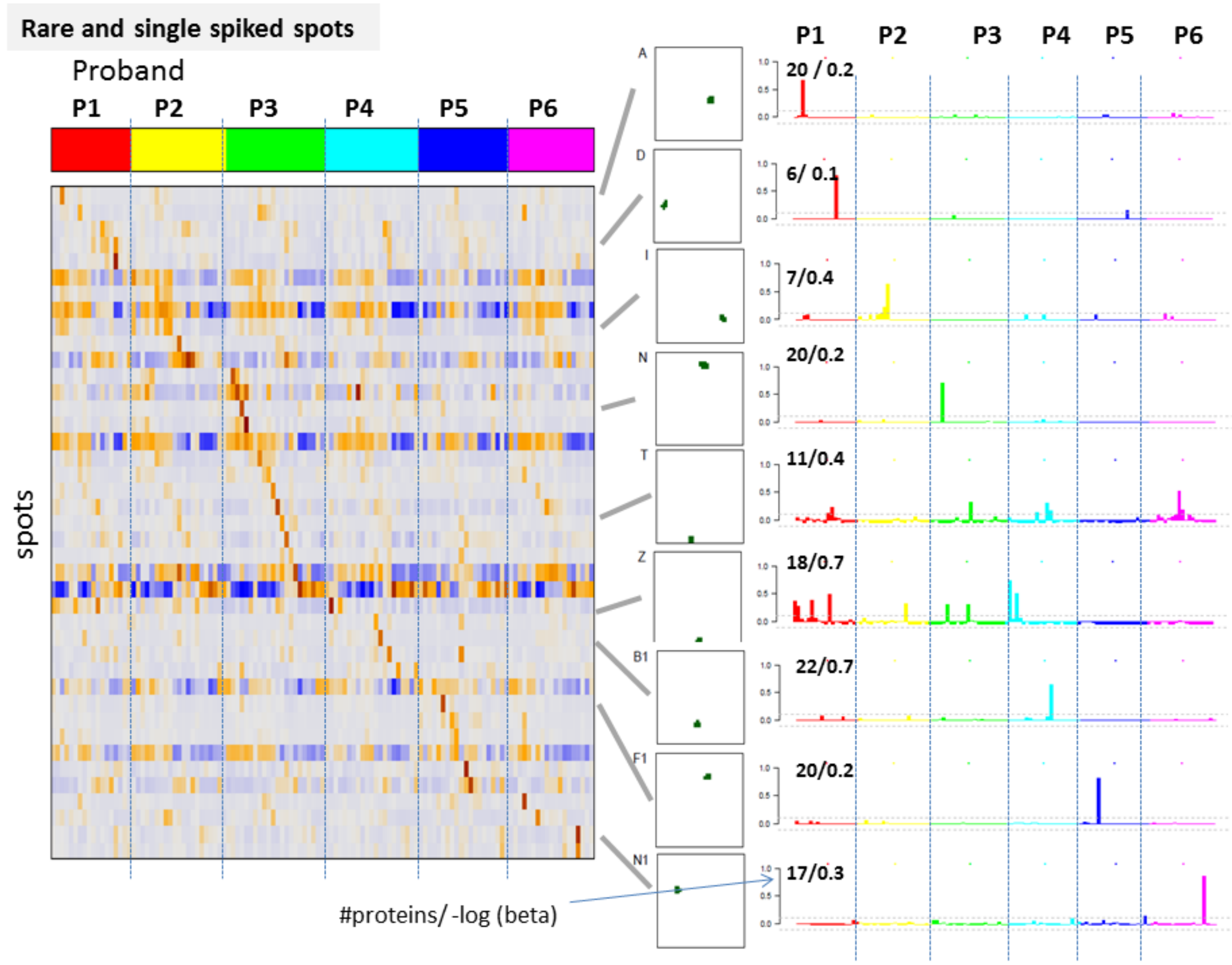

Figure S 15: Protein expression profiles of overexpression spot clusters. The heat map provides an overview over the expression profiles (dark brown to blue indicate high to low expression levels, respectively). The right part selects profiles referring to so-called single-spiked or rare profiles.

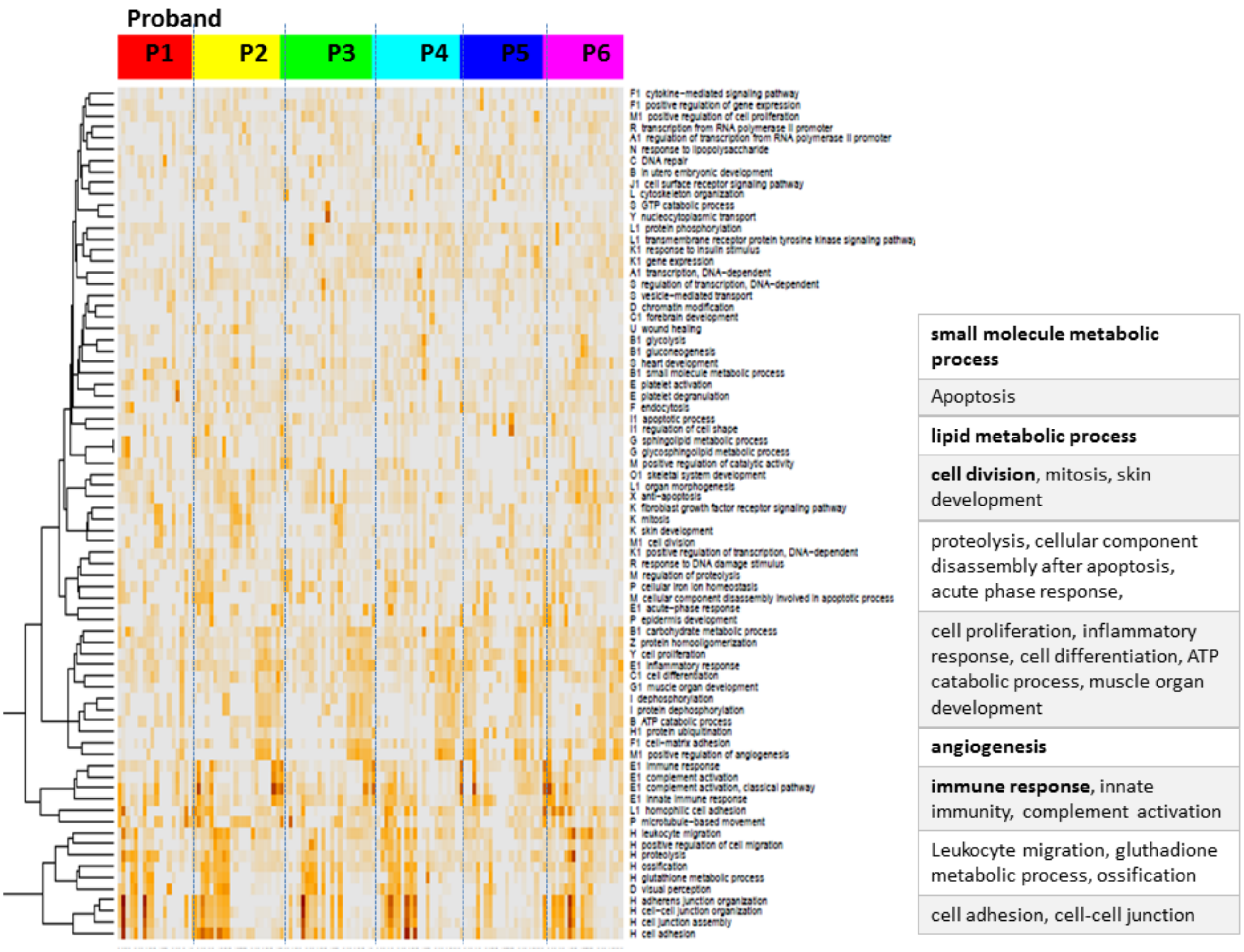

Figure S 16: Global enrichment analysis heat map of the single volunteer analysis: The map clusters top GO-sets of the category 'biological process' enriched in overexpression spots of the time series. Key processes are listed in the right part of the figure. Brown to grey indicates high-to-low enrichment estimated using the GSZ-score.

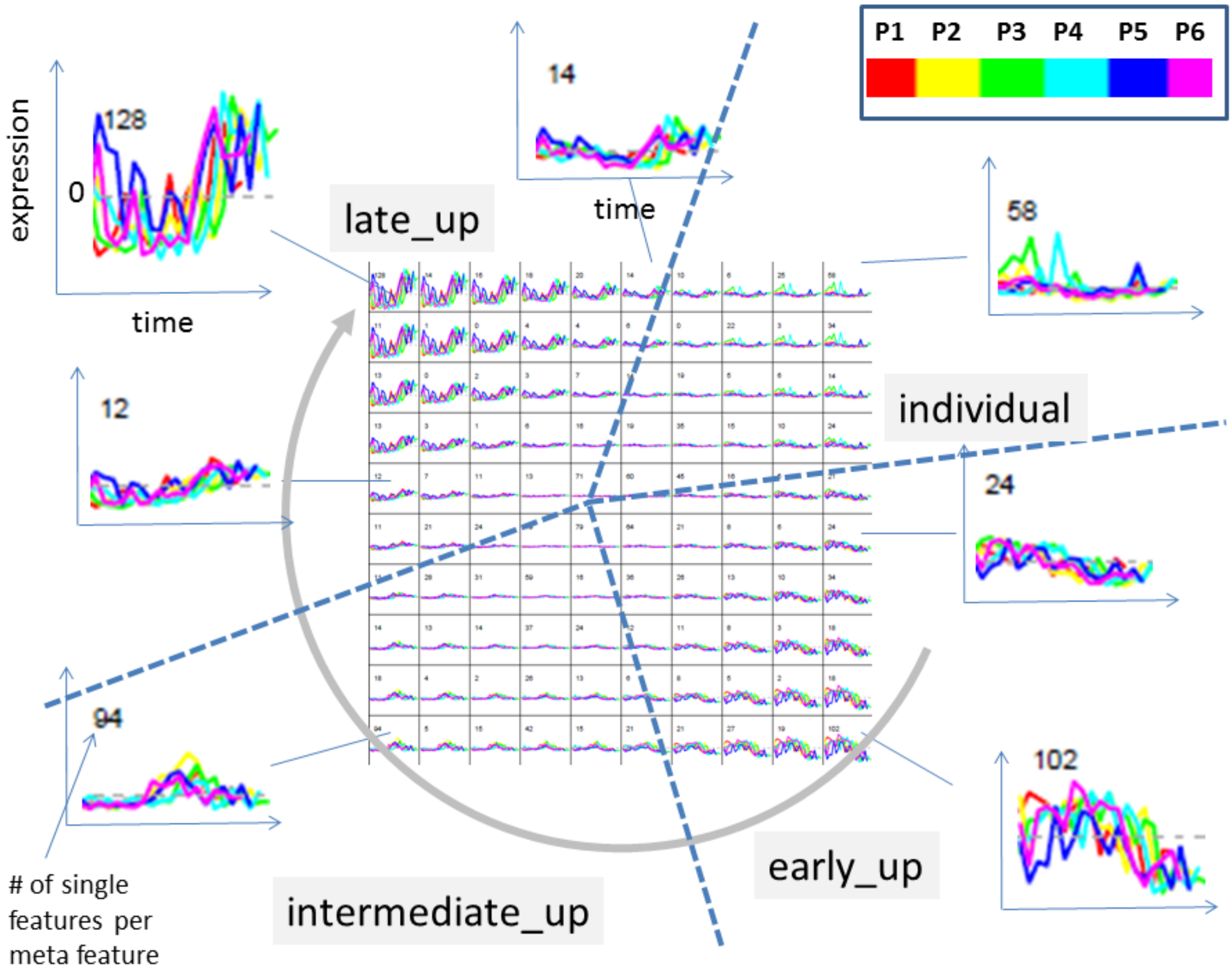

Figure S 17: Meta-feature profile map of single volunteer analysis: A coarsely resolved 10x10 SOM was trained with single volunteer data. The profile map compares the time profiles of the probands P1 – P6 (see legend for assignment) in each of the meta feature tiles. Profiles divide into early_up, intermediate_up, late_up and 'individual' profiles. Profiles of selected tiles are enlarged.

2.10 Organ related protein expression

We analyzed selected tissue-related sets of proteins as described in the main paper. Figure S 18 shows the results for a series of tissues which can be roughly divided into early responders (pancreas, liver, kidney), intermediate (muscle) and late (testis, stomach) responders and into tissues weakly or not responding to the experiment (skin, lymph node, blood, prostate, brain, colon). The type of response is clearly documented in the respective GSZ-profiles which plot the gene set enrichment score of the set as a function of time. Here all protein expression values of the set are considered, compared with the mean expression of all proteins considered and normalized using the variance of the expression values of the set:

$$GSZ_{set,t} = \frac{\left\langle E_{p,t} \right\rangle_{p \in set} - \left\langle E_{p,t} \right\rangle_{all\ p}}{\sqrt{\operatorname{var}\left(E_{p,t}\right)_{p \in set} / n_{set}}} \quad . \tag{12}$$

The GSZ-value thus estimates the consistency of differential expression of the set members compared with the mean expression of all genes in a given state. The GSZ consequently characterizes the expression of the whole protein set.

In addition we show so-called protein set population maps which mark the positions of the protein species of each set in the average volunteer SOM. Accumulation of the proteins in regions assigned to a certain time range (see red rectangles) indicates that the set is affected by the experiment.

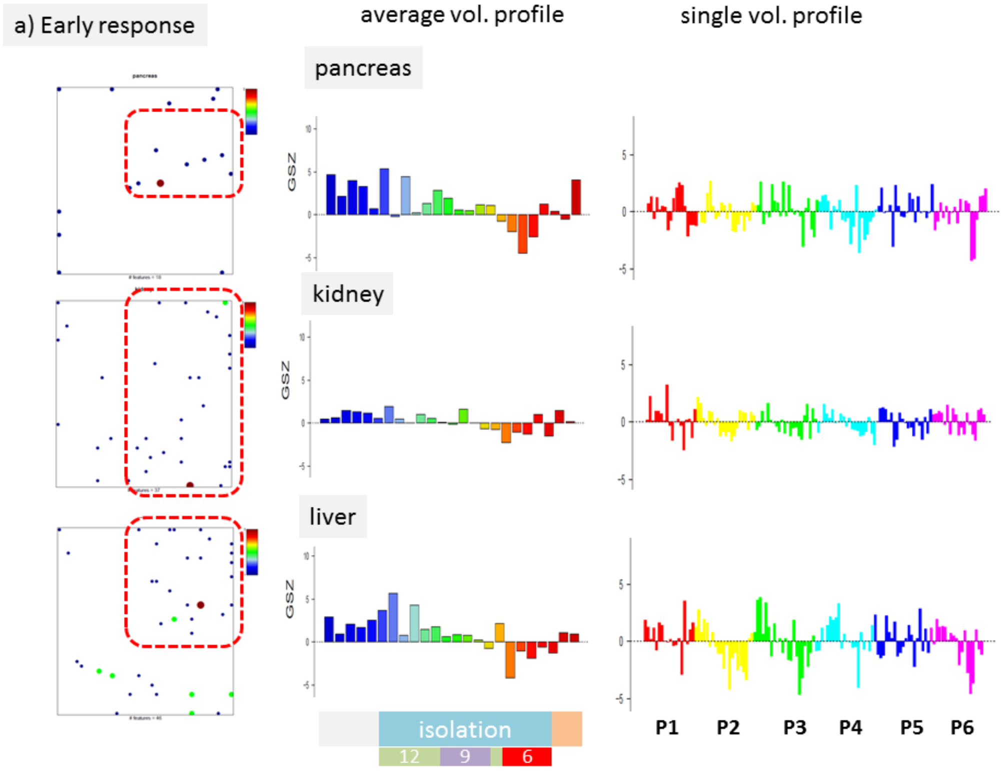

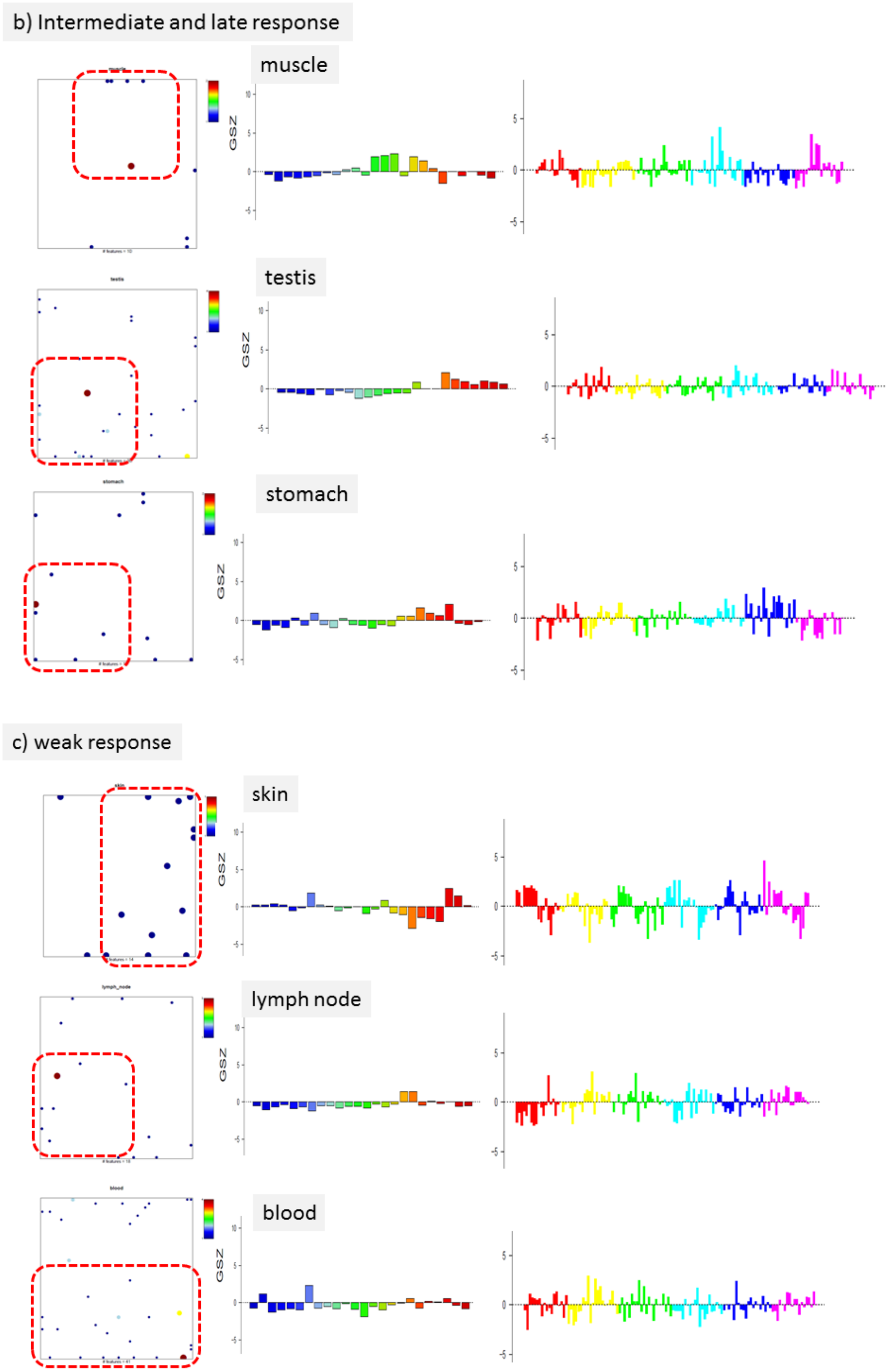
b) Intermediate and late response
muscle
GSZ
testis
GSZ
stomach
GSZ
c) weak response
skin
GSZ
lymph node
GSZ
blood
GSZ

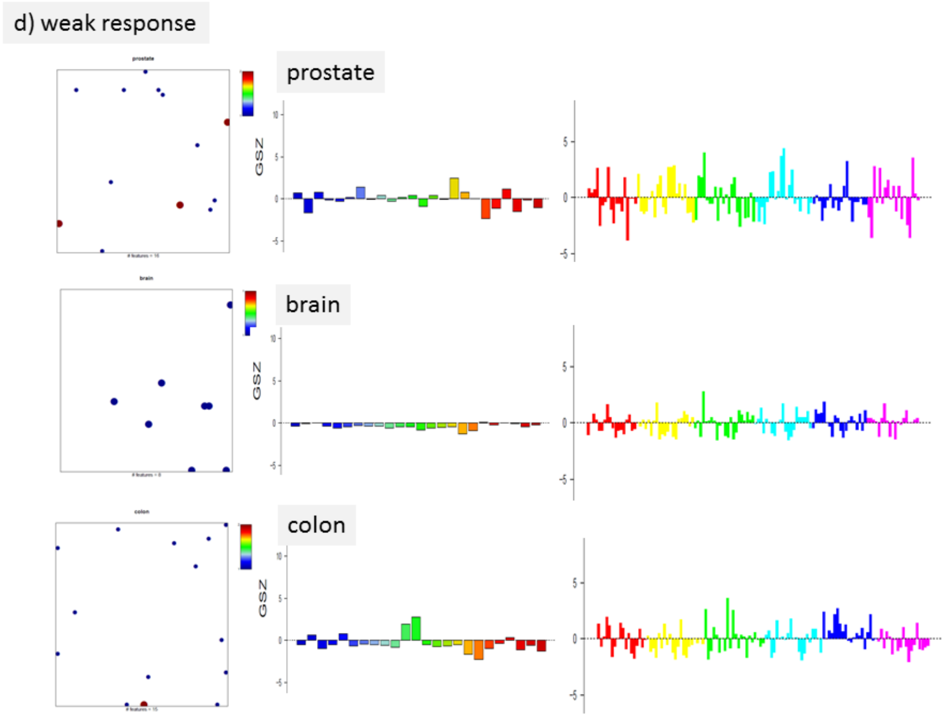

Figure S 18: Tissue specific protein expression: Tissue specific protein sets are taken from TiGR[10] and mapped into the single volunteer map (left part). The red rectangles illustrate regions of increased local density of the respective proteins. These regions refer to different time ranges. The set-profiles shown in the middle part clearly reveal the different time profiles in the average volunteer analysis. The respective single volunteer analysis reflects similarities and proband-specific differences between their tissue expressions.

2.11 Mapping and profiling of selected GO protein sets

Below in Figure S 19 selected protein sets are mapped into the SOM map of average volunteer analysis using the same presentation as for the organ related protein expression in Figure S 18. The bar plots show their average and volunteer specific profiles. The plots support the results obtained using spot analysis and gene set enrichment heat maps shown above.

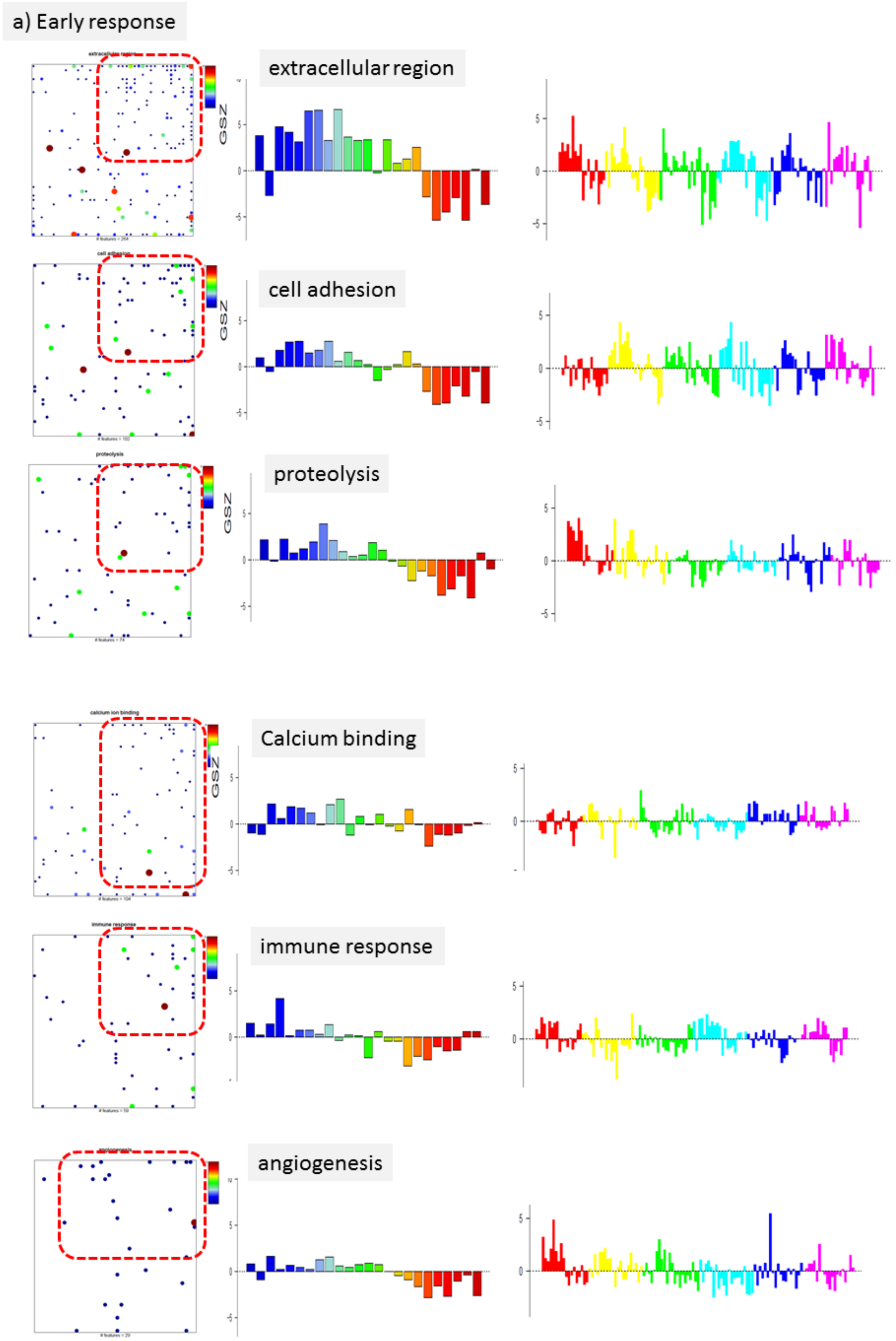

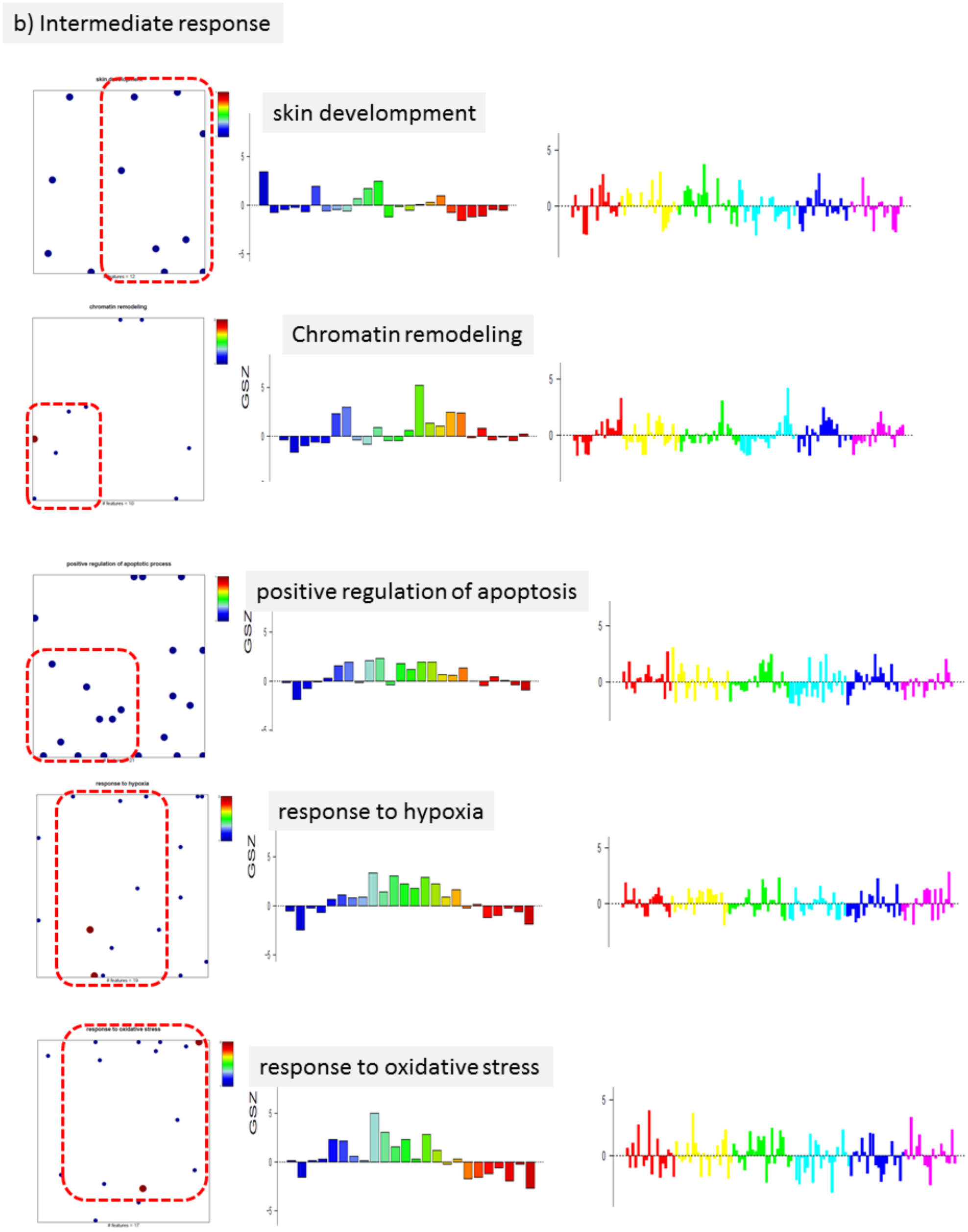

b) Intermediate response
skin develompment
Chromatin remodeling
positive regulation of apoptosis
response to hypoxia
response to oxidative stress
GSZ

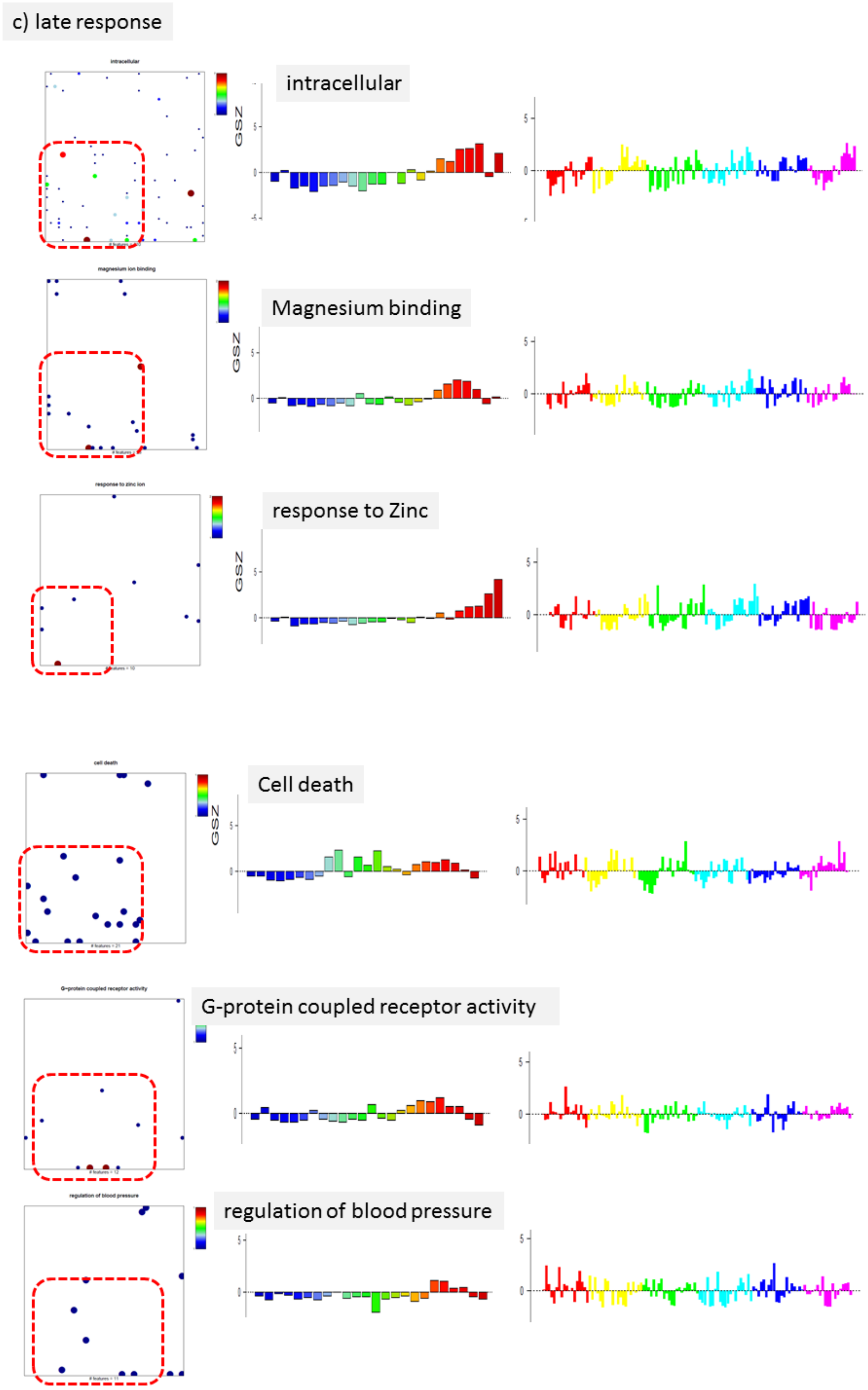

Figure S 19: Mapping and profiling of selected GO gene sets into the single volunteer map (left part). The red rectangles illustrate regions of increased local density of the respective proteins. These regions refer to different time ranges. The set-profiles shown in the middle part clearly reveal the different time profiles in the average volunteer analysis. The respective single volunteer analysis reflects similarities and proband-specific differences between the mean expression of the sets selected.

2.12 SOM analysis of absolute protein expression levels

We trained a SOM with total expression values E_{pt} instead of ΔE_{pt} (see Eq. (1)). This way, absolute levels of protein expression were taken into account. Recall that the expression is defined as the mean detection call of the respective proteins in all six probands. It adopts a value between 0 and 1 defining the fraction of present calls obtained. It means for example that for a value of unity all proteins are detected in all probands. The obtained SOM portraits reveal less structured expression landscapes compared with the SOM of centralized values showing essentially only one global expression maximum and one expression minimum spot in red and blue, respectively. Their position only weakly shifts in the course of the experiment (see the portraits in Figure S 20). These spots obviously collect permanently high and low expressed proteins, respectively. The question arises whether these landscapes contain similar information about the differential expression of groups of proteins detected at early, intermediate and late times of the experiment by means of the SOM analyses performed so far. With this aim we generated the 2nd level SOM of absolute expression landscapes. It closely resembles the respective plot of the centralized data (Figure S 18a). The sample trajectory clearly divides into the three time ranges revealing this way that the individual portraits contain the full information in this respect. The spot trajectory (Figure S 20b) can be assigned to the time ranges discussed so far. In addition to the permanently up and down profiles a 'late down' one is identified (see the detailed profiles shown in Figure S 20 below).

In the next step we used the supporting variance and entropy maps in combination with K-means spot clustering to segment the map into different modules of co-expressed proteins (Figure S 21):

a) The area of low variance and low entropy can be attributed to permanently low expressed proteins. About 63% of all proteins show this kind of behavior.

b) The area of high variance and high entropy collects proteins permanently highly expressed (7%).

c) The area of high variance and high entropy refers to proteins highly expressed in the early and intermediate time ranges only (late down, 13%).

d) Profiles of proteins up-regulated in the intermediate time range only possess medium variance and entropy values (7%). These medium values indicate that the high expression states are observed only at a few time points giving rise to relatively sharp peaks in the profiles.

e) Late_up-regulated proteins collect in a second area of high variance and high entropy (7%) which is however well separated from area b).

Each of these areas splits into several K-means cluster modules of slightly different profiles (see Figure S 21 and Figure S 22). Their inspection reveals a continuum of different shapes which partly cannot be clearly assigned to one of the groups defined above. Instead, they occupy a sort of intermediate position between them. We therefore used the K-means cluster spots for functional analysis using protein set enrichment. We used 'area-filling' K-means clustering because we aim at taking into account all proteins.

Detailed inspection of the spot modules in Figure S 20 shows that a group of about 65 proteins with an inflammatory signature are permanently expressed over the whole period of the experiment. The profiles of the remaining spots of module b) and especially of module c) decrease more or less sharply in the late time range. These modules thus contain the proteins which deplete at low NaCl consumption in the late phase of isolation. Interestingly, part of the spot profiles express onerelative sharp peak in the early time range (spot F and D) or a second one in the intermediate one (e.g. spots M, L, B and F). This second peak becomes more pronounced in intermediate-time mode d). The third peak protrudes already in a few spots of this mode but it becomes much more intense in the late mode e) together with the fourth peak near the end of the experiment. Hence, the more or less constant or decreasing profiles in modes b) and c) overlay with peaked profiles with maxima at distinct positions (as indicated by the asterisks in the figure). Functional analysis essentially supports the results of the previous analysis using centralized expression profiles.

In summary, absolute expression analysis shows that a series of processes become activated in relatively narrow time windows at four fixed times during the experiment, namely at or immediately before isolation (angionesis, complement activation and others), at or immediately after reducing salt consumption to 9 g/day (focal adhesion and cytoskeleton) and to 6 g/day (cell differentiation and organ development) and near the end of the experiment after isolation. The latter trend suggests recovery of the initial state before starting isolation. Double peaked profiles combine peaks at late and intermediate times (e.g. metabolic process and apoptosis).Importantly, immune response processes are permanently active during the experiment with a slight decay in the late time range.

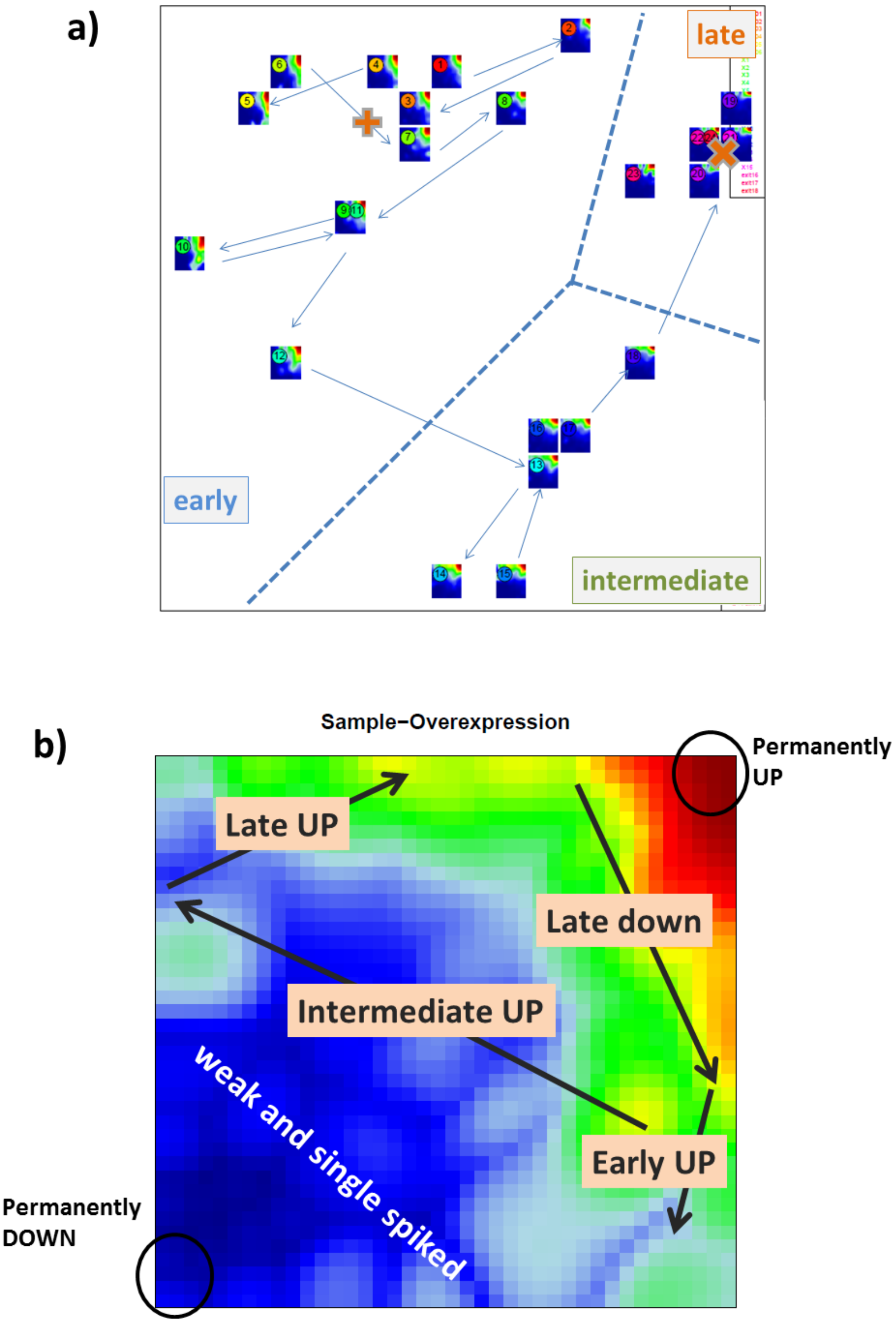

Figure S 20: Sample and spot trajectories of absolute expression SOM analysis: a) 2nd level SOM mapping of the total expression data of the ‘averaged volunteer’. The trajectory divides into an early, intermediate and late regime in agreement with the respective results obtained from centralized data. Note that the portrays (see the small images) are much less structured showing only one red spot compared with the portraits obtained using centralized data. b) Overexpression summary map of the SOM images: The red and blue spots refer to permanently present and almost absent proteins, respectively.

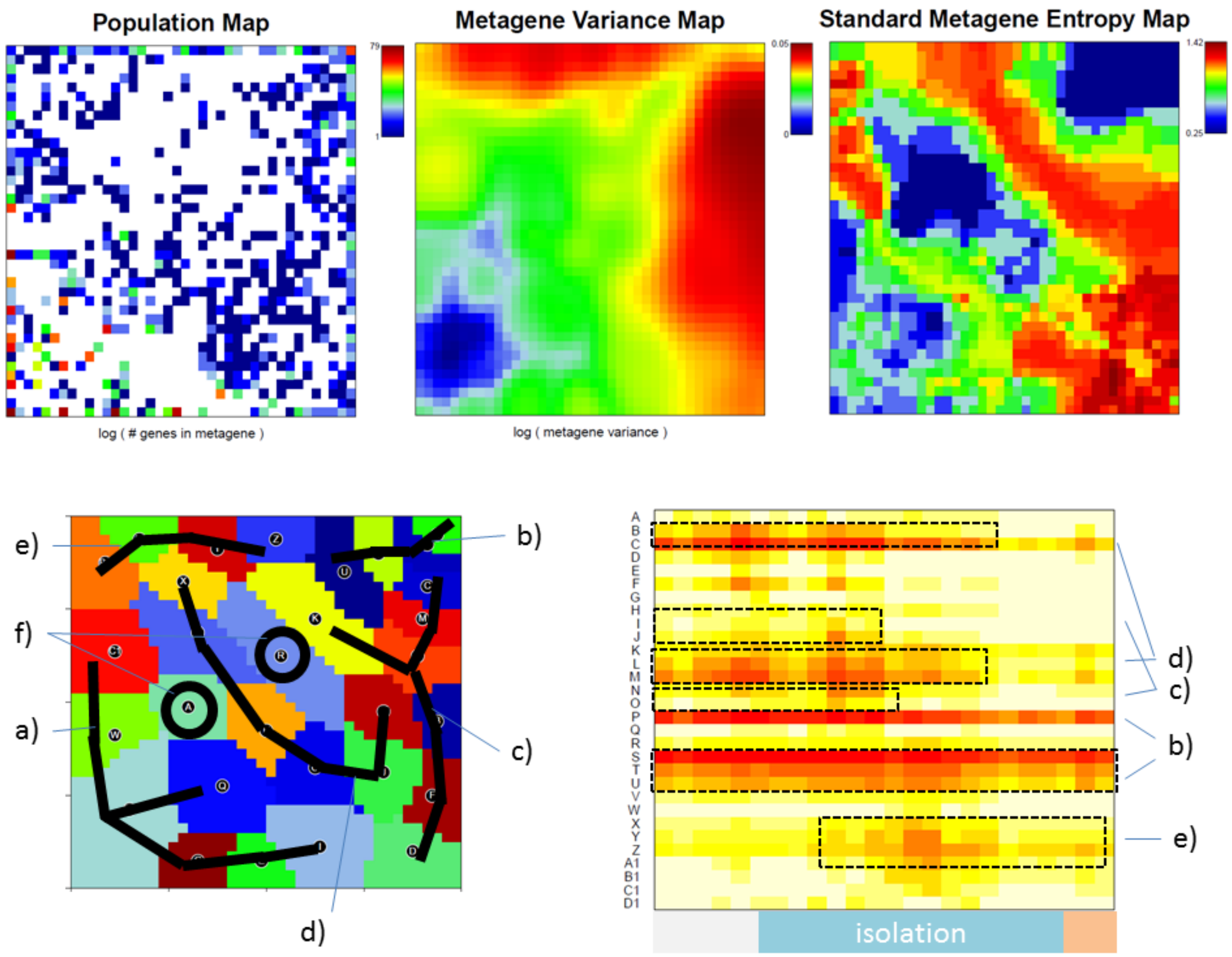

profiles	variance	entropy	#proteins	%proteins
a) low and single spiked	L	L	1302	64%
b) high	H	L	145	7%
c) early_up	H	H	267	13%
d) inter_up	M	M	147	7%
e) late_up	H	H	137	7%
f) diverse	M	L	40	2%

H, M, L .... high, medium and low levels, respectively

Figure S 21: Supporting maps (first row of figures) used to segment the K-means spot map (second row) into six absolute expression modes as indicated by the black curves connecting the spots of each mode. The heat map shows the expression level of each spot (red refers to high, white to low expression). The table assigns the characteristic variance and entropy levels to the modes and provides the number and fraction of proteins per mode.

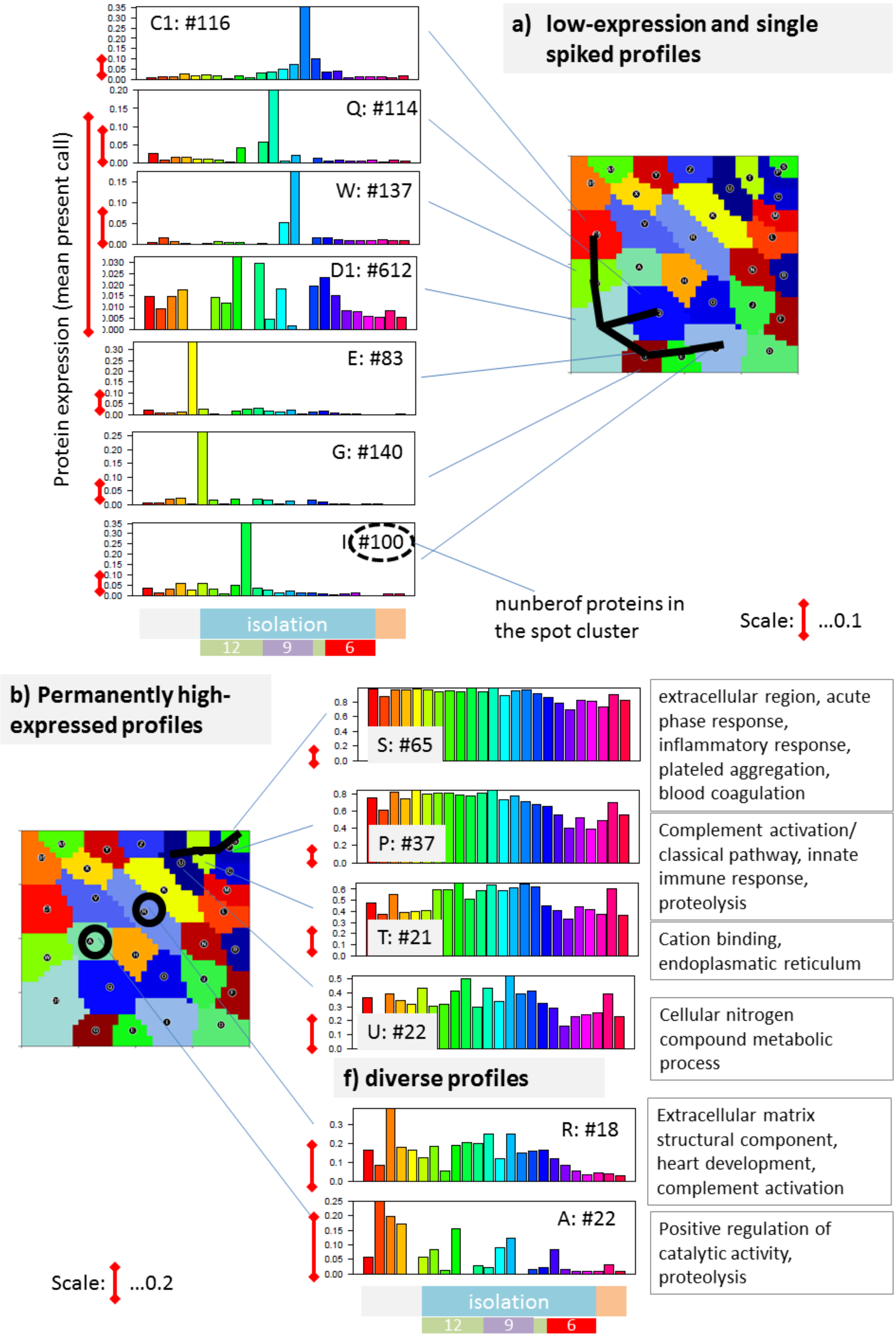
a) low-expression and single spiked profiles
Protein expression (mean present call)
C1: #116
Q: #114
W: #137
D1: #612
E: #83
G: #140
I #100
isolation
12
9
6
nunberof proteins in the spot cluster
Scale: ...0.1
b) Permanently high-expressed profiles
S: #65
P: #37
T: #21
U: #22
extracellular region, acute phase response, inflammatory response, plateled aggregation, blood coagulation
Complement activation/ classical pathway, innate immune response, proteolysis
Cation binding, endoplasmatic reticulum
Cellular nitrogen compound metabolic process
f) diverse profiles
R: #18
A: #22
Extracellular matrix structural component, heart development, complement activation
Positive regulation of catalytic activity, proteolysis
Scale: ...0.2

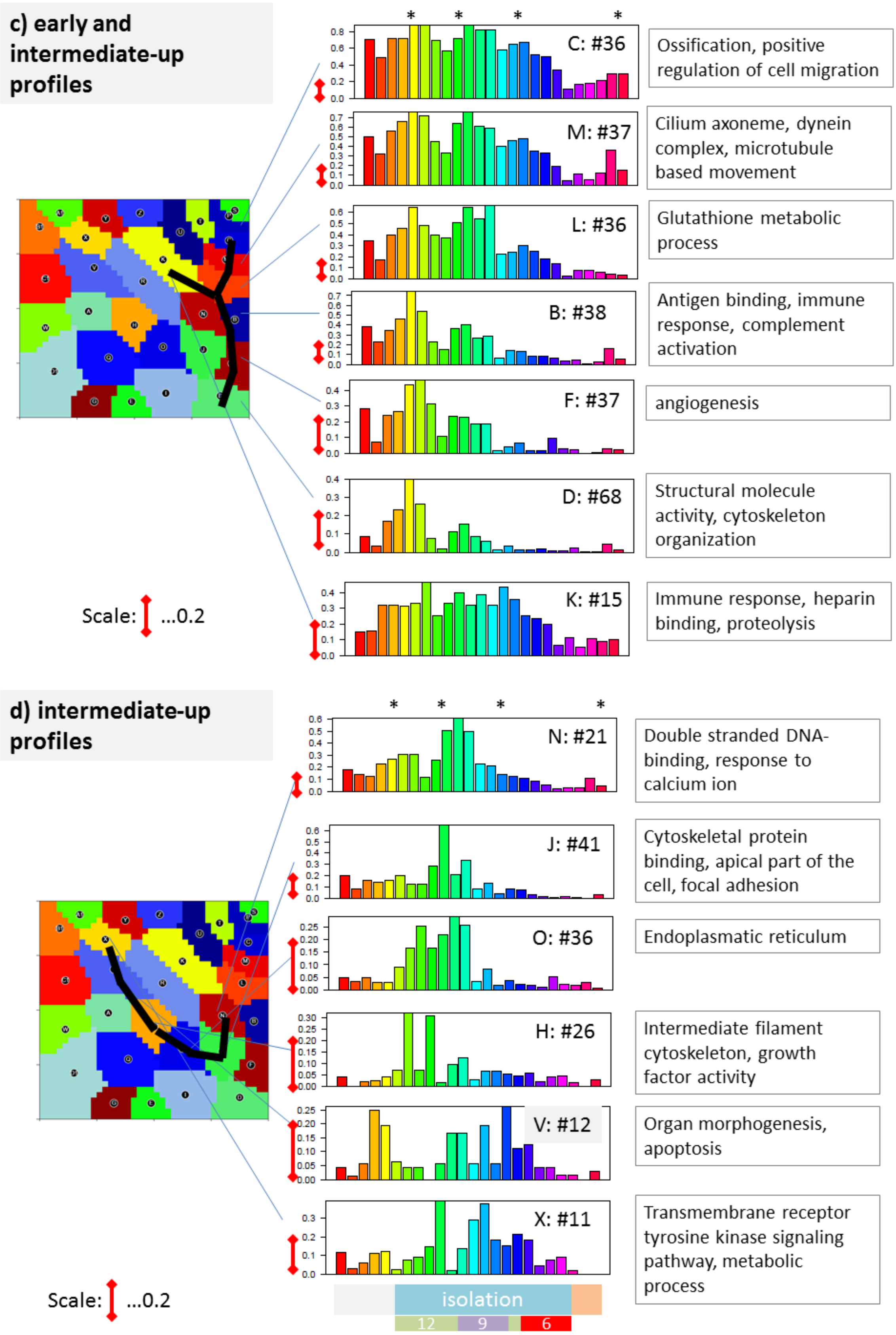
c) early and intermediate-up profiles
C: #36
Ossification, positive regulation of cell migration
M: #37
Cilium axoneme, dynein complex, microtubule based movement
L: #36
Glutathione metabolic process
B: #38
Antigen binding, immune response, complement activation
F: #37
angiogenesis
D: #68
Structural molecule activity, cytoskeleton organization
K: #15
Immune response, heparin binding, proteolysis
Scale: ...0.2
d) intermediate-up profiles
N: #21
Double stranded DNA-binding, response to calcium ion
J: #41
Cytoskeletal protein binding, apical part of the cell, focal adhesion
O: #36
Endoplasmatic reticulum
H: #26
Intermediate filament cytoskeleton, growth factor activity
V: #12
Organ morphogenesis, apoptosis
X: #11
Transmembrane receptor tyrosine kinase signaling pathway, metabolic process
isolation
12
9
6
Scale: ...0.2

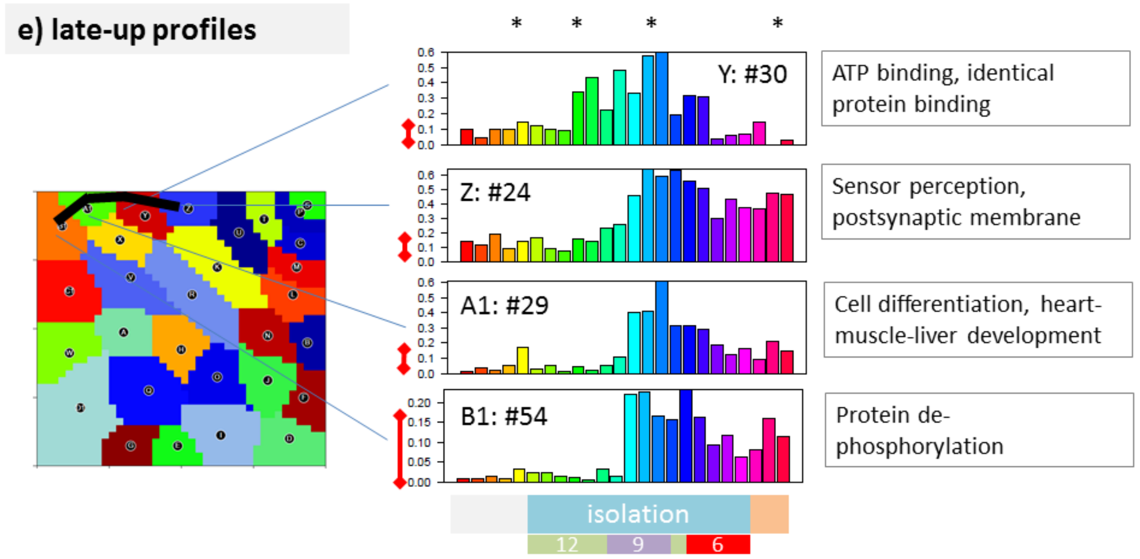

Figure S 22: Expression profiles of the K-means spot clusters of each of the modes a)-f). Enriched protein sets from the GO-terms biological functions, cellular component and molecular function in each of the spots are listed in the boxes in the right part. The number of proteins per spot is given as #protein_number. The asterisks indicate the peak positions observed also in the overall profiles (see main paper). Note that the scale of vertical expression axis changes from plot to plot. The vertical red dumbbell scales an expression value of 0.1 (module a only) or 0.2. Full protein lists of each of the spot modules are provided in additional file 3.

3 References

1. Läuter J, Glimm E, Eszlinger M: **Search for relevant sets of variables in a high-dimensional setup keeping the familywise error rate**. *Statistica Neerlandica* 2005, **59**:298-312.

2. Läuter J, Horn F, Rosolowski M, Glimm E: **High-dimensional data analysis: Selection of variables, data compression and graphics - Application to gene expression**. *Biometrical Journal* 2009, **51**(2):235-251.

3. Arakelyan A, Nersisyan L: **KEGGParser: parsing and editing KEGG pathway maps in Matlab**. *Bioinformatics* 2013, **29**(4):518-519.

4. Wirth H, Loeffler M, von Bergen M, Binder H: **Expression cartography of human tissues using self organizing maps**. *BMC Bioinformatics* 2011, **12**:306.

5. Owen OE, Felig P, Morgan AP, Wahren J, Cahill GF, Jr.: **Liver and kidney metabolism during prolonged starvation**. *The Journal of Clinical Investigation* 1969, **48**(3):574-583.

6. Pfeiffer G, Strube K-H, Geyer R: **Biosynthesis of sulfated glycoprotein-N-glycans present in recombinant human tissue plasminogen activator**. *Biochemical and Biophysical Research Communications* 1992, **189**(3):1681-1685.

7. Wang X, Proud CG: **The mTOR Pathway in the Control of Protein Synthesis**. *Physiology* 2006, **21**(5):362-369.

8. Pastushkova LK, Kireev KS, Kononikhin AS, Tiys ES, Popov IA, Starodubtseva NL, Dobrokhotov IV, Ivanisenko VA, Larina IM, Kolchanov NA, Nikolaev EN: **Detection of Renal Tissue and Urinary Tract Proteins in the Human Urine after Space Flight**. *PLOS one* 2013, **8**(8):e71652.

9. Larina IM, Kolchanov NA, Dobrokhotov IV, Ivanisenko VA, Demenkov PS, Tiys ES, Valeeva OA, Pastushkova LK, Nikolaev EN: **Reconstruction of associative protein networks connected with processes of sodium exchange regulation and sodium deposition in healthy volunteers based on urine proteome analysis**. *Hum Physiol* 2012, **38**(3):316-323.

10. Liu X, Yu X, Zack D, Zhu H, Qian J: **TiGER: A database for tissue-specific gene expression and regulation**. *BMC Bioinformatics* 2008, **9**(1):271.
